# The linkage-type and the exchange molecule affect the protein-labeling efficiency of iminoboronate probes†

**DOI:** 10.1039/d3ob01269g

**Published:** 2023-11-07

**Authors:** Antonie J. van der Zouwen, Aike Jeucken, Elske van der Pol, Gerben Boerema, Dirk J. Slotboom, Martin D. Witte

**Affiliations:** a Chemical Biology II, Stratingh Institute for Chemistry Nijenborgh 7 9747 AG Groningen The Netherlands m.d.witte@rug.nl; b Membrane Enzymology, Groningen Biomolecular Sciences and Biotechnology Institute 9747 AG Groningen The Netherlands

## Abstract

Reversible bioorthogonal conjugation reactions have been exploited in the chemoproteomic field to prepare protein labeling reagents and to visualize labeled proteins. We recently demonstrated that reversible iminoboronates can be used to prepare probes from fragment libraries and that the linkage subsequently can be used to detect the labeled proteins. In this study, we determined the effect of the stability of the iminoboronate linkage on the efficiency of the labeling protocol. Our study reveals that the linkage should be stable enough to allow for efficient targeting, but should be labile enough to detect the labeled protein. Acyl hydrazides were identified as the most suitable handles for the probe synthesis step. Anthranilic hydrazides and *N*-hydroxy semicarbazides were found to be the most efficient read-out molecules. With these novel exchange molecules, native probe-labeled proteins could be visualized under physiological conditions.

## Abstract 2

Acyl hydrazides are the most suitable substrates for the synthesis of iminoboronate probes. They have the appropriate balance between stability, which is required for targeting, and reversibility, which is required for detecting the labeled proteins.

## Graphical abstract

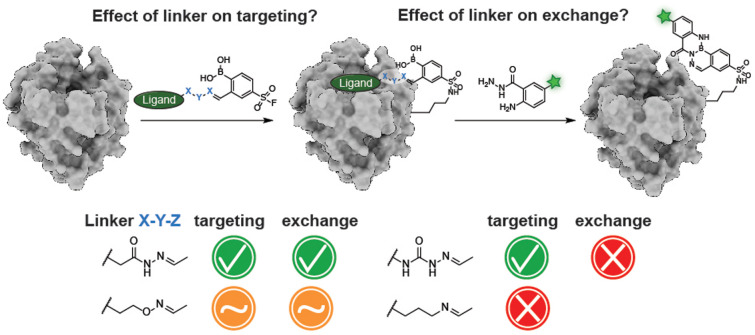

## Introduction

Conjugation reactions allow the linking of two (bio)molecules and have been applied in the chemoproteomics field to couple (photo)crosslinkers to peptides, reversible inhibitors, small molecule ligands, fragment libraries, and even proteins.^1–6^ The resulting protein labeling reagents—so-called affinity- and activity-based probes—covalently modify their targets. Detecting the probe-modified proteins often requires a second conjugation reaction between a bioorthogonal handle on the probe and a reporter group.^7,8^ In most conventional chemoproteomic approaches two conjugation handles are used, one for each step.

**Fig. 1:**
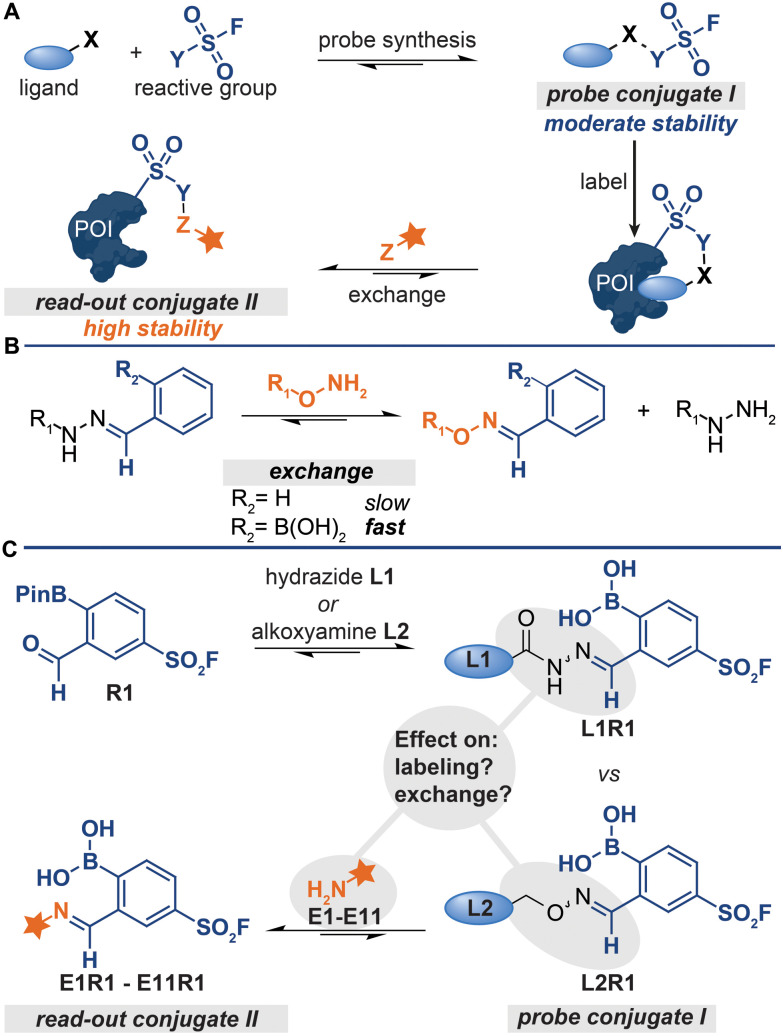
(A) Schematic representation of probe formation *via* reversible bioorthogonal reactions. *X*, *Y* and *Z* represent bioorthogonal handles. (B) Difference between the reactivity of regular hydrazones and iminoboronates. (C) This work studies the effect of the iminoboronate-linkage type and the exchange molecule on the labelling and exchange efficiency.

Both the synthesis of probe conjugate I and the detection of probe-labeled proteins can be performed with a single ligation handle (Fig. 1A), provided that the conjugation reaction is reversible.^5,6,9,10^ In that case, read-out conjugate II can be prepared from probe conjugate I by simply exchanging the protein-binding ligand for a reporter group. This strategy simplifies the synthesis and screening of probe libraries, especially when the conjugation reactions have a broad functional group tolerance.^6^ Conjugation reactions that form non-invasive by-products can even be used for the development of direct-to-biology probe synthesis approaches,^11^ which is particularly advantageous for the discovery of probe leads and the optimization of probes.

A prerequisite for detecting the labeled proteins with the strategy depicted in Fig. 1A is that conjugates of increasing thermodynamic stability are formed. Read-out conjugate II should be more stable than probe conjugate I to drive the ligand-exchange reaction. Therefore, probe conjugate I should not be too stable, as high stability of I will lower the overall efficiency of the protocol. On the other hand, probe conjugate I should also not be too labile. Hydrolysis of the probe conjugates in the labeling step leads to the loss of the targeting ligand and thus to less efficient labeling of the protein of interest.

The kinetic stability of probe conjugate I should be taken into consideration as well. Hydrazone and oxime formation were initially used for the probe synthesis and the read-out steps respectively,^5,6,9,10^ because both reactions are bioorthogonal and can be performed on chemically modified proteins.^12,13^ While probes were indeed readily prepared by reacting acyl-hydrazide ligands with a diverse set of reactive groups, the read-out of the labeled proteins by exchanging the ligand with fluorophore-alkoxyamine proved to be rather challenging.^5,6,9^ The oxime conjugate is thermodynamically more stable,^14,15^ but the relative high kinetic stability of the acyl hydrazone necessitated the use of forcing conditions (100 equivalents of the fluorophore at an acidic pH of 5.5).^5^

We recently demonstrated that problems associated with the high kinetic stability of hydrazones can be overcome by using iminoboronate chemistry for the synthesis and read-out steps.^11^ Iminoboronate chemistry has emerged as a faster and more reversible alternative to conventional hydrazone chemistry.^16–19^*ortho*-Boronic acid groups make imines kinetically more labile. Therefore, the iminoboronate adducts formed by 2-formylphenylboronic acid (2-FPBA) and 2-acetylphenylboronic acid (2-AcPBA) typically undergo exchange reactions more readily than their benzaldehyde and acetophenone counterparts (Fig. 1B).^17^ In our proof-of-concept studies, we prepared chemical probes by reacting hydrazide or alkoxyamine-functionalized ligands with 2-FPBA-based sulfonyl fluoride R1 (Fig. 1C) and we detected the labeled proteins by exchanging the ligand with α-amino hydrazide fluorophore **FITC amzide**.^11^ The iminoboronate chemistry improved the overall efficiency of the protocol considerably.

For our proof-of-concept study, we selected the respective iminoboronate adducts for the probe formation and the read-out based on the dissociation constants reported in literature.^20–22^ At the time, knowledge about how the stabilities of the different adducts compare against one another, particularly in the context of the exchange reaction, was still rather limited. Thus, it was not known how the stability of the iminoboronate probe conjugate and the read-out conjugate affected the efficiency of the various steps of the protocol (Fig. 1C). Our proof-of-concept study was also not conclusive. This was in part caused by the fact that we only determined the overall efficiency of the protocol – the signals in the fluorescence scan showed the combined result of all steps of the protocol.^11^ Moreover, mixed results were obtained for which type of iminoboronate complex was more suited for probe formation. For certain proteins, the probes formed of alkoxyamine ligands gave the strongest labeling, while for other proteins the acyl hydrazide ligands outperformed the alkoxyamine ligands. Also, only α-aminohydrazides were tested as reporter molecules in the exchange reaction.^11^

A more thorough understanding of how the iminoboronate linkage affects the probe formation and the exchange reaction, and how these combined aspects contribute to the overall efficiency of the protocol, was needed to generalize the protocol. We here demonstrate that selecting the appropriate iminoboronate conjugation reaction is key to the success of the modular probe synthesis approach. The type of iminoboronate linkage has a strong effect on the labeling efficiency. Furthermore, we screened a panel of exchange reagents and identified several compounds that have an improved exchange efficiency. The optimized procedure was employed to prepare a small panel of carbonic anhydrase probes that successfully labeled endogenously expressed human carbonic anhydrase II. The results of our studies may be exploited for the further development of direct-to-biology probe synthesis approaches, which will facilitate the synthesis and screening of probe libraries.

## Results and discussion

### Influence of the conjugation chemistry on the targeting and protein labeling efficiency

**Fig. 2:**
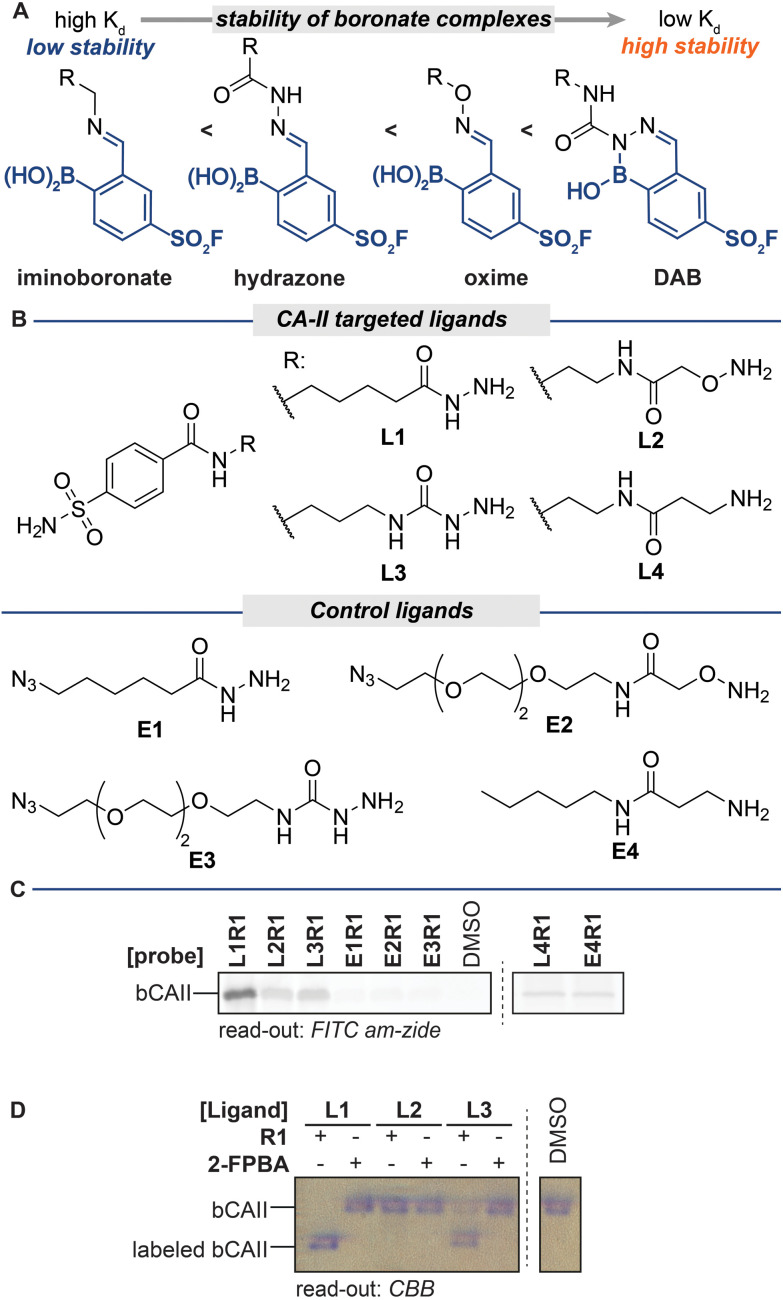
(A) Stability of various iminoboronate adducts. (B) Structures of ligands L1–L4 and E1–E4. (C) In-gel fluorescence of bCAII labeling by hydrazone-, oxime-, semicarbazone- and imine-based iminoboronate probes. Read-out with **FITC amzide**. Labeling conditions: L1R1–L4R1 (20 μM) were incubated with a mixture of bCAII (5 μM), avidin (25 μM), ovalbumin (25 μM) in HEPES (50 mM, pH 8.2) for 2 hours. Exchange: SDS denatured samples were acidified to pH 5.2 with acetic acid and incubated with **FITC amzide** for 2 hours. (D) Native PAGE as a tool to investigate the labeling efficiency. Labeled bCAII separates from unmodified bCAII on native PAGE, thus allowing assessment of the labeling efficiencies of the different probes. Read-out with Coomassie Brilliant Blue (CBB). Labeling conditions: L1R1–L3R1 (20 μM) was incubated with bCAII (5 μM) in HEPES (50 mM, pH 7.4) for 2 hours (for uncropped gels see Fig. S2†).

The stability of iminoboronate complexes depends on the amine used, and dissociation constants ranging from millimolar all the way to picomolar have been reported.^19–27^ It had been deduced that the order of stability is iminoboronate < boronohydrazones < oximes < diazaborines (DABs) (Fig. 2A).^17^ Within the DAB series, the stability of DABs is highly dependent on the hydrazine/hydrazide used for the formation. Acyl hydrazides give the least stable DABs, followed by DABs of semicarbazides. Aryl and alkyl hydrazines yield the most stable DAB adducts.

To examine how these differences in stability affect the efficiency of the different steps of the labeling protocol, we decided to prepare probes for bovine carbonic anhydrase II (bCAII) that contain iminoboronate linkages of varying stability. We synthesized acyl hydrazide L1, alkoxyamine L2, semicarbazide L3 and amine L4 (Fig. 2B) as a representative set of reagents. We combined these sulfonamide ligands, all of equal length, with sulfonyl fluoride R1 to form the corresponding iminoboronate probes L1R1–L4R1. As controls for ligand-independent labeling, we also prepared non-targeted probes from analogs that lack the bCAII binding motif (E1–E4). To determine the overall effect of these iminoboronate linkages on the efficiency of the protocol, we reacted the probes and controls with bCAII (5 μM in HEPES pH 8.2) for two hours. The protein samples were then denatured, acidified to pH 5.2, and incubated with **FITC amzide** (three equivalents compared to the probes) for two hours, after which the samples were separated on an SDS-PAGE gel. The in-gel fluorescence scan showed that, of L1R1–L4R1, the iminoboronate probe deriving from acyl hydrazide L1 gave the strongest fluorescent labeling of bCAII (Fig. 2C), followed by the probes deriving from alkoxyamine L2 and semicarbazide L3. All of these probes labeled bCAII in a ligand-dependent manner, as the fluorescent signals observed for the non-targeted control probes E1R1–E3R1 were considerably lower than those of the corresponding targeted version. A fluorescent signal was also visible in the sample that was treated with the probe formed of amine L4. However, similar levels of bCAII labeling were observed in the control sample that was treated with E4R1, which indicates that labeling in these samples occurs irrespective of whether a ligand is present. The reported dissociation constants for iminoboronate complexes formed of amines are in the millimolar range,^17^ which is well above the probe concentration used in the labeling and therefore we conclude that iminoboronate complex L4R1 does not form efficiently and/or that it hydrolyzes before it engages with bCAII.

The labeling experiment with L1–L4 confirmed that the stability of the iminoboronate linkage used for probe formation influences the labeling outcome. Only probes formed of ligands that yield relatively stable iminoboronate complexes (acyl hydrazides, alkoxyamines, semicarbazides) labeled bCAII in a ligand-directed manner. Labile iminoboronate adducts, as in L4R1, do not form efficiently enough and hydrolyze too quickly for target engagement to occur and are not suitable for probe formation.

While increased stability of the iminoboronate linkage clearly is beneficial for targeting of the probe, it could reduce the efficiency of the ligand-fluorophore exchange step, thereby leading to a net reduction in fluorescent labeling. Therefore, our next step was to assess how the iminoboronate linkage affected the efficiency of the exchange reaction. We could not solely use the fluorescence intensity for this, as this output reflects the overall efficiency of the protocol.

**Fig. 3:**
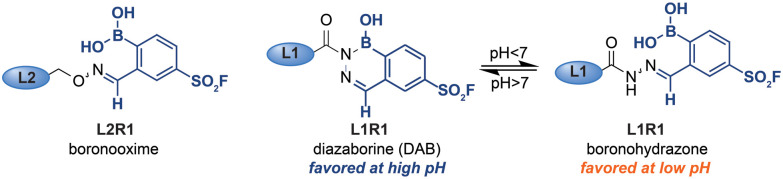
Boronooxime adducts, boronohydrazone adducts and diazoborine adducts formed by alkoxyamine and hydrazide ligands.

In order to assess the influence of the iminoboronate complex on the exchange reaction, we first had to determine the efficiency of L1R1–L3R1 in the protein labeling step. For this, we needed an analysis method that decoupled the read-out for the protein–probe binding event from the transimination reaction. We reasoned that, upon covalent attachment of the probe, there might be a separation between the covalently modified and the non-modified bCAII on native PAGE, as the iminoboronate complexes introduces an additional negative charge under the electrophoresis conditions. The resulting gel shift might be used to establish the labeling efficiency. Gratifyingly, bCAII (5 μM) labeled with L1R1 or L3R1 indeed showed a distinct shift from DMSO-treated bCAII on native PAGE gel (Fig. 2D). Incubating bCAII with control probes formed of L1 or L3 and 2-FPBA did not induce this shift. These control samples confirmed that the shift observed for L1R1 or L3R1 stemmed from covalent modification of bCAII by the sulfonyl fluoride in R1, rather than non-covalent interactions between the iminoboronate probes and bCAII. Therefore, we were confident that native PAGE was suitable to determine the efficiency of the first steps of the labeling protocol. All bCAII had been covalently labeled by L1R1. In the samples treated with L3R1, some unmodified bCAII was still present, indicating that these probes modify bCAII, but less efficiently. Interestingly, treatment of bCAII with the oxime probe L2R1 did not result in a shift at all. Apparently, labeling of bCAII with L2R1 is very inefficient. A potential explanation for the selectivity differences is that, although the probes had the same linker length, their 3D-conformations are not identical. Alkoxyamines yield extended oxime products, while acyl hydrazides give iminoboronate complexes that are, depending on the pH, either in the open boronohydrazone or in the cyclic DAB (Fig. 3).^20^ Accordingly, the positioning of the sulfonyl fluoride reactive group will be different for the DAB probes and boronooxime probes, and these differences in 3D-conformation of L2R1 compared to the other probes might be reflected in the labeling efficiency.

Finally, we compared the outcome of the fluorescent labeling assay with that of the gel shift assay, which revealed that the fluorescence output only partly correlated with the labeling efficiency. Hydrazone L1R1 labeled bCAII most efficiently and, as expected, gave the most intense signal in the fluorescence scan. The fluorescent signal in the sample that was treated with the probe deriving from semicarbazide L3 was considerably weaker (Fig. 2C), even though the labeling efficiency of L3R1 was only slightly less than L1R1, according to the gel shift assay (Fig. 2D). In fact, the fluorescent signal for L3R1-labeled bCAII is comparable to that of L2R1, despite L2R1 being far less efficient at covalently modifying the bCAII. This discrepancy indicates that the DAB complex formed by semicarbazide L3 is less susceptible to the ligand-exchange reaction, and this leads to an overall reduced efficiency of the protocol. Moreover, it suggests that exchange of alkoxyamine ligands may be feasible, but due to the poor efficiency of oxime L2R1 in the labeling step, we could not determine how the exchange of alkoxyamines compared to acyl hydrazides. Therefore, we studied exchange reactions of oximes and boronohydrazones by NMR (Fig. S1†), which showed that oximes are considerably more stable. This result led us to conclude that out of the investigated linkers, the acyl hydrazide is most suited for the use in the iminoboronate probes, followed by alkoxyamines and semicarbazide. Amine linkers are too labile to form probes.

### Optimization of the reagent for the exchange reaction

**Fig. 4:**
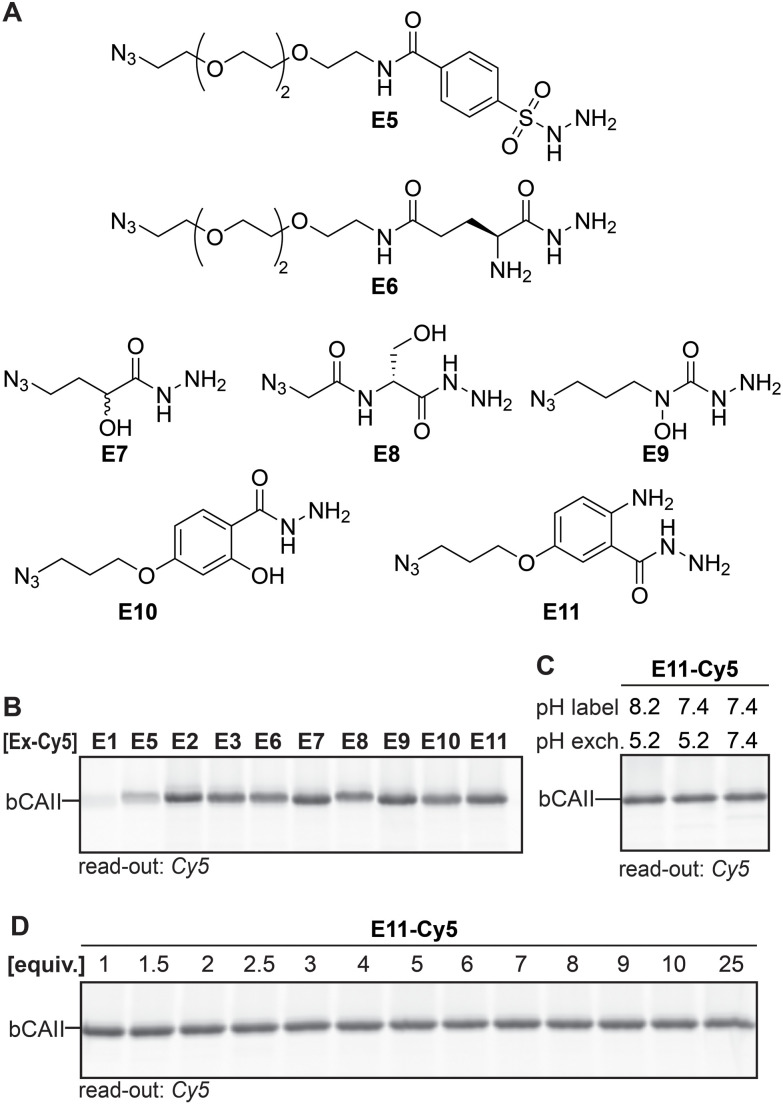
(A) α-Nucleophile reporter groups E5–E11. (B) Transimination efficiency of the different Cy5-conjugated α-nucleophile reporters on L1R1-labeled bCAII. Exchange conditions: three equivalents of exchanger, pH 5.2, overnight incubation. (C) Determination of the influence of the pH on the covalent labeling of bCAII with L1R1 and the subsequent exchange reaction. Exchange conditions: three equivalents of exchanger-Cy5, indicated pH, overnight incubation. Example given for E11 as the reporter group. (D) Influence of the number of equivalents of Cy5-conjugated reporter group (compared to probe) on the transimination reaction on L1R1-labeled bCAII. Exchange conditions: indicated equivalents of exchanger-Cy5, pH 7.4, overnight incubation. Example given for E11 (for uncropped gels and results of other exchange molecules see Fig. S3 and 4†).

Having identified the acyl hydrazide as the preferred handle to be used in the ligands, our focus shifted towards optimizing the exchange reagent used in the transimination reaction. In our original study, we exchanged the ligand with **FITC-amzide** at pH 5.2. Besides α-amino hydrazides,^20^ sulfonyl hydrazides,^28–30^ α- and β-hydroxy hydrazides,^22^ salicylic hydrazides and anthranilic hydrazides^24^ have also been reported to form stabilized DAB adducts with 2-FPBA, and we hypothesized that some of these α-nucleophiles might allow faster exchange and/or might allow the exchange reaction to occur at a physiological pH of 7.4. To validate this hypothesis, we designed and synthesized E5–E11 (Fig. 4A). In order to easily access different variants of the α-nucleophile exchange molecules, we prepared the azide-functionalized derivatives, as these could be conjugated to reporter groups of interest through a strain-promoted azide–alkyne click reaction. We also included acyl hydrazide E1, alkoxyamine E2 and semicarbazide E3 in the screening experiments.

First, we studied the exchange of the ligand with E1–E3 and E5–E11 under conditions in which transimination reaction reaches its equilibrium. E1–E3 and E5–E11 were conjugated to DBCO-Cy5 and three equivalents (compared to the amount of probe) of the resulting Cy5 constructs were added to heat-denatured, L1R1-labeled bCAII. The pH was adjusted to pH 5.2 and the resulting samples were incubated for twenty-two hours. The fluorescence signals for E2, E3 and E6–E11 were very similar, but E2, E3, E7 and E9–E11 gave a slightly stronger signal (Fig. 4B). It was expected that the differences would be minimal, since the iminoboronate complexes of E2, E3 and E6–E11 are more stable than the acyl hydrazone in L1R1. Stability studies further confirmed that the majority of the adducts are highly stable, in particular those formed by E9–E11 (Fig. S5†). Exchange with the acyl hydrazide E1 and the sulfonyl hydrazide E5 was inefficient and therefore, these reagents were excluded from further studies.

When the same procedure was carried out at pH 7.4, rather than at pH 8.2 (labeling reaction) and 5.2 (transimination) as had been the case so far, no visible difference in fluorescent intensity were observed between the different conditions. Pleasingly, this results indicates that exchange reaches its equilibrium at pH 7.4 after twenty-two hours of incubation. Moreover, it shows that the labeling step can be carried out at physiological pH as well (for example see Fig. 4C). Finally, the amount of exchange molecule that was necessary for an optimal exchange reaction was evaluated. bCAII was labeled with L1R1, denatured using SDS and subsequently treated with varying amounts of the Cy5-adducts of E2, E3, and E6–E11 for twenty-two hours at pH 7.4. For all molecules, the maximum in-gel fluorescence intensity was already obtained when using one equivalent (compared to the amount of L1R1 used; for an example see Fig. 4D).

**Fig. 5:**
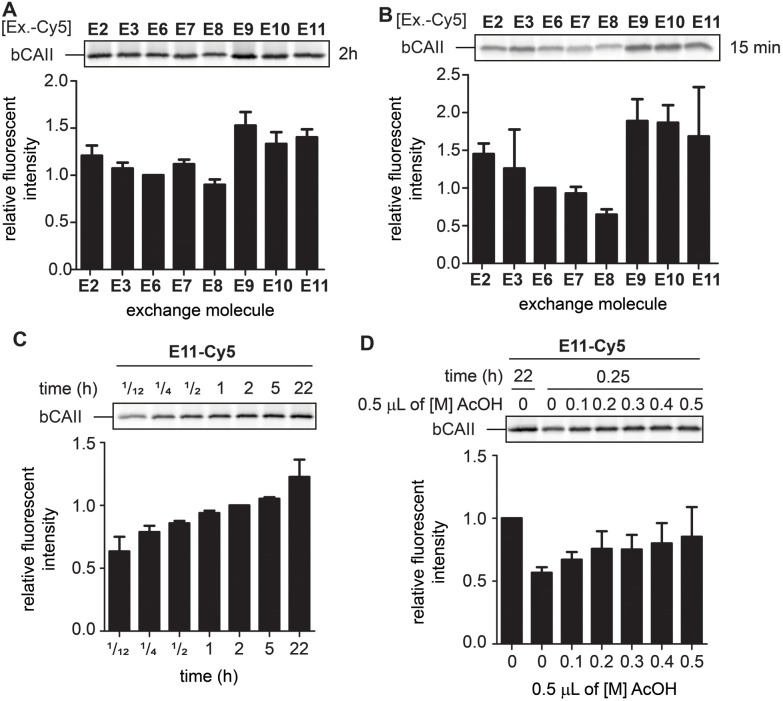
(A) Relative fluorescence obtained after 2 hours of exchange reaction. Normalized for the fluorescent signal of E6. The experiments were carried out in quadruplicate. (B) Relative fluorescence obtained after 15 minutes of exchange reaction. Normalized for the fluorescent signal of E6. The experiments were carried out in quadruplicate. (C) Relative fluorescence of the exchange reaction over time. Example given for E11. Normalized for the fluorescent signal obtained after 2 hours. The experiments were carried out in quadruplicate. (D) Influence of lowering the pH *via* addition of increasing concentrations acetic acid (AcOH) on an exchange reaction of 15 minutes. Example given for E11. Normalized for the fluorescent signal obtained for an exchange reaction of 2 hours in the absence of acetic acid. The experiments were carried out in quadruplicate. Labeling conditions: L1R1 (20 μM) was incubated with a mixture of bCAII (5 μM), avidin (25 μM), ovalbumin (25 μM) in HEPES (50 mM, pH 7.4) for 2 hours (for uncropped gels and results with E2, E3 and E9 see Fig. S6 and S7†).

While the differences between E2, E3 and E6–E11 are minimal after twenty-two hours, we expected that they would behave differently at earlier time points when the equilibrium is not yet reached. At these time-points, the α-nucleophiles that exchange the ligand the fastest should give the strongest labeling. Incubating L1R1-labeled bCAII with one equivalent of the Cy5-conjugates of E2, E3 and E6–E11 at pH 7.4 for either fifteen minutes or two hours indeed led to prominent differences in signal intensities (Fig. 5A and B). The relative fluorescent intensities – normalized to α-aminohydrazide E6 – were the highest for E9, E10 and E11, and the difference was most pronounced after fifteen minutes of transimination. These results indicated that E9, E10 and E11 undergo the exchange reaction the quickest.

To study how the reaction time and the pH affected the transimination efficiency in further detail, we focused on E2, E3, E9 and E11 as a representative set of the different transimination efficiencies observed. Varying the reaction time between five minutes and twenty-two hours revealed that the transimination reaction did not yet reach its maximum after incubating for two hours at pH 7.4. However, at this time point, the exchange is only slightly less efficient than after twenty-two hours (Fig. 5C and S7†). This means that the time for the transimination could be shortened while still obtaining a decent fluorescent signal. Furthermore, we found that, although the pH did not have a pronounced effect on the signal intensity after twenty-two hours (Fig. 4D), lowering of the pH did result in differences in earlier time points (Fig. 5D and S7†). Acidification of the samples increased the exchange rate and allowed shortening the reaction time to 15 minutes. From all these studies, we conclude that exchangers E9 and E11 are particularly suited for the transimination reaction. Judging from the gel shift in the Coomassie Brilliant Blue stained gels, the exchange efficiency is >50% (Fig. S7†). Depending on the experimental set-up, the reporter group can either be introduced by exchanging the ligand with one equivalent of exchange molecule (compared to the probe) at pH 7.4 for two hours or at pH 5 for 15 minutes.

**Fig. 6:**
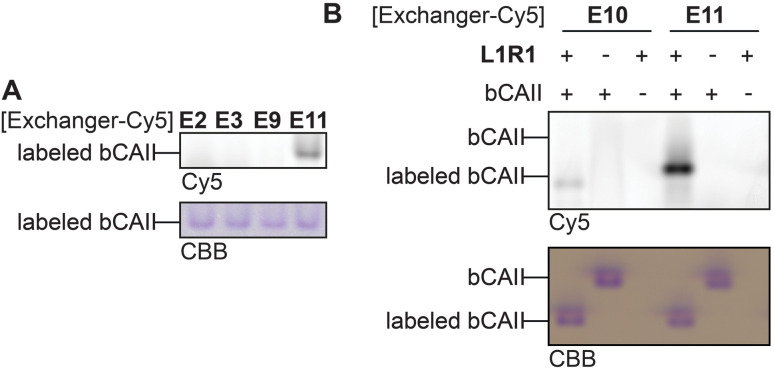
(A) Exchange reaction of E2, E3, E9 and E11 on native, L1R1-labeled bCAII. (B) Treatment of native bCAII with L1R1 or DMSO, followed by an exchange reaction with E10 and E11. Labeling conditions: L1R1 (20 μM) was incubated with bCAII (5 μM) in HEPES (50 mM, pH 7.4) for 2 hours. Exchange: exchanger-Cy5 for 2 hours (for uncropped gels see Fig. S8†).

Thus far, the exchange reaction was tested on denatured protein. For cell-based applications, it should also work on native proteins. To determine if transimination on native proteins was feasible, we carried out the exchange reaction on folded L1R1-modified bCAII with E2, E3, E9, E10 and E11. To ensure maximum transimination, we allowed the protein to react for twenty-two hours. The samples were then subjected to native PAGE and the in-gel fluorescence was analyzed (Fig. 6). The results showed that only E10 and E11 were able to perform the transimination on native bCAII, with E11 being more efficient. Incubating unlabeled bCAII with either E10 or E11 did not result in a fluorescent signal, showing that the observed signal was not caused by non-specific interactions between the bCAII and E10 or E11. Based on these and all previous findings, it was concluded that the anthranilic hydrazide E11 is the α-nucleophile of choice for the transimination reaction when performing the exchange on native proteins.

### Profiling of cell lysates with iminoboronate probes

**Fig. 7:**
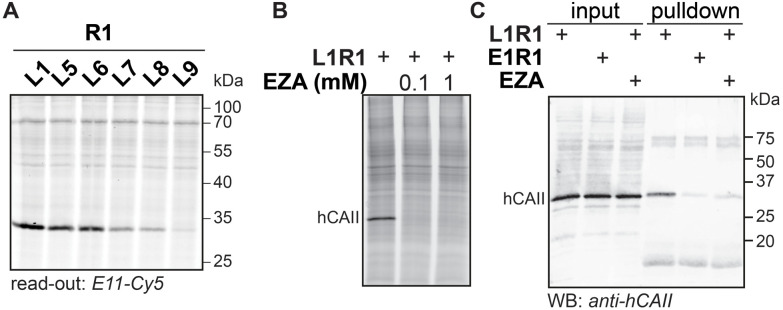
(A) Labeling with R1-derived probes in HEK293 lysate. HEK293 cell lysate (2 mg mL^−1^) in HEPES (50 mM, pH 7.4) was incubated with R1-based iminoboronate probes (1 μM) for 2 hours. Read-out with **E11-Cy5** (1 μM). (B) Labeling with L1R1 (10 μM) in the presence of ethoxzolamide (EZA) (100 μM or 1 mM). Read-out with **E11-Cy5** (10 μM). (C) Western blot analysis of material retrieved from HEK lysate that was labeled with L1R1 or E1R1 (10 μM) and subsequently subjected to an exchange reaction with **E11-biotin**. Input: aliquot taken before neutravidin enrichment of the labeled proteins; pulldown: material retrieved from the neutravidin beads. Read-out: immunoblot; primary antibody: rabbit polyclonal anti-human carbonic II; secondary antibody: donkey anti-rabbit 680.

To demonstrate the applicability of anthranilic hydrazide E11 in profiling chemical probes, we prepared iminoboronate probes of sulfonamide ligands L1, L5, L6, L7, L8 and L9 with the 2-FPBA-derived reactive groups, sulfonyl fluoride R1 and epoxide R2 (Fig. S9†), and we used these probes to profile carbonic anhydrases in HEK293 cell lysate. To this end, the cell lysate (2 mg mL^−1^ in HEPES pH 7.4) was treated with 1 μM probe for two hours, followed by SDS denaturing of the lysate and subsequent transimination with one equivalent of E11 for two hours. The lysates were separated on SDS-PAGE and the labeled proteins were visualized by scanning the in-gel fluorescence. The results for the profiling showed that the sulfonamide probes L1R1, L5R1, L6R1, L7R1, L8R1 and L9R1 profoundly labeled a protein band at ∼30 kDa (Fig. 7A). Labeling of this band was also observed when the same sulfonamide ligands were combined with epoxide R2, although the intensity of the fluorescent signal was lower (Fig. S9†). E9 could be used as read-out as well (Fig. S9†). We suspected the protein to be human carbonic anhydrase 2 (hCAII), as this protein has a molecular weight of 29 kDa, is highly expressed in HEK293 cells and is known to be targeted by sulfonamide ligands. Also, labeling by L1R1 was blocked in the presence of the known carbonic anhydrase inhibitor ethoxzolamide (EZA) (Fig. 7B).^31^ To further confirm that the labeled protein corresponds to hCAII, we labeled the protein with L1R1, exchanged the sulfonamide ligand with **E11-biotin** and enriched the biotinylated proteins with neutravidin beads. The retrieved material was analyzed with western blot using anti-hCAII for read out. The blot revealed that the hCAII signal is stronger in the sample that was labeled with L1R1 compared to the samples that were treated with the control probe E1R1 or that were pretreated with ethoxzolamide.

## Experimental

### General biochemical procedures

Ligands, reactive groups and exchanger molecules were dissolved in DMSO in 50 mM stocks and stored at −20 °C. Probe formation was carried out by mixing equimolar amounts of ligand and reactive group and incubation for 30 minutes, followed by dilution to the desired concentrations. Probe solutions were stored at −20 °C and could be used for ≥2 months. DBCO-Cy3 (Sigma-Aldrich 777366), DBCO-Cy5 (Sigma-Aldrich 777374), were purchased from commercial vendors. Laemmli sample buffer (4×) contained Tris (pH 6.8, 200 mM), SDS (8% w/v) bromophenol blue (0.2% w/v), glycerol (40%) and β-mercaptoethanol (20%). Native sample buffer (2×) contained Tris (pH 6.8, 62.5 mM), glycerol (40%) and bromophenol blue (0.025% w/v).

### Cy3-, Cy5- and biotin-conjugated exchanger molecules

The azide-containing exchanger molecules (2 μL of 50 mM) were reacted with DBCO-Cy3, DBCO-Cy5 or DBCO-biotin (2.4 μL of 50 mM) for 22 hours. Any unreacted DBCO was quenched by addition of 2-azido-*N*,*N*,*N*-trimethylethylammonium iodide (3.6 μL of 100 mM) and incubation for 22 hours. The reporter-conjugated exchanger molecules were diluted to the desired concentrations with DMSO and used without any further purification. The molecules were stored at −20 °C and could be used for ≥4 months.

### Labeling of bovine carbonic anhydrase II with iminoboronate probes

#### Screening of different sulfonamide-based α-nucleophile ligands

Probe (0.5 μL of 200 μM) was incubated with a mixture of bCAII/avidin/ovalbumin (4.5 μL of 5.55 μM bCAII, 27.78 μM avidin, 27.78 μM ovalbumin) in HEPES (50 mM, pH 8.2) for 2 hours. SDS (0.3 μL of 20% w/v solution in water) was added and the samples were heated at 100 °C for 15 minutes. The samples were shortly centrifuged, followed by addition of FITC amzide (0.5 μL of 600 μM) and acetic acid (0.5 μL of 500 mM), and the samples were incubated for 2 hours. Laemmli sample buffer (6.5 μL of 2× stock) was added, the samples were loaded on 12% SDS-PAGE gel, resolved and analyzed by scanning the in-gel fluorescence.

#### Labeling on native bCAII

Probe (0.5 μL of 200 μM) was incubated with bCAII (4.5μL of 5.55 μM bCAII) in HEPES (50 mM, pH 7.4) for 2 hours. Native sample buffer (5 μL of 2× stock) was added, the samples were loaded on 12% native PAGE gel, resolved and stained with Coomassie Brilliant Blue.

#### Optimized procedure for labeling with the new α-nucleophile reporters

Probe (0.5 μL of 200 μM) was incubated with a mixture of bCAII/avidin/ovalbumin (4.5 μL of 5.55 μM bCAII, 27.78 μM avidin, 27.78 μM ovalbumin) in HEPES (50 mM, pH 7.4) for 2 hours. SDS (0.3 μL of 20% w/v solution in water) was added and the samples were heated at 100 °C for 15 minutes. The samples were shortly centrifuged, followed by addition of the Cy5-conjugated exchanger molecules (0.5 μL of 200 μM), and the samples were incubated for 2 hours. Laemmli sample buffer (6 μL of 2× stock) was added, the samples were loaded on 12% SDS-PAGE gel, resolved and analyzed by in-gel fluorescence.

#### Probe labeling and exchange reaction on native bCAII

Probe (0.5 μL of 200 μM) was incubated with bCAII (4.5μL of 5.55 μM bCAII) in HEPES (50 mM, pH 7.4) for 2 hours. The Cy5-conjugated exchanger molecules (0.5 μL of 200 μM) were added and the samples were incubated for 2 hours. Native sample buffer (5 μL of 2× stock) was added, the samples were loaded on 12% native PAGE gel, resolved and stained with Coomassie Brilliant Blue.

### Labeling in HEK293 cell lysates

HEK293 cell pellets were kindly provided by Valeria Kalienkova. The pellets were lysed using a NP40 lysis buffer (0.5% NP40, Tris·HCl (10 mM), NaCl (150 mM), MgCl_2_ (5 mM), pH 7.4) during 10 min over ice, the cell debris was removed by centrifuging (10 minutes, 10 000 rpm), and the cell lysate (5.29 mg mL^−1^) was frozen using liquid nitrogen and stored at −80 °C. Probe (0.5 μL of 10 μM) was incubated with HEK293 lysate (4.5 μL of 2.22 mg mL^−1^) in HEPES (50 mM, pH 7.4) for 2 hours. SDS (0.3 μL of 20% w/v solution in water) and dithiothreitol (0.5 μL of 80 mM) were added and the samples were incubated for 45 minutes. Then, iodoacetamide (0.5 μL of 100 mM) was added and the samples were incubated for another 45 minutes. **E11-Cy5** (0.5 μL of 10 μM) was added and the samples were incubated for 2 hours. Laemmli sample buffer (7 μL of 2× stock) was added, the samples were loaded on 12% SDS-PAGE gel, resolved and analyzed by in-gel fluorescence.

### Validation of human carbonic anhydrase II as labeling target

#### Competition experiment

HEK293 lysate (90 μL of a 2.22 mg mL^−1^ solution) in HEPES (20 mM, 100 mM pH7.5) was briefly incubated with 6-ethoxy-2-benzothiazolesulfonamide (0.5 μL of 2 mM or 20 mM) or DMSO prior to incubating with L1R1 (1 μL of a 100 μM stock) for 4 hours. The samples were denatured, reduced and alkylated as described above and visualized with **E11-Cy5**.

#### Pulldown

HEK293 lysate (90 μL of a 2.22 mg mL^−1^ solution) in HEPES (20 mM, 100 mM pH7.5) was incubated with the probe (10 μL of 100 μM stock, final concentration: 10 μM, sample I: L1R1, sample II: E1R1, sample III: 0.5 μL of 50 mM 6-ethoxy-2-benzothiazolesulfonamide and L1R1). The mixture was incubated for 4 hours at room temperature, after which the proteins were heat-denatured with SDS (10 μL of 10% w/v solution in water). The samples were cooled to room temperature, treated with dithiothreitol (10 μL of a 80 mM stock) for 20 min followed by iodoacetamide (5 μL of a 200 mM stock) for 20 minutes. The proteins were stored at −20 °C overnight. The next day, the **E11-biotin** was added (10 μL of 100 μM stock in 0.1 M aqueous acetic acid) and the sample was incubated for 2 hours. An aliquot (12.5 μL) was taken from the sample (input) and sample buffer was added to this sample. The proteins were precipitated with chloroform/methanol precipitation. Briefly, methanol (400 μL) was added and the sample was vortexed. Next, chloroform (200 μL) was added and the sample was vortexed. Finally, water (300 μL) was added and the sample was vortexed. The sample was centrifuged at max g for 10 minutes. The top-layer was removed and discarded. To the bottom layer was added methanol (400 μL) and the sample was gently vortexed, after which it was centrifuged. The solvent was removed and the pellet was briefly dried. The proteins were redissolved in PBS containing 1% SDS (50 μL) and diluted with PBS to 1 mL. Neutravidin beads (50 μL of a prewashed slurry) were added to the sample. The samples were incubated under rotation for 2 hours. The tubes were centrifuged on a table top centrifuge. The supernatant was removed. The beads were washed twice with PBS containing 0.2% SDS (1 mL). During the washing, the beads were agitated for 10 minutes. Finally, the beads were washed with PBS (1 mL) and needled to dryness. Sample buffer (40 μL, 2×) containing biotin (20 μM) was added and the samples were boiled for 20 minutes. Next the sample was removed from the beads. The samples were loaded on a 12.5% SDS page. The proteins were transferred to a nitrocellulose membrane using wet-blotting according to the manufacturers procedure. The membrane was washed with TBS containing 0.1% Tween-20 (TBS-T) and blocked with 1% BSA in TBS-T and blotted with rabbit anti-hCAII polyclonal antibody (2 ml TBS-T, 1% BSA, 4 μL of a 1 mg mL^−1^ antibody solution, Invitrogen PA580391) at 4 °C under constant agitation overnight. The membrane was washed with TBS-T (3×, 10 minutes) after which the secondary antibody was added (donkey anti-rabbit 680). The membrane was incubated for 1 hour, washed with TBS-T (3 × 5 minutes) and visualized with a LiCOR Fc imager from Odyssey.

## Conclusions

In conclusion, we studied the impact of the iminoboronate conjugation reaction on our previously reported modular probe synthesis protocol with ligands L1–L4. The labeling studies on bCAII revealed that the stability of the conjugate determines the efficiency of probe formation, the ligand-directed targeting and the exchange reaction. Chemical probes that contain iminoboronate linkages that hydrolyze rapidly under the assay conditions (probe concentrations below their dissociation constant and competing biomolecules) label the target proteins inefficiently. Probes that form a very stable iminoboronate, as is the case for semicarbazide L3, efficiently modify the target protein, but the subsequent exchange reaction to introduce a reporter group is hampered by the stability of the linkage. In addition, we found that the iminoboronate linkage largely affects the positioning of the reactive group and thus the labeling efficiency. The tested oxime probes labeled bCAII inefficiently according to native PAGE. NMR studies confirmed previous findings that alkoxyamines form stable iminoboronate adducts that only exchange slowly with hydrazine. Since the effect of oxime linkers could not be properly determined on proteins, we for now recommend to use hydrazide ligands in the design of iminoboronate probes.

We also investigated the performance of different α-nucleophile reporter groups in the exchange reaction in further detail. It was shown that most exchange molecules performed similarly when the reaction was allowed to reach its thermodynamic equilibrium. Only acyl hydrazides (E1) and sulfonyl hydrazides (E5) performed poorly under these conditions. At earlier time points, E9–E11 performed best. Of these, only E10 and E11 were able to undergo transimination on a native protein. As E11 clearly outperformed E10 in this case, it was decided that anthranilic hydrazide E11 was the most suited α-nucleophile for future exchange reactions, but *N*-hydroxy semicarbazide E9 was also very efficient on denatured proteins.

Finally, we demonstrated that the optimized conditions could applied to prepare a small library of carbonic anhydrase probes that labeled endogenously expressed carbonic anhydrases in HEK293 cell lysates. The fact that the iminoboronate probes do not require purification, as well as the simple labeling and transimination protocol, should allow the expedient screening of probe libraries. Thus, the iminoboronate probes are promising tools to identify new protein–probe pairs, which could ultimately lead to the development of chemical probes to study under-investigated proteins of interest.

## Author contributions

A. J. v. d. Z. and M. D. W. conceived the project. A. J. v. d. Z., E. v. d. P. and G. B. synthesized the ligands and exchange molecules. A. J. v. d. Z., A. J., D. J. S. and M. D. W. designed and performed the experimental set-up for the biochemical labelling experiments. A. J. v. d. Z. and M. D. W. wrote the manuscript.

## Conflicts of interest

There are no conflicts to declare.

## Supplementary Material

OB-021-D3OB01269G-s001
