# Supplementary material for: The linkage-type and the exchange molecule affect the protein-labeling efficiency of iminoboronate probes
Source: Org Biomol Chem. 2023 Nov 7;21(46):9173–81. doi: 10.1039/d3ob01269g (PMC10686633; doi:10.1039/d3ob01269g)
Supplement: OB-021-D3OB01269G-s001 [file OB-021-D3OB01269G-s001.pdf]

Electronic Supplementary Material (ESI) for Organic & Biomolecular Chemistry.
This journal is © The Royal Society of Chemistry 2023

Supplementary information of:

The linkage-type and the exchange molecule affect the protein-labeling efficiency of iminoboronate probes

A. J. van der Zouwen[a], A. Jeucken[b], E. van der Pol[a], G. Boerema[a], D. J. Slotboom[b] and M. D. Witte[a]

[a] A.J. van der Zouwen, E. van der Pol, G. Boerema, Prof. Dr. M.D.Witte, Chemical Biology II, Stratingh Institute for Chemistry, Nijenborgh 7, 9747 AG, Groningen, The Netherlands. E-mail: m.d.witte@rug.nl

[b] Dr. A. Jeucken, Prof. Dr. D.J. Slotboom Membrane Enzymology, Groningen Biomolecular Sciences and Biotechnology Institute, 9747 AG Groningen, The Netherlands

Table of contents

		Page
**Figure S1**	NMR exchange studies	3
**Figure S2**	Uncropped images belonging to Figure 3	4
**Figure S3**	Uncropped image belonging to Figure 4 and effect of pH on the protocol	5
**Figure S4**	Effect of the equivalence on the exchange	6
**Figure S5**	Stability studies of the iminoboronate-exchange molecule complexes	8
**Figure S6**	Uncropped images of the in gel fluorescence scans depicted in Figure 5A, B	9
**Figure S7**	Effect of the pH and time on the exchange rate	10
**Figure S8**	Uncropped images of the native exchange experiment depicted in Figure 6	11
**Figure S9**	Structures of **L5-L9**, EZA and labeling results in HEK293 lysate with **R2**-probes	12
**General procedures**		12
**Synthetic procedures**		13
**References**		25
**Spectra**		27

To obtain conclusive results on the exchange efficiency of oximes, we measured the susceptibility of these complexes towards hydrazinolysis with NMR and compared it with a boronohydrazone. Boronate complexes of 2-FPBA (1 mM) and hydrazide **E1** (1 mM) or alkoxyamine **E2** (1 mM) were formed in a mixture of 10% DMSO-d_6, 10% D_2O and 80% PBS (pH 7.4) and were subsequently treated with an excess of hydrazine (40 mM). The disappearance of the initial boronate complex was followed over time (Figure S1). The ^{1}H-NMR spectra show that after five hours, the boronohydrazone had been almost fully converted into the DAB of hydrazine and 2-FPBA, while the oxime had only been partially converted.

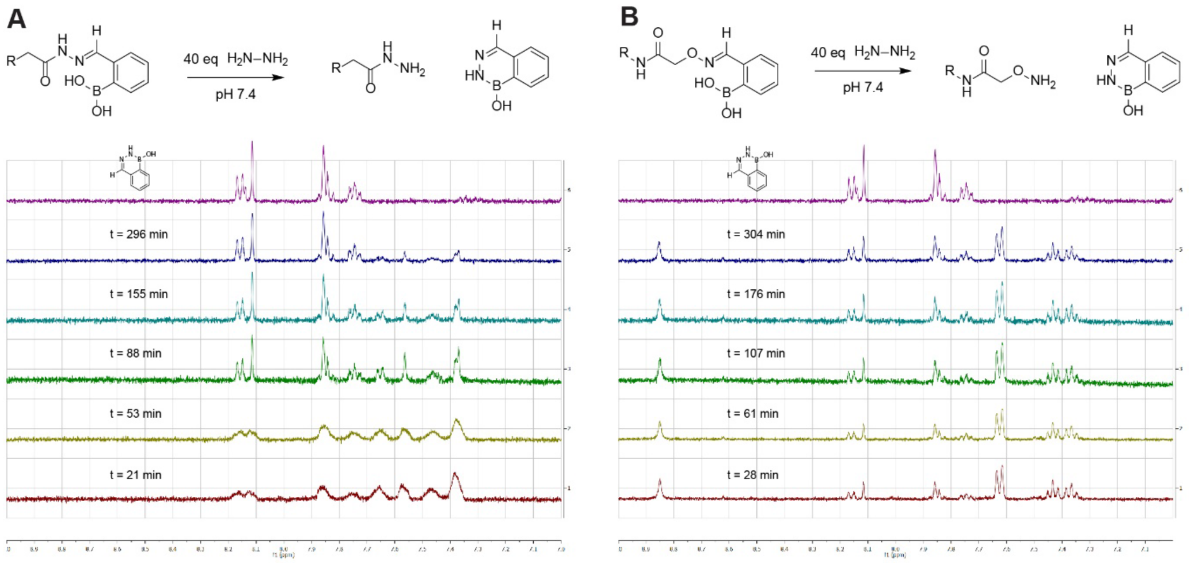

Figure S1. The stability of boronohydrazone (A) and oxime (B) adducts against hydrazinolysis. Measured by ^{1}H NMR over time.

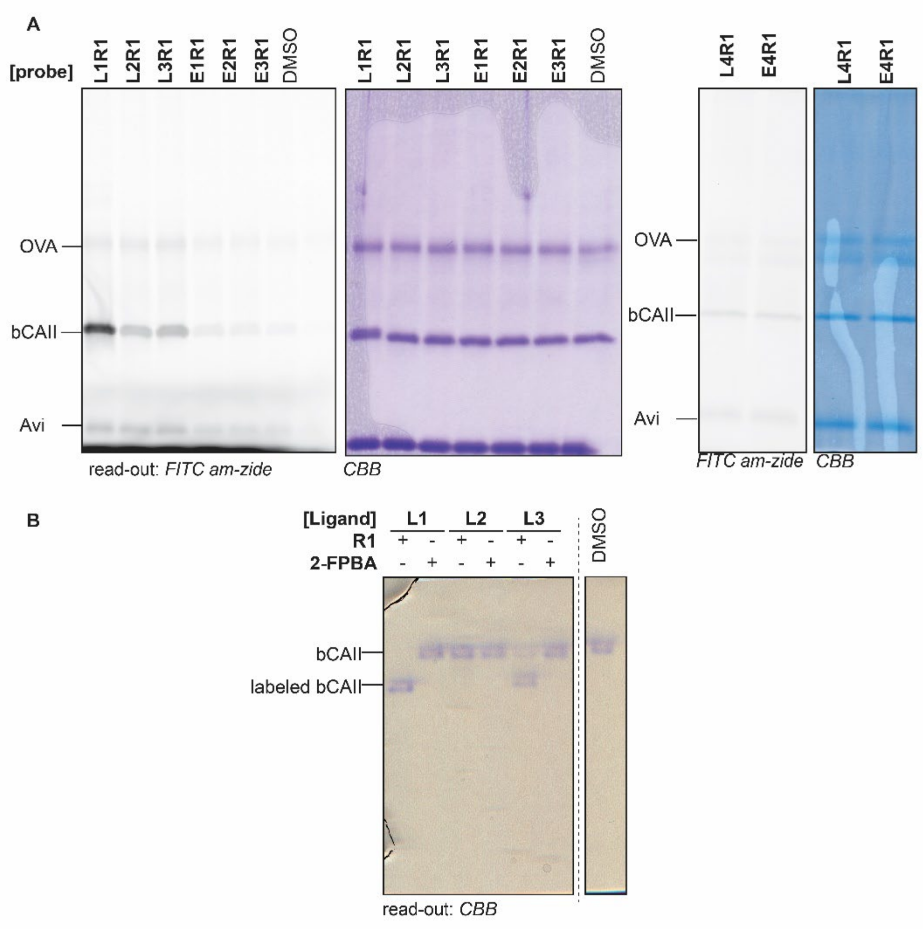

Figure S2. Uncropped images of the gels depicted in Figure 3. (A) In-gel fluorescence of bCAII labeling by hydrazone, oxime, semicarbazone and imine-based iminoboronate probes. Read-out with FITC am-zide. Labeling conditions: **L1R1-L4R1** (20 µM) was incubated with a mixture of bCAII (5 µM), avidin (25 µM), ovalbumin (25 µM) in HEPES (50 mM, pH 8.2) for 2 hours. Exchange: SDS denatured samples were acidified to pH 5.2 with acetic acid was incubated with FITC am-zide for 2 hours. (B) Native PAGE as a tool to investigate the labeling efficiency. Labeled bCAII separates from unmodified bCAII on native PAGE, thus allowing assessment of the labeling efficiencies of the different probes. Read-out with Coomassie brilliant blue (CBB). Labeling conditions: **L1R1-L3R1** (20 µM) was incubated with bCAII (5 µM) in HEPES (50 mM, pH 7.4) for 2 hours.

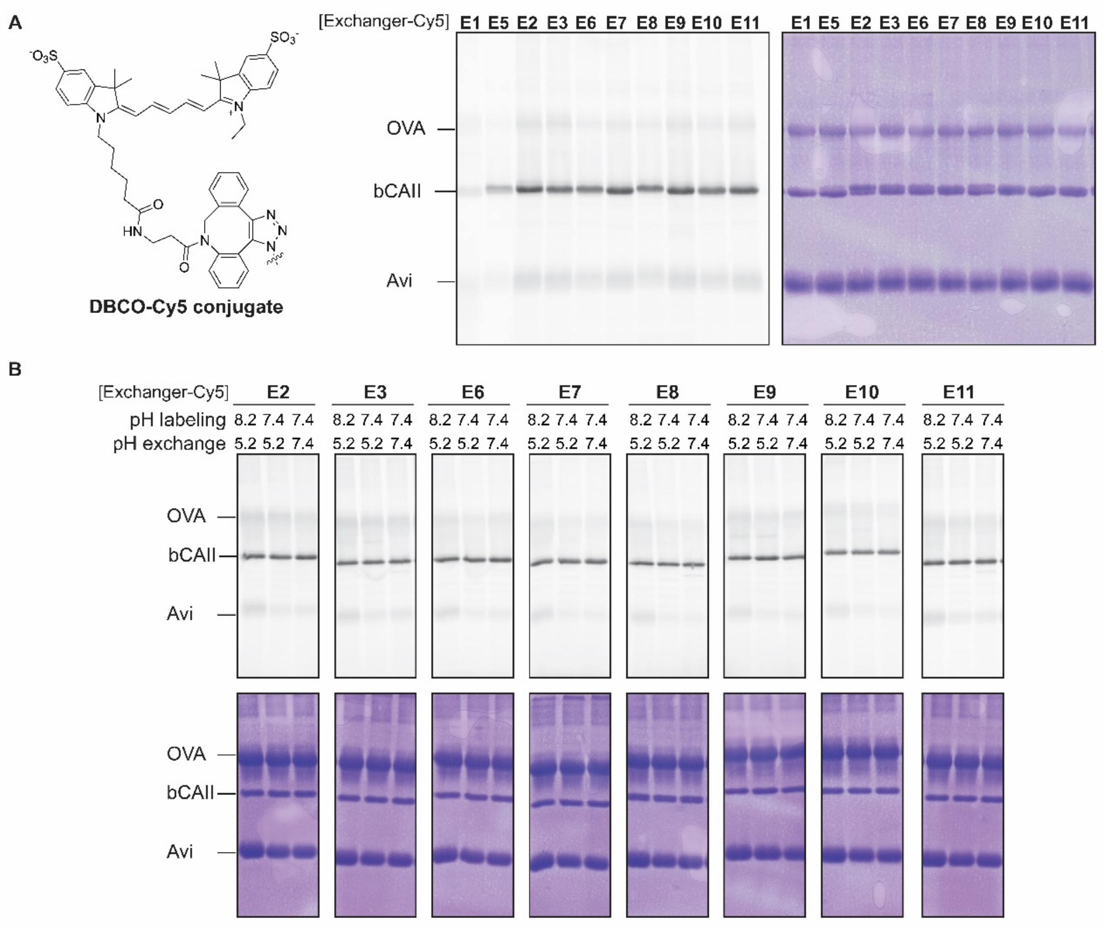

Figure S3. (A) Structure of DBCO-Cy5 conjugate and an uncropped version of the in-gel fluorescence scan depicted in Figure 3B. The transimination reaction was performed with α-nucleophile reporters **E2**, **E3**, **E6–E11** on **L3R1**-labeled bCAII. Exchange conditions: Three equivalents of exchanger, pH 5.2, overnight incubation. (B) Determination of the influence of the pH on the covalent labeling of bCAII with **L1R1** and the subsequent exchange reaction with **E2**, **E3**, **E6–E11**. Exchange conditions: Three equivalents of exchanger-Cy5, indicated pH, overnight incubation.

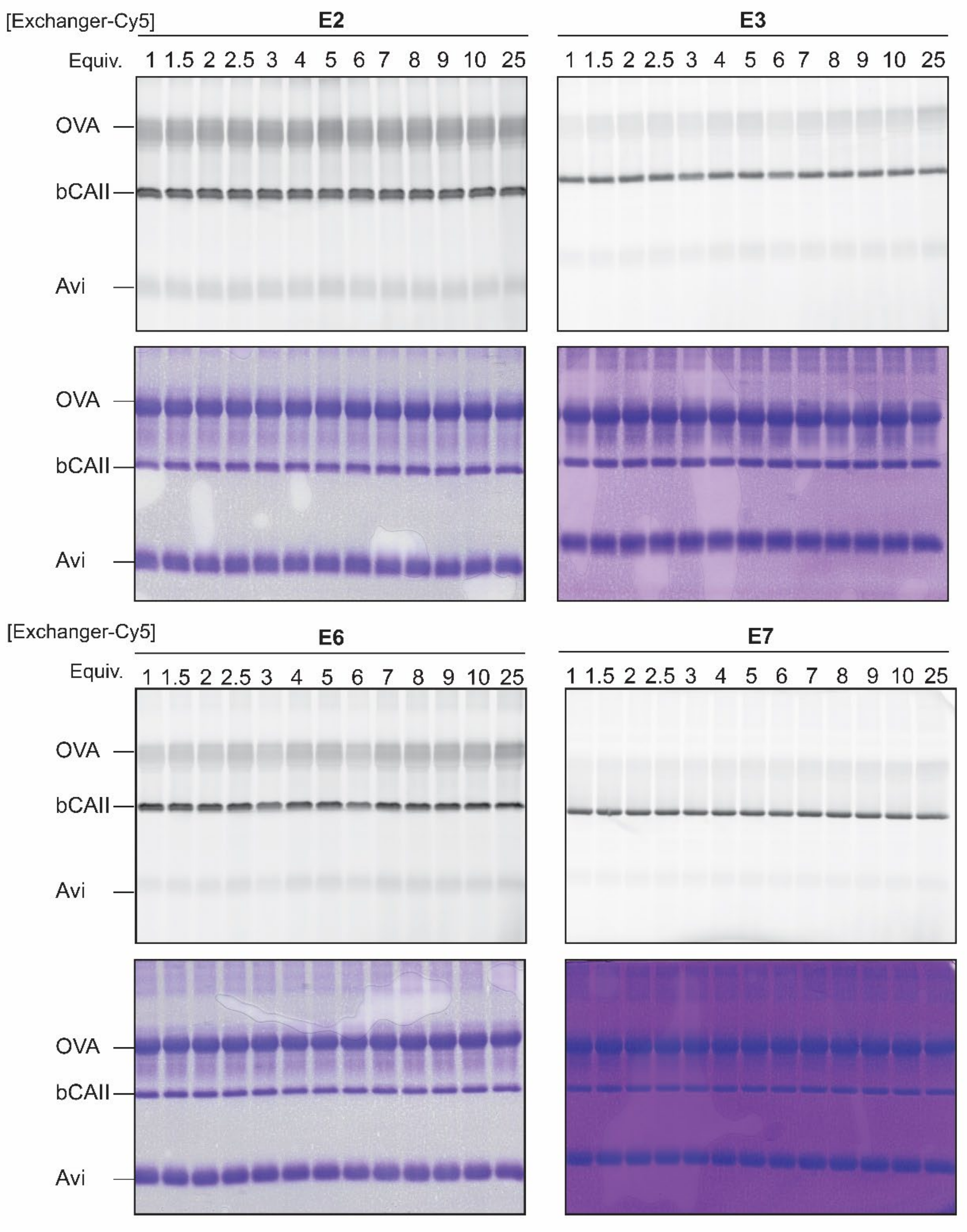

[Exchanger-Cy5]
E2
E3
Equiv.
1 1.5 2 2.5 3 4 5 6 7 8 9 10 25
1 1.5 2 2.5 3 4 5 6 7 8 9 10 25
OVA
bCAII
Avi
OVA
bCAII
Avi
[Exchanger-Cy5]
E6
E7
Equiv.
1 1.5 2 2.5 3 4 5 6 7 8 9 10 25
1 1.5 2 2.5 3 4 5 6 7 8 9 10 25
OVA
bCAII
Avi
OVA
bCAII
Avi

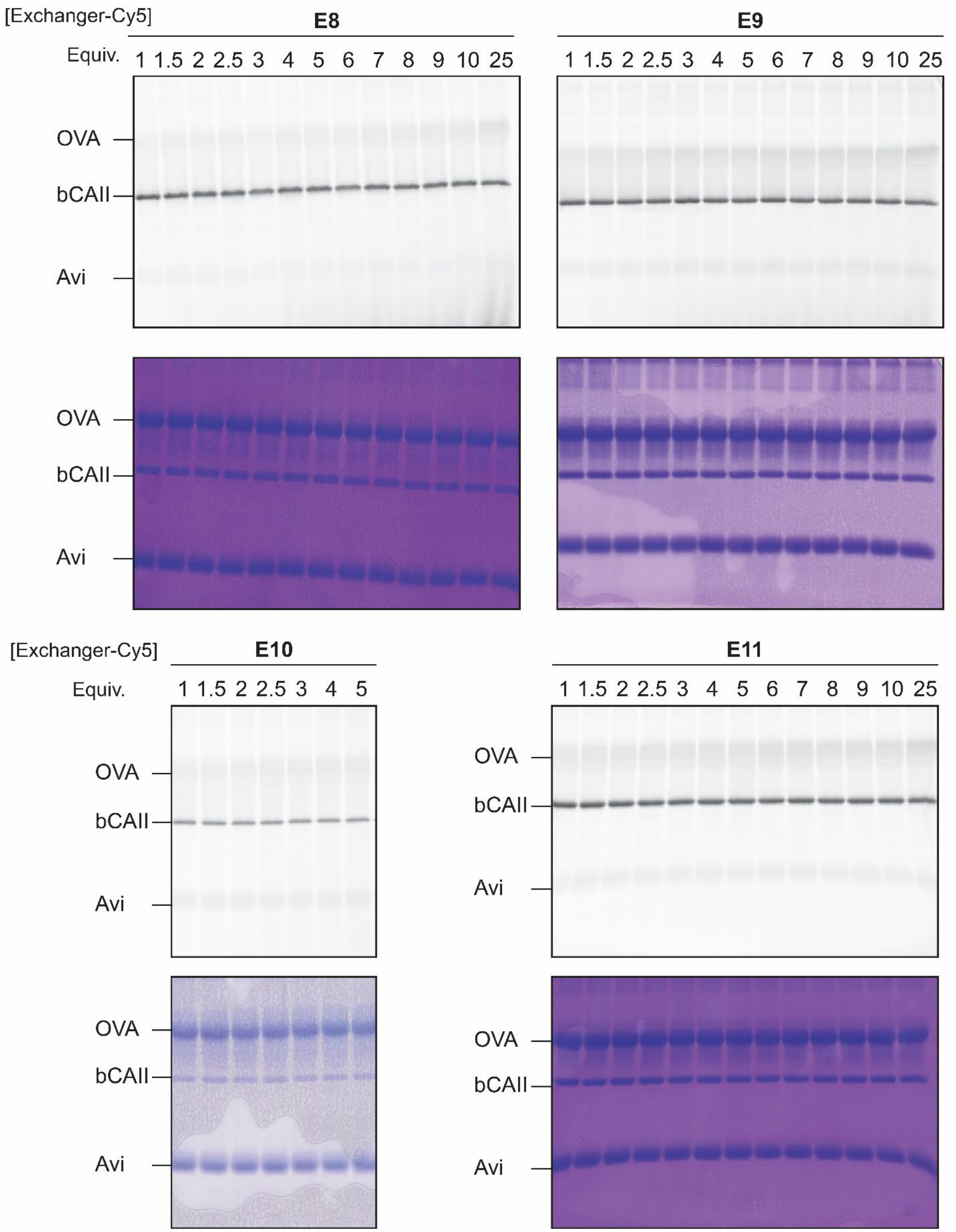

Figure S4. Influence of the number of equivalents of Cy5-conjugated reporter group (compared to probe) on the transimination reaction on **L1R1**-labeled bCAII. Exchange conditions: indicated equivalents of exchanger-Cy5, pH 7.4, overnight incubation. Read-out is the in-gel fluorescence of the Cy5 dye.

To gain insight in the stability of iminoboronate complexes formed by exchange molecules **E2**, **E3**, and **E6–E11**, we subjected samples that were labeled with **L1R1** and transiminated with **E2**, **E3**, and **E6–E11** to hydrazinolysis. This treatment should remove **E2**, **E3**, and **E6–E11** from bCAII and thus should lead to a loss in labeling signal (Figure S5A). The in-gel fluorescence showed that the signal intensity of bCAII labeled with β-hydroxy hydrazide **E8** had decreased after incubation with an excess of hydrazine for twenty-four hours at a pH of ~5.2 (Figure S5B). The changes in intensity were less profound for alkoxyamine **E2**, semicarbazide **E3**, α-amino hydrazide **E6**, α-hydroxy hydrazide **E7** and *N*-hydroxysemicarbazide **E9**, but still noticeable. Incubation with hydrazine did not have an apparent effect on the fluorescent intensity of bCAII visualized with salicylic hydrazide **E10** and anthranilic hydrazide **E11** at all, indicating that these complexes are the most stable. We investigated the hydrazinolysis of α-amino hydrazide **E6**, α-hydroxy hydrazide **E7** and β-hydroxy hydrazide **E8** in further detail, as a study by the group of Bane had shown that these iminoboronate complexes were reversible.[1] Following the hydrazinolysis over time, it was clear that the β-hydroxy hydrazide **E8** formed the most labile iminoboronate (Figure S5C). In our hands, the hydrazinolysis of **E8** was much slower than reported and the fluorescent signal decreased by roughly 50% after forty-five hours.

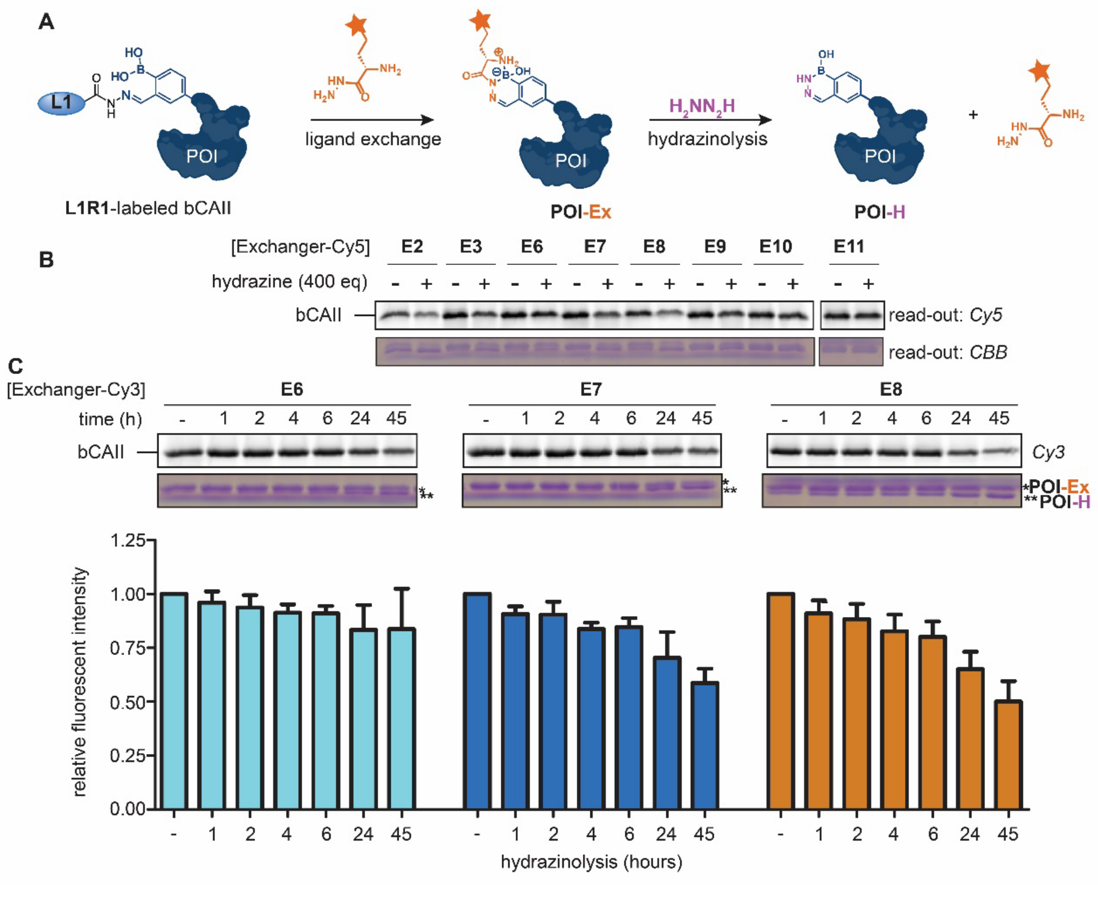

Figure S5. (A) Schematic representation of the stability test performed. (B) Effect of 400 equivalents of hydrazine fluorescence signal on **L1R1**-labeled bCAII after incubating for 24 hours. (C) Hydrazinolysis of the **E6-Cy3** (left, light blue bars), **E7-Cy3** (middle, blue bars) and **E8-Cy3** (right, orange bars) iminoboronate adducts followed over the course of 45 hours. * indicates the bCAII-fluorophore construct. ** indicates the hydrazinolysed product. Labeling conditions: **L1R1** (20 µM) was incubated with a mixture of bCAII (5 µM), avidin (25 µM), ovalbumin (25 µM) in HEPES

(50 mM, pH 7.4) for 2 hours. Exchange: SDS denatured samples were incubated with exchanger for 2 hours. Hydrazinolysis: 400 equivalents of hydrazine, pH 5.2, indicated amount of time.

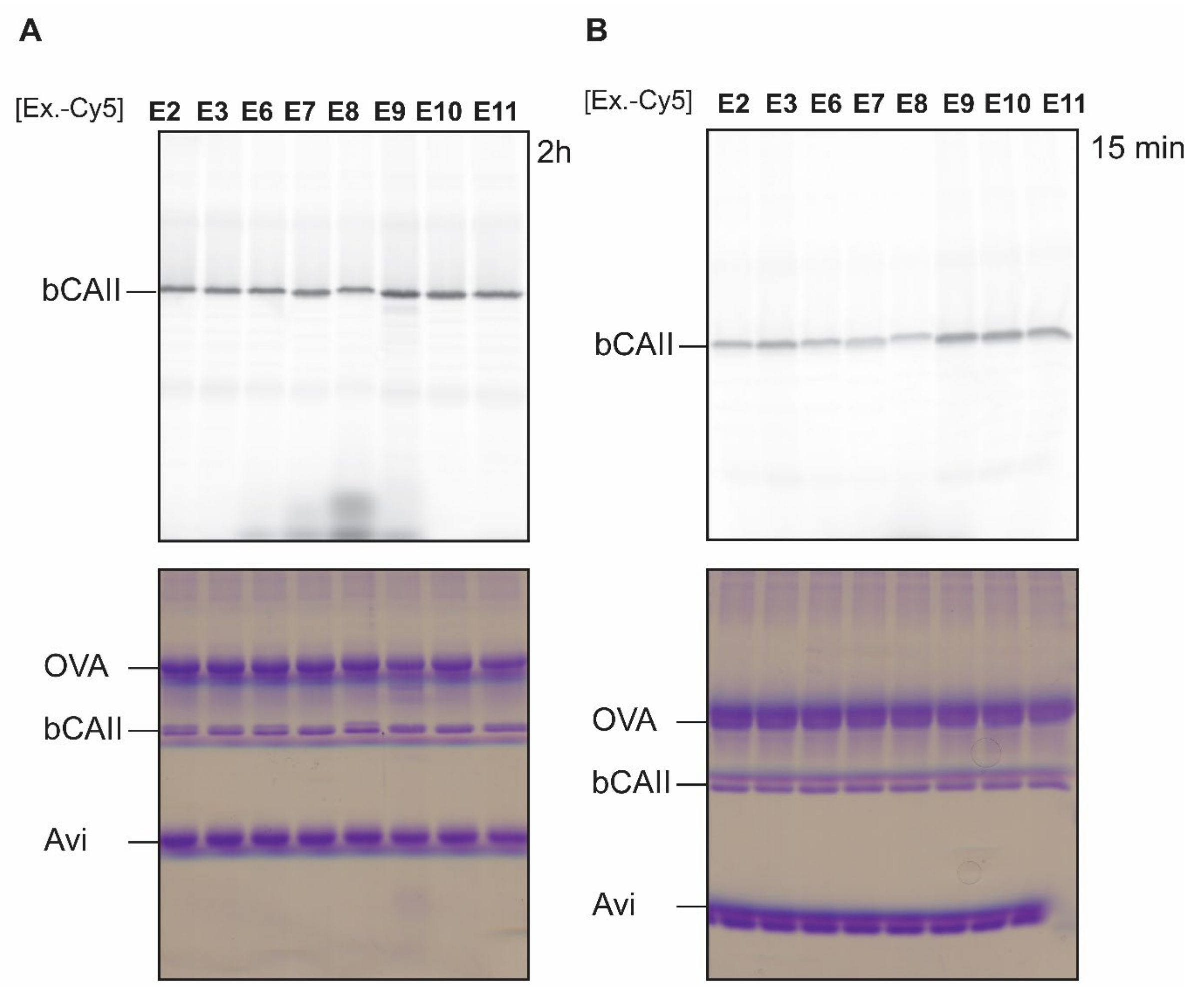

Figure S6. Uncropped images of the in-gel fluorescence scans depicted in panels A and B of Figure 5.

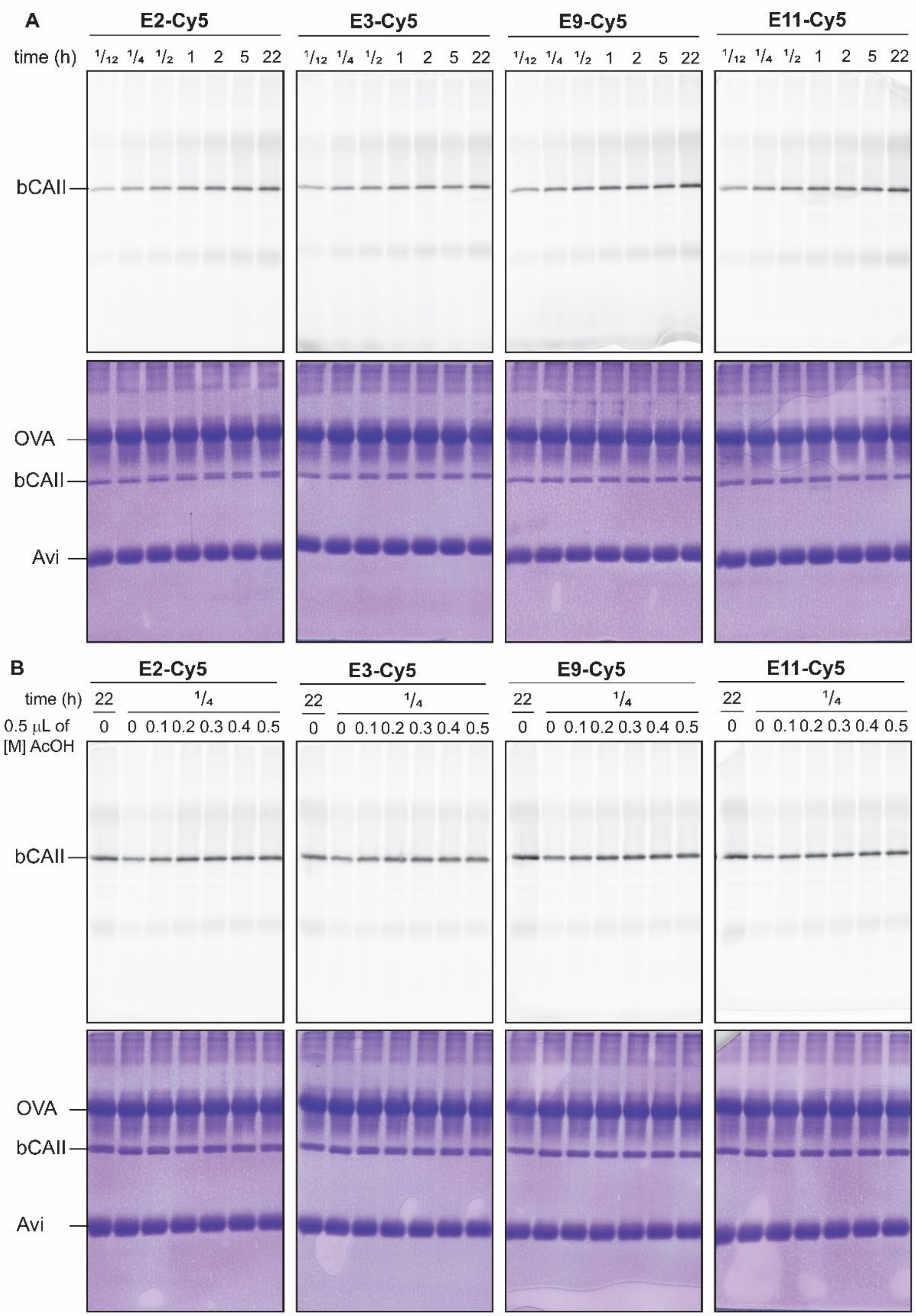
A
E2-Cy5
E3-Cy5
E9-Cy5
E11-Cy5
time (h)
1/12 1/4 1/2 1 2 5 22
bCAII
OVA
bCAII
Avi
B
E2-Cy5
E3-Cy5
E9-Cy5
E11-Cy5
time (h)
22
1/4
0.5 µL of [M] AcOH
0 0 0.1 0.2 0.3 0.4 0.5
bCAII
OVA
bCAII
Avi

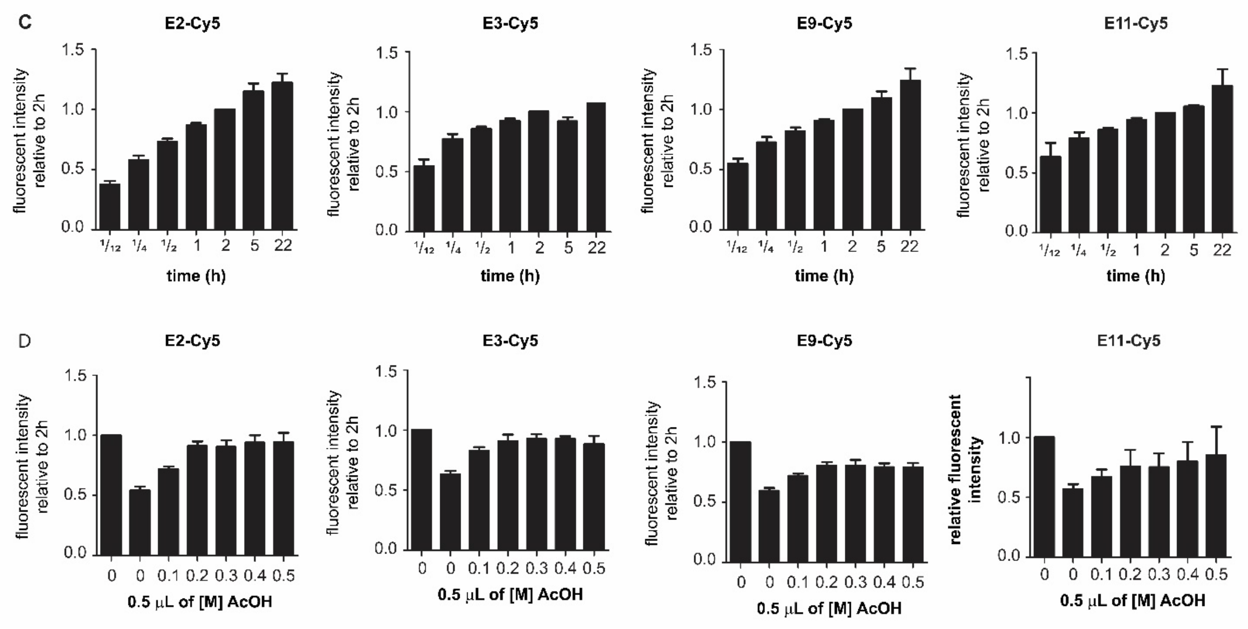

Figure S7. In gel fluorescence scan demonstrate the **e**ffect of the exchange time (A) and the pH (B) on the exchange efficiency of **E2**, **E3**, **E9** and **E11**. (C) Relative fluorescence of the exchange reaction over time. Normalized for the fluorescent signal obtained after 2 hours. The experiments were carried out in quadruplicate. (D) Influence of lowering the pH via addition of increasing concentrations acetic acid (AcOH) on an exchange reaction of 15 minutes. Normalized for the fluorescent signal obtained for an exchange reaction of 2 hours in the absence of acetic acid. The experiments were carried out in quadruplicate. Labeling conditions: **L1R1** (20 μM) was incubated with a mixture of bCAII (5 μM), avidin (25 μM), ovalbumin (25 μM) in HEPES (50 mM, pH 7.4) for 2 hours. (For uncropped gels and results with **E2**, **E3** and **E9** see Figure S6-S7)

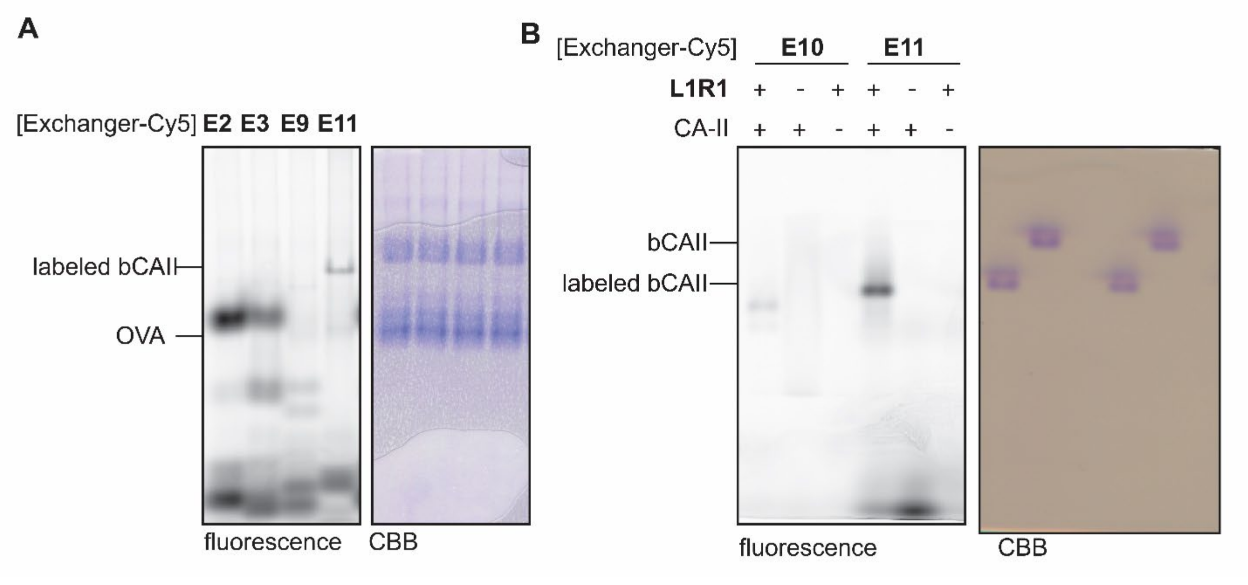

Figure S8. Uncropped images of the native exchange experiment depicted in Figure 6.

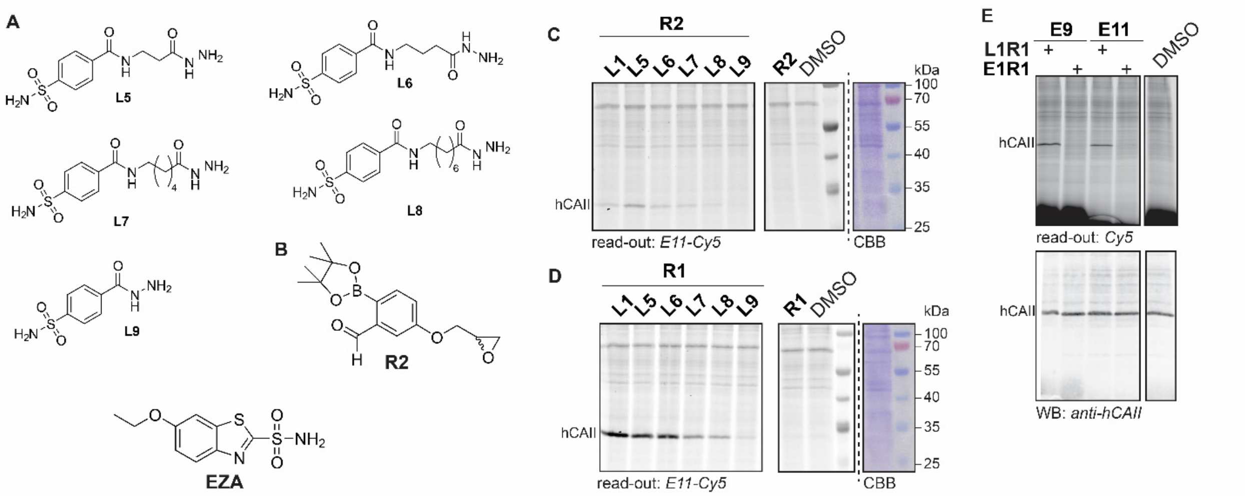

Figure S9. (A) Structures of **L5-L9**; (B) Structure of **R2** and **EZA**; (C) Labeling with **R2**-derived probes in HEK293 lysate. HEK293 cell lysate (2 mg/mL) in HEPES (50 mM, pH 7.4) was incubated with **R2**-based iminoboronate probes (1 μM) for 2 hours. Read-out with E11-Cy5 (1 μM). (D) Labeling with **R1**-derived probes in HEK293 lysate. HEK293 cell lysate (2 mg/mL) in HEPES (50 mM, pH 7.4) was incubated with **R1**-based iminoboronate probes (1 μM) for 2 hours. Read-out with E11-Cy5 (1 μM). (E)

Experimental procedures

General synthetic procedures

The synthesis of β-hydroxy hydrazide **E8** is described.[2] The synthesis of sulfonamide ligand **L1**, **L5-L9**, sulfonyl fluoride **R1**, epoxide **R2**, **FITC am-zide** are described.[3] Hydrazide **E1**,[4] 4-sulfamoylbenzyl *N*-hydroxysuccinimide ester **1**,[5] 1-(*tert*-butyl) 2-(4-nitrophenyl) hydrazine-1,2-dicarboxylate,[6] 5-(2-azidoethyl)-2,2-dimethyl-1,3-dioxolan-4-one **21**[7], methyl 4-(3-azidopropoxy)-2-hydroxybenzoate **26**[8] and *p*-toluenesulfonic acid 3-azidopropyl ester[9] were synthesized according to literature procedures. ^{1}H and ^{13}C NMR were recorded on a Varian AMX400 spectrometer or on a Bruker Avance NEO 600, using chloroform-*d*, DMSO-d_6, deuterium oxide or methanol-d_4 as solvent. Chemical shift values are reported in ppm with the solvent resonance as the internal standard (chloroform-*d*: δ 7.26 for ^{1}H, δ 77.16 for ^{13}C; methanol-d_4: δ 3.31 for ^{1}H, δ 49.00 for ^{13}C; DMSO-d_6: δ2.50 for ^{1}H δ 39.52 for ^{13}C; D_2O: δ 4.79 for ^{1}H). Data are reported as follows: chemical shifts (δ), multiplicity (s = singlet, d = doublet, dd = double doublet, ddd = double double doublet, t = triplet, q = quartet, p = quintet, m = multiplet), coupling constants J (Hz), and integration. High resolution mass measurements were performed using a ThermoScientific LTQ OrbitrapXL spectrometer using methanol as eluent.

NMR studies

TSP-d_4 (50 μL of 10 mM in D_2O) was added to PBS (390 μL of 50 mM, pH 7.4). To this was added 2-FPBA (5 μL of 100 mM in DMSO-d_6), followed by addition of **E1** or **E2** (5 μL of 100 mM in DMSO-d_6). The samples were incubated overnight and then once measured by ^{1}H NMR (water suppression protocol). Subsequently, hydrazine (10 μL of 2 M in D_2O) was added and the hydrazinolysis was followed over time by ^{1}H NMR (water suppression protocol).

Synthesis of sulfonamide-derived alkoxyamine L2

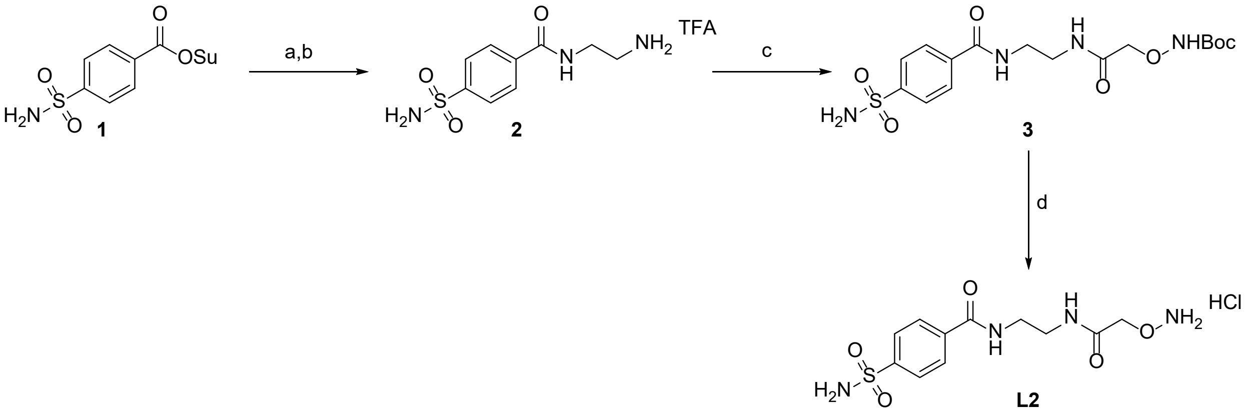

Scheme S1. Synthesis of **L2**. Reagents and conditions: a) Boc-ethylenediamine, DiPEA, DMF; b) TFA (quantitative over two steps); c) (Boc-aminooxy)acetic acid, HCTU, DiPEA, DMF (46%); d) HCl, 1,4-dioxane (95%).

***N*-(2-aminoethyl)-4-sulfamoylbenzamide TFA salt (2)**

To a solution of 4-sulfamoylbenzyl *N*-hydroxysuccinimide ester **1** (500 mg, 1.7 mmol) and Boc-ethylenediamine (323 mg, 2.0 mmol) in DMF (3.4 mL) was added DiPEA (0.75 mL, 4.3 mmol). After stirring at room temperature for 90 minutes, the mixture was concentrated under reduced pressure. The resulting white solid was dissolved in water (20 mL) and EtOAc (20 mL). The layers were separated and the water layer was extracted with EtOAc (20 mL, 1×). The combined organic layers were washed with brine (30 mL, 1×). The separatory funnel was rinsed with methanol, the methanol rinse was added combined with the organic layer, then the combined organic layers were dried over Na_2SO_4, filtered over cotton and concentrated under reduced pressure. The intermediate was dissolved in TFA (2 mL), stirred at room temperature for 2.5 hours and concentrated under reduced pressure to yield *N*-(2-aminoethyl)-4-sulfamoylbenzamide TFA salt **2** as an off-white solid (329 mg, 0.92 mmol, quantitative over two steps). ^1H NMR (400 MHz, methanol-d_4) δ 8.01 (app t, J = 8.1 Hz, 4H), 3.69 (t, J = 5.9 Hz, 2H), 3.19 (d, J = 5.9 Hz, 2H).

***tert*-butyl (2-oxo-2-((2-(4-sulfamoylbenzamido)ethyl)amino)ethoxy)carbamate (3)**

To a solution of **2** (30 mg, 0.08 mmol) in DMF (0.6 mL) was added HCTU (70 mg, 0.17 mmol), (Boc-aminooxy)acetic acid (34 mg, 0.18 mmol) and DiPEA (0.1 mL, 0.57 mmol) and the orange mixture was stirred overnight at room temperature. The mixture was concentrated under reduced pressure, dissolved in EtOAc (30 mL), washed with $KHSO_4$ (1M, 30 mL, 2×) and aqueous $NaHCO_3$ (saturated, 30 mL, 3×), dried over Na_2SO_4, filtered over cotton and concentrated under reduced pressure. After purification by column chromatography (1→2% methanol/dichloromethane), tert-butyl (2-oxo-2-((2-(4-sulfamoylbenzamido)ethyl)amino)ethoxy)carbamate **3** (16 mg, 0.038 mmol, 46%) was obtained as an off-white solid. ^1H NMR (400 MHz, methanol-d_4) δ 7.97 (s, 4H), 4.27 (s, 2H), 3.55 (t, 5.3 Hz, 2H), 3.53 (t, 5.3 Hz, 2H), 1.42 (s, 9H). ^{13}C NMR (101 MHz, methanol-d_4) δ 172.0, 169.2, 159.7, 147.7, 139.0, 129.1, 127.3, 83.1, 76.6, 49.0, 40.9, 39.6, 28.4. HRMS (ESI-orbitrap) m/z calculated for $[M+Na]^+$ = 439.126, found 439.126.

***N*-(2-(2-(aminooxy)acetamido)ethyl)-4-sulfamoylbenzamide hydrochloride (L2)**

HCl in 1,4-dioxane (4 M, 0.3 mL, 1.2 mmol) was added to **3** (10 mg, 0.024 mmol). After stirring for 30 minutes at room temperature, the mixture was centrifugated for 10 seconds and the supernatant was removed to yield *N*-(2-(2-(aminooxy)acetamido)ethyl)-4-sulfamoylbenzamide hydrochloride **L2** (8 mg, 0.023 mmol, 95%) as an off-white solid. ^1H NMR (400 MHz, methanol-d_4) δ 7.98 (s, 4H), 4.55 (s, 2H), 3.57

(t, J = 5.3 Hz, 2H), 3.50 (t, J = 5.3 Hz, 2H). ^{13}C NMR (101 MHz, methanol-d_4) δ 169.8, 169.3, 147.8, 138.8, 129.1, 127.3, 72.4, 40.6, 40.1.

Synthesis of sulfonamide-derived semicarbazide L3

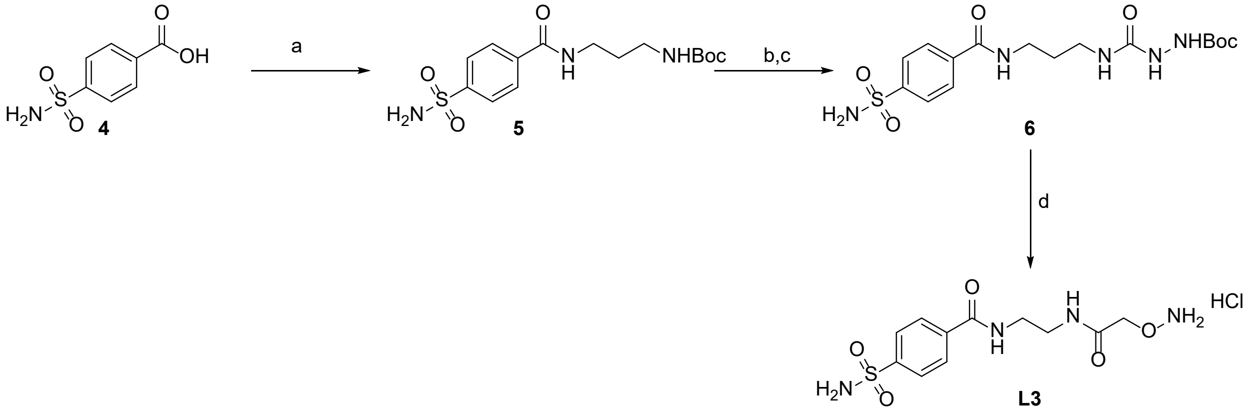

Scheme S2. Synthesis of **L3**. Reagents and conditions: a) Boc-propylenediamine, EDC.HCl, HOBt hydrate, DiPEA, DMF (66%); b) HCl, 1,4-dioxane, methanol; c) 1-(tert-butyl) 2-(4-nitrophenyl) hydrazine-1,2-dicarboxylate, DiPEA, DMF (26% over two steps); d) HCl, methanol (quantitative).

***tert*-butyl (3-(4-sulfamoylbenzamido)propyl)carbamate (5)**

N-(3-Dimethylaminopropyl)-*N'*-ethylcarbodiimide hydrochloride (EDC.HCl, 115 mg, 0.60 mmol) and HOBt hydrate (114 mg, 0.74 mmol) were added to a solution of 4-sulfamoylbenzoic acid **4** (114 mg, 0.57 mmol) in DMF (4 mL). Subsequently, a solution of Boc-propylenediamine (100 mg, 0.57 mmol) in DMF (1 mL) and DiPEA (0.3 mL, 1.7 mmol) were added, and the reaction mixture was stirred at room temperature for 1 hour. The mixture was concentrated under reduced pressure, dissolved in EtOAc (50 mL), washed with aqueous $KHSO_4$ (1M, 40 mL, 2×), $NaHCO_3$ (saturated, 40 mL, 3×) and brine (40 mL, 1×), dried over Na_2SO_4, filtered over cotton and concentrated under reduced pressure. The crude was triturated with dichloromethane (40 mL) to yield *tert*-butyl (3-(4-sulfamoylbenzamido)propyl)carbamate **5** (134 mg, 0.38 mmol, 66%) as a white solid. ^1H NMR (400 MHz, DMSO-d_6) δ 8.60 (t, J = 5.7 Hz, 1H), 7.97 (d, J = 8.5 Hz, 2H), 7.89 (d, J = 8.5 Hz, 2H), 6.81 (t, J = 5.8 Hz, 1H), 3.26 (q, J = 6.7 Hz, 2H), 2.98 (q, J = 6.6 Hz, 2H), 1.64 (m, 2H), 1.37 (s, 9H). ^{13}C NMR (101 MHz, DMSO-d_6) δ 165.1, 155.6, 146.2, 137.5, 127.8, 125.6, 77.5, 37.7, 37.1, 29.5, 28.3.

***tert*-butyl 2-((3-(4-sulfamoylbenzamido)propyl)carbamoyl)hydrazine-1-carboxylate (6)**

HCl in 1,4-dioxane (4M, 0.25 mL, 1 mmol) was added to a solution of **5** (54 mg, 0.15 mmol) in methanol (3 mL) and the mixture was stirred at room temperature for 3 hours. The crude was concentrated under reduced pressure and dissolved in DMF (3 mL). Subsequently, DiPEA (0.15 mL) and 1-(*tert*-butyl) 2-(4-nitrophenyl) hydrazine-1,2-dicarboxylate (57 mg, 0.19 mmol) were added and the solution was stirred at room temperature overnight. The mixture was concentrated under reduced pressure, diluted with EtOAc (50 mL), washed with aqueous $KHSO_4$ (1M, 30 mL, 2×), $NaHCO_3$ (saturated, 30 mL, 3×) and brine (30 mL, 1×), dried over Na_2SO_4, filtered over cotton and concentrated under reduced pressure. Purification by column chromatography (dry-loaded on celite, 10% methanol/dichloromethane) yielded *tert*-butyl 2-((3-(4-sulfamoylbenzamido)propyl)carbamoyl)hydrazine-1-carboxylate **7** (16 mg, 0.039 mmol, 26%) as a yellow solid. ^1H NMR (400 MHz, methanol-d_4) δ 7.98 (s, 4H), 3.45 (t, J = 6.6 Hz, 2H), 3.26 (t, J = 6.6 Hz, 2H), 1.77 (m, 2H), 1.47 (s, 9H). ^{13}C NMR (101 MHz, methanol-d_4) δ 168.8, 161.8, 158.4, 147.7, 139.0, 129.0, 127.3, 82.0, 38.0, 37.9, 30.9, 28.6.

N-(3-(4-sulfamoylbenzamido)propyl)hydrazinecarboxamide hydrochloride (L3)

Methanolic HCl (3M, 0.38 mL, 1.1 mmol) was added to a solution of **6** (16 mg, 0.39 mmol) in methanol (2 mL) and the mixture was stirred at room temperature for 5 hours. The mixture was concentrated under reduced pressure and co-evaporated with methanol (2×) to yield *N*-(3-(4-sulfamoylbenzamido)propyl)hydrazinecarboxamide hydrochloride **L3** (14 mg, 0.039 mmol, quantitative) as a yellow solid. ^{1}H NMR (400 MHz, Methanol-d_4) δ 7.94 (s, 4H), 3.42 (t, J = 6.8 Hz, 2H), 3.29 – 3.22 (m, 2H), 1.79 (m, 2H). ^{13}C NMR (101 MHz, Methanol-d_4) δ 168.9, 159.1, 147.7, 138.9, 129.0, 127.3, 38.5, 38.2, 30.7.

Synthesis of sulfonamide-derived amine L4

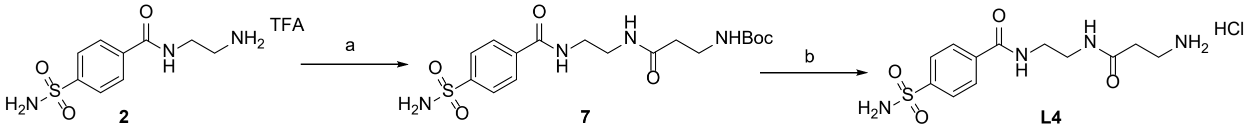

Scheme S3. Synthesis of **L4**. Reagents and conditions a) Boc-β-alanine, HCTU, DiPEA, DMF (9%); b) acetyl chloride, methanol (quantitative)

***tert*-butyl (3-oxo-3-((2-(4-sulfamoylbenzamido)ethyl)amino)propyl)carbamate (7)**

2 (83 mg, 0.23 mmol), Boc-β-alanine (40 mg, 0.21 mmol), HCTU (88 mg, 0.21 mmol) were dissolved in DMF (1.1 mL), then DiPEA (0.1 mL, 0.57 mmol) was added and the mixture was stirred for 1 hour at room temperature. The mixture was concentrated under reduced pressure, dissolved in EtOAc (20 mL), washed with $KHSO_4$ (1M, 30 mL, 2×) and aqueous $NaHCO_3$ (saturated, 30 mL, 3×), dried over Na_2SO_4, filtered over cotton and concentrated under reduced pressure. Purification by reverse phase column chromatography (5 → 95% acetonitrile/water) yielded *tert*-butyl (3-oxo-3-((2-(4-sulfamoylbenzamido)ethyl)amino)propyl)carbamate **7** (8 mg, 0.02 mmol, 9%) as a white solid. ^{1}H NMR (400 MHz, methanol-d_4) δ 7.98 (s, 4H), 3.51 (t, *J* = 5.6 Hz, 2H), 3.41 (t, *J* = 5.9 Hz, 2H), 3.31 (t, 2H), 2.36 (t, *J* = 6.7 Hz, 2H), 1.41 (s, 9H). ^{13}C NMR (101 MHz, methanol-d_4) δ 174.6, 169.0, 147.7, 138.9, 130.5, 129.0, 127.3, 80.2, 41.0, 40.0, 38.1, 37.6, 28.7. HRMS (ESI-orbitrap) *m/z* calculated for $[M+Na]^+$ = 437.147, found 437.147.

***N*-(2-(3-aminopropanamido)ethyl)-4-sulfamoylbenzamide hydrochloride (L4)**

Methanol (1 mL, 25.70 mmol) was cooled in an ice-water bath. followed by dropwise addition of acetyl chloride (5.3 mL, 74.09 mmol). Subsequently, part of the pre-made HCl solution (2 mL) was added dropwise to **7** (8 mg, 0.02 mmol). After stirring for 20 minutes at room temperature, the mixture was concentrated under reduced pressure and triturated with pentane (2 mL) to yield *N*-(2-(3-aminopropanamido)ethyl)-4-sulfamoylbenzamide hydrochloride **L4** (7 mg, 0.02 mmol, quantitative) as a white solid. ^{1}H NMR (400 MHz, methanol-d_4) δ 7.98 (d, J = 0.9 Hz, 4H), 3.55 (t, J = 5.9 Hz, 2H), 3.43 (t, J = 5.9 Hz, 2H), 3.18 (t, J = 6.5 Hz, 2H), 2.60 (t, J = 6.4 Hz, 2H). ^{13}C NMR (101 MHz, methanol-d_4) δ 172.6, 169.2, 147.8, 138.9, 129.1, 127.3, 40.6, 40.3, 37.2, 32.9. HRMS (ESI-orbitrap) m/z calculated for $[M+Na]^+$ = 315.112, found 315.112.

Synthesis of alkoxyamine E2

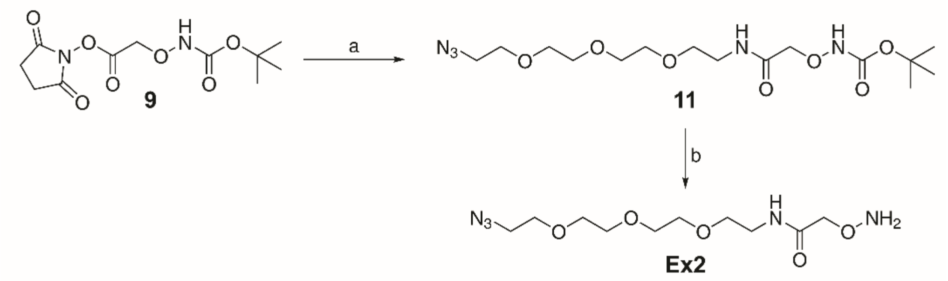

Scheme S4. Synthesis of **E2**. Reagents and conditions: a) 2-(2-(2-(2-azidoethoxy)ethoxy)ethoxy)ethan-1-amine, DiPEA, dichloromethane (74%); b) HCl, methanol (77%).

***tert*-butyl ((14-azido-2-oxo-6,9,12-trioxa-3-azatetradecyl)oxy)carbamate (11)**

(Boc-aminooxy)acetic acid *N*-hydroxysuccinimide ester **9** (334 mg, 1.2 mmol) was added to a solution of 2-(2-(2-(2-azidoethoxy)ethoxy)ethoxy)ethan-1-amine (106 mg, 0.49 mmol) in dichloromethane (5 mL). Subsequently, DiPEA (0.36 mL, 2.1 mmol) was added and the reaction mixture was stirred at room temperature for 4 h. The solvent was removed under reduced pressure. The crude was dissolved in EtOAc (80 mL), washed with aqueous $KHSO_4$ (1 M, 70 mL, 2×), $NaHCO_3$ (saturated, 70 mL, 3×) and brine (70 mL, 1×), dried over Na_2SO_4, filtered over cotton and concentrated under reduced pressure. Purification by column chromatography (2% methanol/dichloromethane) yielded *tert*-butyl ((14-azido-2-oxo-6,9,12-trioxa-3-azatetradecyl)oxy)carbamate **11** (141 mg, 0.36 mmol, 74%) as a colorless oil. ^{1}H NMR (400 MHz, Chloroform-*d*) δ 7.88 (s, 1H), 7.82 (s, 1H), 4.35 – 4.30 (m, 2H), 3.67 (apparent s, 10H), 3.59 (m, 2H), 3.55 – 3.46 (m, 2H), 3.38 (m, 2H), 1.48 (s, 9H). ^{13}C NMR (101 MHz, Chloroform-*d*) δ 169.0, 157.5, 82.8, 76.1, 70.8, 70.7, 70.7, 70.4, 70.1, 69.6, 50.8, 39.0, 28.3. HRMS (ESI-orbitrap) m/z calculated for $[M+H]^+$ 392.2140, found 392.2131; m/z calculated for $[M+H\text{-}Boc]^+$ 292.1616, found 292.1614.

2-(aminooxy)-*N*-(2-(2-(2-(2-azidoethoxy)ethoxy)ethoxy)ethyl)acetamide (E2)

Methanolic HCl (3 M, 1 mL, 3.1 mmol) was added to a solution of *tert*-butyl ((14-azido-2-oxo-6,9,12-trioxa-3-azatetradecyl)oxy)carbamate **11** (122 mg, 0.31 mmol) in methanol (3 mL) and the reaction mixture was stirred at room temperature overnight. The mixture was concentrated under reduced pressure, co-evaporated with methanol (2×) and purified by column chromatography (1/2/97 Et_3N/methanol/dichloromethane) to yield 2-(aminooxy)-*N*-(2-(2-(2-(2-azidoethoxy)ethoxy)ethoxy)ethyl)acetamide **E2** (70 mg, 0.24 mmol, 77%) as a colorless oil. ^{1}H NMR (400 MHz, Methanol-*d*$_4$) δ 4.10 (d, J = 1.8 Hz, 2H), 3.68 – 3.62 (m, 10H), 3.58 (m, 2H), 3.45 (m, 2H), 3.40 – 3.36 (m, 2H). ^{13}C NMR (101 MHz, Methanol-*d*$_4$) δ 172.9, 75.4, 71.6, 71.5, 71.5, 71.2, 71.0, 70.4, 51.7, 39.8. HRMS (ESI-orbitrap) m/z calculated for $[M+H]^+$ 292.1616, found 292.1615.

Synthesis of semicarbazide E3

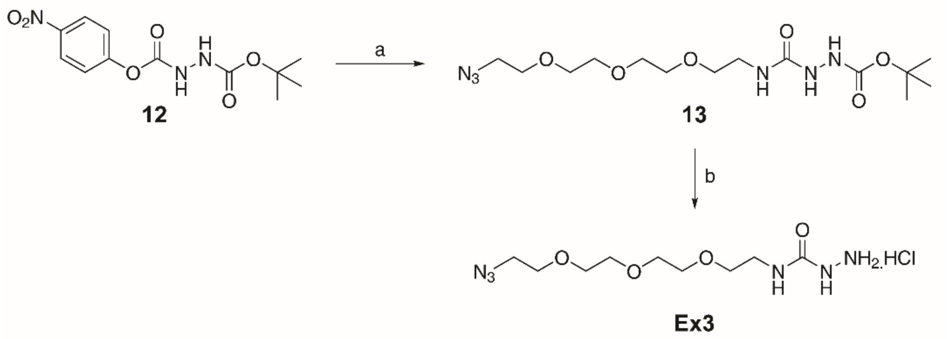

Scheme S5. Synthesis of **E3**. Reagents and conditions: a) 2-(2-(2-(2-azidoethoxy)ethoxy)ethoxy)ethan-1-amine, DiPEA, dichloromethane (91%); HCl, methanol (86%).

***tert*-butyl 16-azido-4-oxo-8,11,14-trioxa-2,3,5-triazahexadecanoate (13)**

1-(*tert*-butyl) 2-(4-nitrophenyl) hydrazine-1,2-dicarboxylate **12** (73 mg, 0.25 mmol) and DiPEA (0.21 mL, 1.2 mmol) were added to a solution of 2-(2-(2-(2-azidoethoxy)ethoxy)ethoxy)ethan-1-amine (75 mg, 0.34 mmol) in dichloromethane (3 mL) and the reaction mixture was stirred at room temperature overnight. The mixture was diluted with dichloromethane (50 mL), washed with aqueous $KHSO_4$ (1 M, 50 mL, 1×), $NaHCO_3$ (saturated, 50 mL, 2×) and brine (50 mL, 1×), dried over Na_2SO_4, filtered over cotton and concentrated under reduced pressure. Purification by column chromatography (1→4% methanol/dichloromethane) yielded *tert*-butyl 16-azido-4-oxo-8,11,14-trioxa-2,3,5-triazahexadecanoate **13** (84 mg, 0.22 mmol, 91%) as a colorless oil. ^{1}H NMR (400 MHz, Chloroform-*d*) δ 6.48 (s, 1H), 6.28 (s, 1H), 5.85 – 5.76 (m, 1H), 3.72 – 3.66 (m, 6H), 3.64 (s, 4H), 3.57 (m, 2H), 3.43 (m, 4H), 1.47 (s, 9H). ^{13}C NMR (101 MHz, Chloroform-*d*) δ 158.6, 155.9, 81.9, 70.9, 70.7, 70.6, 70.4, 70.4, 70.1, 50.8, 40.2, 28.3. HRMS (ESI-orbitrap) m/z calculated for $[M+H]^+$ 399.1963, found 399.1960.

***N*-(2-(2-(2-(2-azidoethoxy)ethoxy)ethoxy)ethyl)hydrazinecarboxamide hydrochloride (E3)**

Methanolic HCl (3 M, 1.4 mL, 4.1 mmol) was added to a solution of **13** (77 mg, 0.21 mmol) in methanol (3 mL) and the reaction was stirred at room temperature overnight. The mixture was concentrated under reduced pressure and co-evaporated with methanol (3×) to yield *N*-(2-(2-(2-(2-azidoethoxy)ethoxy)ethoxy)ethyl)hydrazinecarboxamide hydrochloride **E3** (55 mg, 0.18 mmol, 86%) as a yellowish oil. ^{1}H NMR (400 MHz, Methanol-*d₄*) δ 3.71 – 3.61 (m, 10H), 3.57 (m, 2H), 3.38 (m, 4H). ^{13}C NMR (101 MHz, Methanol-d_4) δ 159.0, 71.5, 71.5, 71.4, 71.3, 71.0, 51.7, 41.1. HRMS (ESI-orbitrap) m/z calculated for $[M+H]^+$ 299.1438, found 299.1439.

Synthesis of amine E4

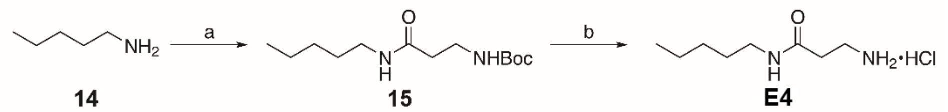

Scheme S6. Synthesis of **E4**. Reagents and conditions: a) Boc-β-alanine, HCTU, DiPEA, DMF; b) HCl, 1,4-dioxane (89%)

3-amino-*N*-pentylpropanamide hydrochloride (E4)

To a solution of pentylamine **14** (66 µL, 0.50 mg, 0.57 mmol) in DMF (3 mL) was added Boc-β-alanine (217 mg, 1.15 mmol), HCTU (475 mg, 1.15 mmol) and DiPEA (400 µL, 2.30 mmol). The mixture was stirred at room temperature for 1 hour, after which TLC indicated complete conversion. The mixture was diluted with CH_2Cl_2 (20 mL) and washed twice with aqueous $KHSO_4$ (1M, 20 mL), thrice with sat. aqueous sodium bicarbonate solution (20 mL). The organic layer was collected and dried over Na_2SO_4. NMR analysis of the

crude product indicated that tert-butyl (3-(butylamino)-3-oxopropyl)carbamate **15** had formed. The crude product (51 mg) was immediately subjected to the deprotection step.

HCl in 1,4-dioxane (4 M, 1 mL, 4 mmol) was added dropwise to **15** (51 mg, 0.20 mmol) and stirred at room temperature for 10 minutes. The reaction mixture was transferred to Eppendorf tubes, centrifuged for 10 seconds and the supernatant was removed. Subsequently, the white solid was dissolved in methanol and the combined fractions were concentrated under reduced pressure to yield 3-amino-*N*-pentylpropanamide hydrochloride **E4** (34 mg, 0.18 mmol, 31% over two steps) as a white solid. ^{1}H NMR (400 MHz, methanol-d_4) δ 3.18 (m, 4H), 2.64 (t, *J* = 6.5 Hz, 2H), 1.52 (p, *J* = 7.1 Hz, 2H), 1.41 – 1.26 (m, 4H), 0.91 (t, *J* = 6.8 Hz, 3H). ^{13}C NMR (101 MHz, methanol-d_4) δ 172.0, 40.4, 37.2, 32.8, 30.2, 29.9, 23.4, 14.3. HRMS (ESI-orbitrap) *m/z* calculated for $[M+H]^+$ = 159.149, found 159.149.

Synthesis of sulfonyl hydrazide E5

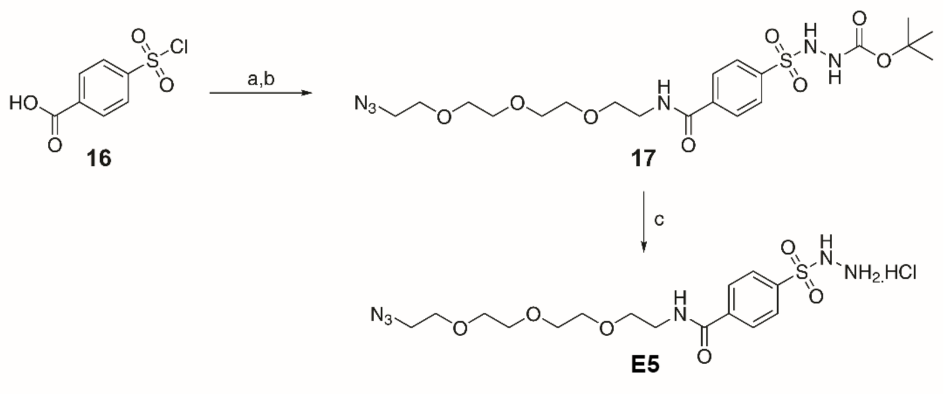

Scheme S7. Synthesis of **E5**. Reagents and conditions: a) tert-butyl carbazate, THF, 0 °C to rt; b) EDC.HCl, 2-(2-(2-(2-azidoethoxy)ethoxy)ethoxy)ethan-1-amine, DMF (22% over two steps); c) HCl, methanol (quantitative).

***tert*-Butyl 2-((4-((2-(2-(2-(2-azidoethoxy)ethoxy)ethoxy)ethyl)carbamoyl)phenyl)sulfonyl)hydrazine-1-carboxylate (17)**

A suspension of 4-(chlorosulfonyl)benzoic acid **16** (202 mg, 0.92 mmol) in THF (10 mL) was cooled with an ice-water bath, followed by addition of t*ert*-butyl carbazate (359 mg, 2.7 mmol). After 30 minutes of stirring, the cooling bath was removed and the mixture was left stirring at room temperature overnight. After removal of the solvent under reduced pressure, the crude was dissolved in EtOAc (90 mL) and aqueous $KHSO_4$ (1 M, 60 mL). The layers were separated and the organic layer was washed with aqueous $KHSO_4$ (1 M, 60 mL, 2×) and brine (60 mL, 1×), dried over Na_2SO_4, filtered over cotton and concentrated under reduced pressure.

Without any further purification, the crude was dissolved in DMF (10 mL). *N*-(3-Dimethylaminopropyl)-*N′*-ethylcarbodiimide hydrochloride (EDC.HCl; 208 mg, 1.1 mmol) was added, followed by addition of a solution of 2-(2-(2-(2-azidoethoxy)ethoxy)ethoxy)ethan-1-amine (235 mg, 1.1 mmol) in DMF (2 mL). After having stirred at room temperature overnight, the reaction mixture was concentrated under reduced pressure, diluted with EtOAc (80 mL), washed with aqueous $KHSO_4$ (1 M, 60 mL, 2×), $NaHCO_3$ (saturated, 60 mL, 2×) and brine (60 mL, 1×), dried over Na_2SO_4, filtered over cotton and concentrated under reduced pressure. Purification by column chromatography (2% methanol/dichloromethane) yielded *tert*-butyl 2-((4-((2-(2-(2-(2-azidoethoxy)ethoxy)ethoxy)ethyl)carbamoyl)phenyl)sulfonyl)hydrazine-1-carboxylate **17** (106 mg, 0.21 mmol, 22%) as a colorless thick oil. ^{1}H NMR (600 MHz, DMSO-d_6) δ 9.70 (s, 1H), 9.17 (s, 1H), 8.72 (t, J = 5.6 Hz, 2H), 7.99 (d, J = 8.1 Hz, 2H), 7.83 (d, J = 8.5 Hz, 2H), 3.59 – 3.57 (m, 2H), 3.55 – 3.52 (m,

8H), 3.43 (q, J = 5.9 Hz, 2H), 3.39 – 3.36 (m, 2H), 3.17 (d, J = 3.9 Hz, 2H), 1.22 (s, 9H). 13C NMR (151 MHz, DMSO-d6) δ 165.1, 127.6, 79.5, 69.8, 69.8, 69.7, 69.6, 69.2, 68.7, 50.0, 48.6, 27.7. HRMS (ESI-orbitrap) m/z calculated for $[M+H]^+$ 517.2075, found 517.2057.

***N*-(2-(2-(2-(2-azidoethoxy)ethoxy)ethoxy)ethyl)-4-(hydrazineylsulfonyl)benzamide hydrochloride (E5)**
Methanolic HCl (3 M, 0.7 mL, 2.1 mmol) was added to a solution of **17** (94 mg, 0.18 mmol) in methanol (3 ml) and the reaction mixture was stirred at room temperature overnight. The mixture was concentrated under reduced pressure and co-evaporated with methanol (1×) to yield *N*-(2-(2-(2-(2-azidoethoxy)ethoxy)ethoxy)ethyl)-4-(hydrazineylsulfonyl)benzamide hydrochloride **E5** (83 mg, 0.18 mmol, quantitative) as a colorless oil. ^1H NMR (400 MHz, Methanol-d_4) δ 8.02 (m, 4H), 3.72 – 3.56 (m, 14H), 3.38 – 3.32 (m, 2H). ^{13}C NMR (101 MHz, Methanol-d_4) δ 168.4, 141.0, 140.8, 129.6, 129.4, 71.6, 71.5, 71.4, 71.3, 71.0, 70.3, 51.7, 41.1. HRMS (ESI-orbitrap) m/z calculated for $[M+H]^+$ 417.1551, found 417.1548.

Synthesis of α-amino hydrazide E6

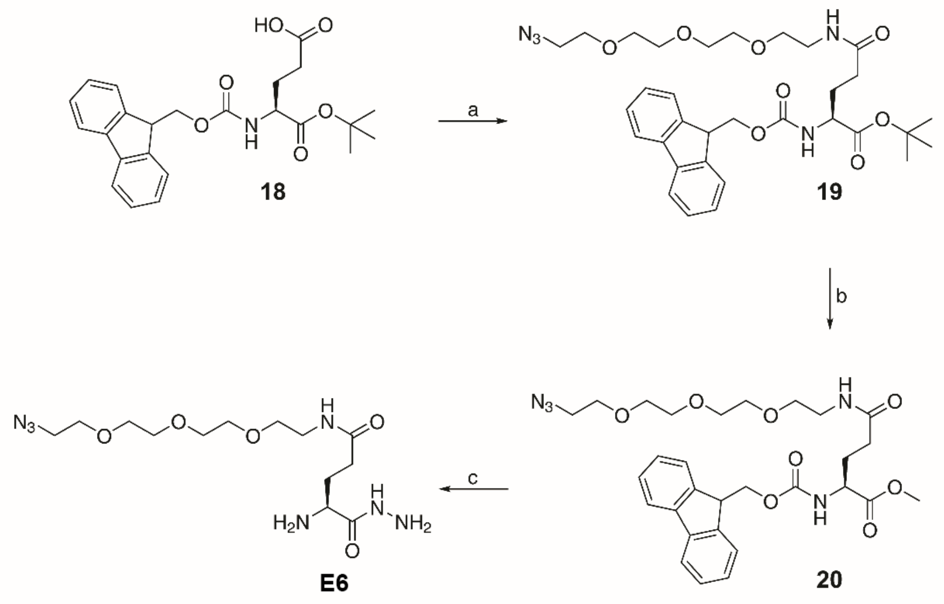

Scheme S8. Synthesis of **E6**. Reagents and conditions; a) 2-(2-(2-(2-azidoethoxy)ethoxy)ethoxy)ethan-1-amine, EDC.HCl, dichloromethane (79%); b) HCl, methanol (84%); c) hydrazine hydrate, methanol (53%).

***tert*-butyl (*S*)-16-((((9H-fluoren-9-yl)methoxy)carbonyl)amino)-1-azido-13-oxo-3,6,9-trioxa-12-azaheptadecan-17-oate (19)**
EDC.HCl (258 mg, 1.35 mmol) was added to a solution of Fmoc-Glu(OH)-OtBu **18** (521 mg, 1.22 mmol) in dichloromethane (6 mL). Subsequently, a solution of 2-(2-(2-(2-azidoethoxy)ethoxy)ethoxy)ethan-1-amine (286 mg, 1.31 mmol) in dichloromethane (4 mL) was added and the reaction mixture was stirred at room temperature for 1 h. The solvent was removed under reduced pressure. The crude was dissolved in EtOAc (80 mL), washed with aqueous $KHSO_4$ (1 M, 70 mL, 3×), $NaHCO_3$ (saturated, 70 mL, 3×) and brine (70 mL, 1×), dried over Na_2SO_4, filtered over cotton and concentrated under reduced pressure. Purification by column chromatography (dry-loaded on celite, 0.5→1.5% methanol/dichloromethane, then when the product would not elute 100% methanol) yielded *tert*-butyl (*S*)-16-((((9H-fluoren-9-yl)methoxy)carbonyl)amino)-1-azido-13-oxo-3,6,9-trioxa-12-azaheptadecan-17-oate **19** (609 mg, 0.97

mmol, 79%) as a yellow oil. ^{1}H NMR (400 MHz, Methanol-*d_4*) δ 7.80 (d, J = 7.5 Hz, 2H), 7.68 (t, J = 6.6 Hz, 2H), 7.39 (t, J = 7.4 Hz, 2H), 7.31 (t, J = 7.4 Hz, 2H), 4.45 – 4.28 (m, 2H), 4.22 (t, J = 6.8 Hz, 1H), 4.05 (m, 1H), 3.61 (m, 8H), 3.53 (t, J = 5.4 Hz, 2H), 3.35 (m, 4H), 2.36 – 2.27 (m, 2H), 2.17 – 2.08 (m, 1H), 1.96 – 1.82 (m, 1H), 1.46 (s, 9H). ^{13}C NMR (101 MHz, Methanol-*d_4*) δ 174.9, 172.9, 145.3, 145.2, 142.6, 128.8, 128.2, 126.3, 126.2, 120.9, 82.9, 71.6, 71.6, 71.5, 71.3, 71.1, 70.5, 68.0, 55.8, 51.8, 48.4, 40.4, 33.3, 28.6, 28.3. HRMS (ESI-orbitrap) m/z calculated for [M+H]$^+$ 648.3004, found 648.2981.

methyl (*S*)-16-((((9H-fluoren-9-yl)methoxy)carbonyl)amino)-1-azido-13-oxo-3,6,9-trioxa-12-azaheptadecan-17-oate (20)

Methanolic HCl (3 M, 3.4 mL, 10 mmol) was added to a solution of *tert*-butyl (S)-16-((((9H-fluoren-9-yl)methoxy)carbonyl)amino)-1-azido-13-oxo-3,6,9-trioxa-12-azaheptadecan-17-oate **19** (600 mg, 0.95 mmol) in methanol (9 mL) and the reaction mixture was stirred at room temperature overnight. The mixture was concentrated under reduced pressure and purified by column chromatography (dry-loaded on celite, 4% methanol/dichloromethane) to yield methyl (S)-16-((((9H-fluoren-9-yl)methoxy)carbonyl)amino)-1-azido-13-oxo-3,6,9-trioxa-12-azaheptadecan-17-oate **20** (469 mg, 0.80 mmol, 84%) as a yellow oil. ^{1}H NMR (400 MHz, Methanol-*d_4*) δ 7.79 (d, J = 7.5 Hz, 2H), 7.67 (t, J = 6.9 Hz, 2H), 7.39 (t, J = 7.4 Hz, 2H), 7.31 (t, J = 7.2 Hz, 2H), 4.44 – 4.29 (m, 2H), 4.25 – 4.17 (m, 2H), 3.72 (s, 3H), 3.62 (m, 8H), 3.52 (t, J = 5.4 Hz, 2H), 3.38 – 3.31 (m,42H), 2.41 (t, J = 7.3 Hz,0.5H), 2.29 (t, J = 7.4 Hz, 1.5H), 2.22 – 2.09 (m, 1H), 1.98 – 1.89 (m, 1H). ^{13}C NMR (101 MHz, Methanol-*d_4*) δ 174.6, 174.1, 145.3, 145.2, 142.6, 128.8, 128.2, 128.2, 126.3, 126.2, 120.9, 71.6, 71.6, 71.5, 71.2, 71.1, 70.5, 68.0, 55.0, 52.8, 52.2, 51.7, 48.4, 40.4, 33.1, 28.5. HRMS (ESI-orbitrap) m/z calculated for [M+H]$^+$ 584.2715, found 584.2697.

(*S*)-4-amino-*N*-(2-(2-(2-(2-azidoethoxy)ethoxy)ethoxy)ethyl)-5-hydrazineyl-5-oxopentanamide (E6)

Hydrazine hydrate (0.4 mL, 8.2 mmol) was added to a solution of methyl (*S*)-16-((((9H-fluoren-9-yl)methoxy)carbonyl)amino)-1-azido-13-oxo-3,6,9-trioxa-12-azaheptadecan-17-oate **20** (158 mg, 0.27 mmol) in methanol (3 mL) and the reaction mixture was stirred at room temperature overnight. The mixture was washed with heptane (4 mL, 3×), concentrated under reduced pressure and co-evaporated with methanol (2×). Purification by column chromatography (dry-loaded on celite, 4→20% methanol/dichloromethane) yielded (*S*)-4-amino-N-(2-(2-(2-(2-azidoethoxy)ethoxy)ethoxy)ethyl)-5-hydrazineyl-5-oxopentanamide **E6** (51 mg, 0.14 mmol, 53%) as a colorless oil. ^{1}H NMR (400 MHz, Methanol-*d_4*) δ 3.68 – 3.61 (m, 10H), 3.55 (t, J = 4.9 Hz, 2H), 3.40 – 3.34 (m, 4H), 3.26 (t, J = 6.8 Hz, 1H), 2.27 (m, 2H), 2.00 – 1.76 (m, 2H). ^{13}C NMR (101 MHz, Methanol-*d_4*) δ 176.1, 175.3, 71.6, 71.6, 71.5, 71.3, 71.1, 70.6, 54.3, 51.8, 40.4, 33.2, 32.4. HRMS (ESI-orbitrap) m/z calculated for [M+H]$^+$ 362.2146, found 362.2147.

Synthesis of α-hydroxy hydrazide E7

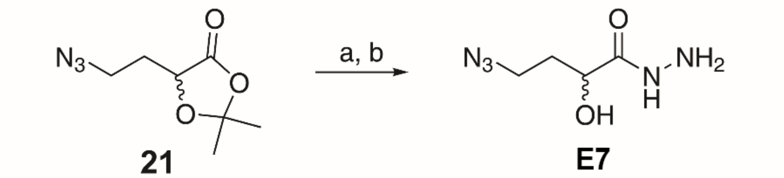

21 E7

Scheme S9. Synthesis of **E7**. Reagents and conditions: a) p-TsOH, methanol; b) hydrazine hydrate, methanol (37% over two steps).

4-azido-2-hydroxybutanehydrazide (E7)

p-toluenesulfonic acid monohydrate (20 mg, 0.11 mmol) was added to a solution of 5-(2-azidoethyl)-2,2-dimethyl-1,3-dioxolan-4-one **21**[7] (47 mg, 0.25 mmol) in methanol (10 mL) and the reaction mixture was refluxed for 2 h. The mixture was diluted with EtOAc (20 mL), washed with $NaHCO_3$ (saturated, 15 mL, 2×) and brine (15 mL, 1×), dried over Na_2SO_4, filtered and concentrated under reduced pressure to yield the crude methyl ester (22 mg).

Without further purification, the crude was dissolved in methanol (5 mL), followed by addition of hydrazine hydrate (0.2 mL, 4.1 mmol). The reaction mixture was stirred at room temperature overnight. The mixture was concentrated under reduced pressure and purified by column chromatography (dry-loaded on celite, 6% methanol/dichloromethane) to yield 4-azido-2-hydroxybutanehydrazide **E7** (15 mg, 0.094 mmol, 37% yield over two steps) as a yellow oil. ^1H NMR (400 MHz, DMSO-d_6) δ 8.93 (s, 1H), 5.53 (s, 1H), 4.20 (s, 2H), 3.95 (dd, J = 8.6, 4.0 Hz, 1H), 3.39 (dd, J = 7.7, 6.1 Hz, 2H), 1.92 – 1.83 (m, 1H), 1.74 – 1.63 (m, 1H). ^{13}C NMR (101 MHz, DMSO-d_6) δ 172.0, 67.8, 47.2, 33.4. HRMS (ESI-orbitrap) m/z calculated for $[M+Na]^+$ 182.0649, found 182.0645.

Synthesis of *N*-Hydroxysemicarbazide E9

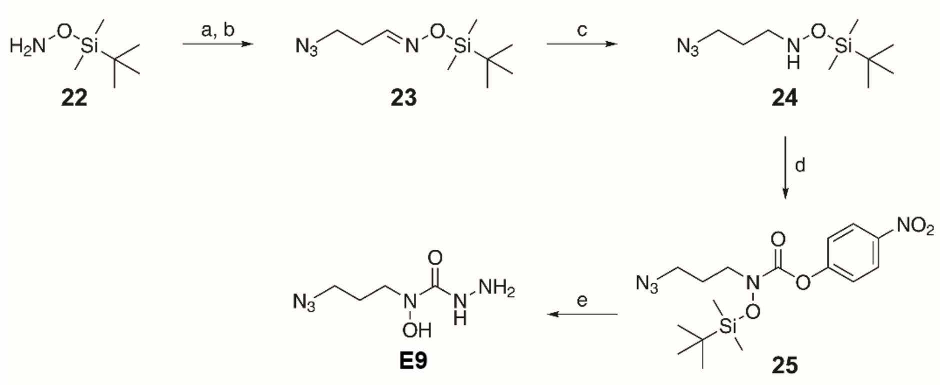

Scheme S10. Synthesis of **E9**. Reagents and conditions: a) acrolein, NaN_3, acetic acid, water; b) NaOAc, toluene (75%); c) $NaBH_3CN$, methanolic HCl, ethanol (43%); d) 4-nitrophenyl chloroformate, DiPEA, dichloromethane (77%); e) hydrazine hydrate, THF (23%).

(*E/Z*)-3-azidopropanal O-(*tert*-butyldimethylsilyl) oxime (23)

Acetic acid (1 mL) was cooled with an ice-salt bath between -15 °C to -20 °C, followed by addition of acrolein (0.3 mL, 4.5 mmol). Subsequently, a solution of NaN_3 (430 mg, 6.6 mmol) in water (1.3 mL) was added dropwise to the reaction mixture over the course of 2 minutes and the mixture was stirred at -15 °C to -20 °C for 1 h. The reaction mixture was quenched with aqueous $NaHCO_3$ (saturated, 25 mL) and extracted with toluene (25 mL, 3´). The combined organic layers were dried over $MgSO_4$ and filtered over cotton.

O-(tert-butyldimethylsilyl)hydroxylamine **22** (589 mg, 4.0 mmol) was added to the crude toluene solution, followed by addition of NaOAc (0.89 g, 10.8 mmol) and the mixture was stirred at room temperature overnight. The mixture was diluted with aqueous $NaHCO_3$ (saturated, 120 mL), the layers were separated and the aqueous layer was extracted with toluene (100 mL, 3×). All the organic layers were combined, dried over $MgSO_4$, filtered over cotton and concentrated under reduced pressure. Purification by column chromatography (dry-loaded on celite, 1% ether/pentane) yielded (*E/Z*)-3-azidopropanal O-(*tert*-

butyldimethylsilyl) oxime **23** (683 mg, 3.0 mmol, 75%) as a yellow oil. ^{1}H NMR (400 MHz, Chloroform-*d*) δ 7.53 (t, J = 5.2 Hz, ½ H), 6.93 (t, J = 5.2 Hz, ½ H), 3.52 – 3.42 (m, 2H), 2.67 (apperent q, J = 6.6 Hz, 1H), 2.49 (apparent q, J = 6.6 Hz, 1H), 0.93 (s, 9H), 0.17 (s, 3H), 0.16 (s, 3H). ^{13}C NMR (101 MHz, Chloroform-*d*) δ 151.8, 151.6, 48.4, 48.2, 29.7, 26.2, 26.1, 25.6, 18.3, 18.3, -5.2, -5.2. *HRMS measurements were carried out, but none of the attempted methods (ESI positive, APCI positive, and ESI negative) succeeded in ionization of **23**.*

***N*-(3-azidopropyl)-*O*-(*tert*-butyldimethylsilyl)hydroxylamine (24)**

Sodium cyanoborohydride (937 mg, 14.2 mmol) was added to a solution of **23** (683 mg, 3.0 mmol) in ethanol (10 mL), followed by addition of methanolic HCl (3 M, 1.2 mL, 3.6 mmol) and the reaction mixture was stirred at room temperature for 30 minutes. The mixture was concentrated under reduced pressure and diluted with aqueous $NaHCO_3$ (150 mL) and dichloromethane (100 mL). The layers were separated and the aqueous layer was extracted with dichloromethane (75 mL, 2×). All the organic layers were combined, dried over $MgSO_4$, filtered over cotton and concentrated under reduced pressure. Purification by column chromatography (dry-loaded on celite, 5→10% ether/pentane, then 100% ether) yielded *N*-(3-azidopropyl)-*O*-(*tert*-butyldimethylsilyl)hydroxylamine **24** (297 mg, 1.3 mmol, 43%) as a colorless oil. ^{1}H NMR (400 MHz, Chloroform-*d*) δ 5.09 (s, 1H), 3.37 (t, J = 6.8 Hz, 2H), 2.98 (t, J = 6.7 Hz, 2H), 1.79 (apparent p, J = 6.7 Hz, 2H), 0.91 (s, 9H), 0.09 (s, 6H). ^{13}C NMR (101 MHz, Chloroform-*d*) δ 51.5, 49.7, 26.8, 26.4, 18.2, -5.3. *HRMS measurements were carried out, but none of the attempted methods (ESI positive, APCI positive, and ESI negative) succeeded in detection of **24**.*

4-nitrophenyl (3-azidopropyl)((*tert*-butyldimethylsilyl)oxy)carbamate (25)

4-nitrophenyl chloroformate (210 mg, 1.0 mmol) and DiPEA (0.4 mL, 2.3 mmol) were added to a solution of **23** (116 mg, 0.50 mmol) in dichloromethane (5 mL) and the reaction mixture was stirred at room temperature overnight. The mixture was diluted with DCM (60 mL), washed with aqueous $KHSO_4$ (75 mL, 1×) and brine (75 mL, 1×), dried over $MgSO_4$, filtered over cotton and concentrated under reduced pressure. Purification by column chromatography (dry-loaded on celite, 10% ether/pentane) yielded 4-nitrophenyl (3-azidopropyl)((*tert*-butyldimethylsilyl)oxy)carbamate **25** (154 mg, 0.39 mmol, 77%) as a yellow oil. ^{1}H NMR (400 MHz, Chloroform-*d*) δ 8.27 (d, J = 9.0 Hz, 2H), 7.33 (d, J = 9.0 Hz, 2H), 3.73 (t, J = 6.6 Hz, 2H), 3.44 (t, J = 6.3 Hz, 2H), 2.03 (apparent p, J = 6.4 Hz, 2H), 0.98 (s, 9H), 0.25 (s, 6H). ^{13}C NMR (101 MHz, Chloroform-*d*) δ 155.8, 154.9, 145.3, 125.4, 122.1, 49.8, 49.0, 26.0, 25.8, 18.0, -4.8. HRMS (ESI-orbitrap) m/z calculated for $[M+H]^+$ 396.1698, found 396.1695.

***N*-(3-azidopropyl)-*N*-hydroxyhydrazinecarboxamide (E9)**

Hydrazine hydrate (0.3 mL, 6.2 mmol) was added to a solution of **25** (99 mg, 0.25 mmol) in THF (2.5 mL) and the reaction mixture was stirred vigorously for 3.5 h. The mixture was concentrated under reduced pressure and purified by column chromatography (dry-loaded on celite, 2→6% methanol/dichloromethane) to yield *N*-(3-azidopropyl)-*N*-hydroxyhydrazinecarboxamide **E9** (10 mg, 0.057 mmol, 23%) as a white solid. ^{1}H NMR (400 MHz, DMSO-d_6) δ 9.16 (s, 1H), 7.87 (s, 1H), 3.94 (s, 2H), 3.36 (m, 4H), 1.74 (apparent p, J = 6.6 Hz, 2H). ^{13}C NMR (101 MHz, DMSO-d_6) δ 162.3, 48.4, 47.9, 25.9. HRMS (ESI-orbitrap) m/z calculated for $[M+H]^+$ 175.0938, found 175.0938.

Synthesis of salicylic hydrazide E10

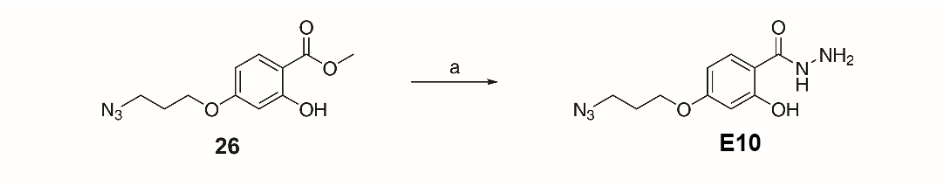

Scheme S11. Synthesis of **E10**. Reagents and conditions: a) hydrazine hydrate, methanol (quant)

4-(3-azidopropoxy)-2-hydroxybenzohydrazide (E10)

Hydrazine hydrate (0.23 ml, 7.5 mmol) was added to a suspension of 4-(3-azido-propoxy)-2-hydroxybenzoic acid methyl ester **26**[8] (47 mg, 0.19 mmol) in methanol (3 ml) and the reaction mixture was refluxed at 50 °C overnight. The mixture was concentrated under reduced pressure and co-evaporated with methanol (5×) to yield 4-(3-azidopropoxy)-2-hydroxybenzohydrazide **E10** as an off-white solid (48 mg, 0.19 mmol, quantitative). ^{1}H NMR (400 MHz, DMSO-*d6*) δ 9.92 (s, 1H), 7.72 (d, J = 8.7 Hz, 1H), 6.47 – 6.38 (m, 2H), 4.05 (t, J = 6.1 Hz, 2H), 3.49 (t, J = 6.7 Hz, 2H), 1.96 (m, 2H). ^{13}C NMR (101 MHz, DMSO-*d6*) δ 168.3, 162.4, 162.1, 128.2, 107.1, 106.2, 101.7, 64.8, 47.6, 28.0.

Synthesis of anthranilic hydrazide E11

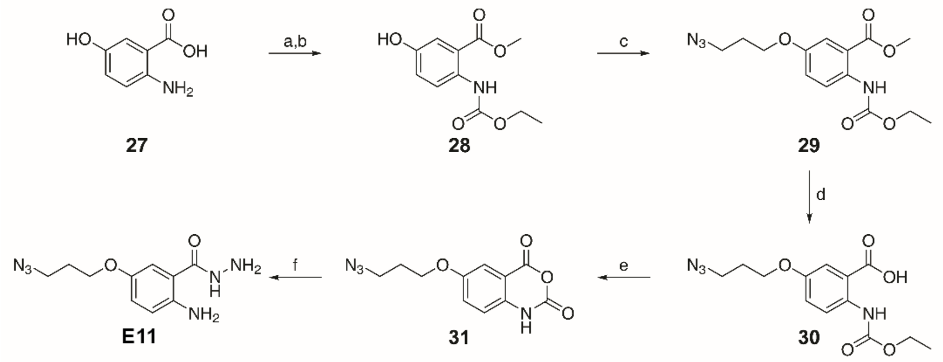

Scheme S12. Synthesis of **E11**. Reagents and conditions: a) ethyl chloroformate, dry dioxane, reflux; b) methyl iodide, K_2CO_3, DMF, 60 °C (50%); c) p-toluenesulfonic acid 3-azidopropyl ester, K_2CO_3, DMF, 60 °C (80%); d) KOH, water, dioxane (99%); e) oxalyl chloride, dry THF, reflux (70%); f) hydrazine hydrate, water (quantitative).

Methyl 2-((ethoxycarbonyl)amino)-5-hydroxybenzoate (28)

Ethyl chloroformate (1.2 mL, 12.8 mmol) was added to a suspension of 2-amino-5-hydroxybenzoic acid **27** (983 mg, 6.4 mmol) in dry dioxane (20 mL) under nitrogen atmosphere. The reaction mixture was heated at reflux for 6 h, followed by removal of the solvent under reduced pressure. The crude was dissolved in DMF (10 mL), then methyl iodide (0.38 mL, 6.1 mmol) and K_2CO_3 (1.35 g, 9.8 mmol) were added and the mixture was stirred at 60 °C overnight. The mixture was concentrated under reduced pressure, diluted with EtOAc (150 mL), washed with $NaHCO_3$ (saturated, 100 mL, 1×) and brine (100 mL, 1×), dried over $MgSO_4$, filtered over cotton and concentrated under reduced pressure. Purification by column chromatography (dry-loaded on celite, 20→40% ether/pentane) yielded methyl 2-((ethoxycarbonyl)amino)-5-hydroxybenzoate **28** (765 mg, 3.2 mmol, 50%) as an off-white solid. ^{1}H NMR (400 MHz, Chloroform-*d*) δ 10.13 (s, 1H), 8.29 (d, J = 9.1 Hz, 1H), 7.47 (d, J = 3.1 Hz, 1H), 7.05 (dd, J = 9.1, 3.1 Hz, 1H), 4.96 (m, J = 4.8 Hz, 1H), 4.21 (q, J = 7.1 Hz, 2H), 3.90 (s, 3H), 1.31 (t, J = 7.1 Hz, 3H). ^{13}C NMR (101 MHz, Chloroform-*d*) δ 168.1, 154.1, 149.9, 135.6, 122.2, 120.8, 116.7, 115.8, 61.3, 52.5, 14.7. HRMS (ESI-orbitrap) m/z calculated for $[M+Na]^+$ 262.0686, found 262.0686.

Methyl 5-(3-azidopropoxy)-2-((ethoxycarbonyl)amino)benzoate (29)

p-toluenesulfonic acid 3-azidopropyl ester (137 mg, 0.54 mmol) and K_2CO_3 (90 mg, 0.65 mmol) were added to a solution of **28** (100 mg, 0.42 mmol) in DMF (3 mL) and the reaction mixture was heated at 60 °C for 6 h. Additional *p*-toluenesulfonic acid 3-azidopropyl ester (73 mg, 0.29 mmol) and K_2CO_3 (49 mg, 0.36 mmol) were added and the mixture was stirred at 60 °C for another 2 h. The solvent was removed under reduced pressure and the crude was dissolved in EtOAc (75 mL), washed with $NaHCO_3$ (saturated, 60 mL, 1×) and brine (60 mL, 1×), dried over $MgSO_4$, filtered over cotton and concentrated under reduced pressure. Column chromatography (dry-loaded on celite, 8→10% ether/pentane) yielded methyl 5-(3-azidopropoxy)-2-((ethoxycarbonyl)amino)benzoate **29** (208 mg, 0.336 mmol, 80%, purity 52%) as a white solid. The impurity was the *p*-toluenesulfonic acid 3-azidopropyl ester starting material. *Note: a later repeat of this experiment revealed that the column chromatography step could have been omitted*. ^{1}H NMR (400 MHz, Chloroform-*d*) δ 10.16 (s, 1H), 8.35 (d, J = 9.2 Hz, 1H), 7.80 (d, J = 8.3 Hz, 2H; starting material), 7.50 (d, J = 3.1 Hz, 1H), 7.36 (d, J = 8.2 Hz, 2H; starting material), 7.12 (dd, J = 9.2, 3.1 Hz, 1H), 4.21 (q, J = 7.1 Hz, 2H), 4.11 (t, J = 5.9 Hz, 2H; starting material), 4.05 (t, J = 5.9 Hz, 2H), 3.92 (s, 3H), 3.52 (t, J = 6.6 Hz, 2H), 3.38 (t, J = 6.5 Hz, 2H; starting material), 2.46 (s, 3H; starting material), 2.05 (p, J = 6.3 Hz, 2H), 1.89 (p, J = 6.2 Hz, 2H; starting material), 1.31 (t, J = 7.1 Hz, 3H). ^{13}C NMR (101 MHz, Chloroform-*d*) δ 168.3, 154.0, 153.0, 145.2, 136.0, 132.9, 130.1, 128.1, 122.0, 120.6, 115.5, 115.3, 67.1, 65.2, 61.2, 52.5, 48.3, 47.4, 28.9, 28.6, 21.8, 14.7. HRMS (ESI-orbitrap) m/z calculated for $[M+Na]^+$ 345.1169, found 345.1168.

5-(3-azidopropoxy)-2-((ethoxycarbonyl)amino)benzoic acid (30)

29 (208 mg, 0.336 mmol, purity 52%) was dissolved in dioxane (2 mL), a solution of KOH (94 mg, 1.7 mmol) in water (2 mL) was added and the reaction mixture was stirred at room temperature for 1 h. The mixture was acidified with aqueous $KHSO_4$ (1 M, 50 mL), extracted with EtOAc (80 mL, 1×), then the organic layer was washed with brine (60 mL, 1×), dried over $MgSO_4$, filtered over cotton and concentrated under reduced pressure. The crude was dissolved in DCM (50 mL) and extracted with $NaHCO_3$ (50 mL, 2×). The combined aqueous layers were acidified to pH 3 with HCl (2 M, about 100 mL) and the resultant suspension was extracted with DCM (75 mL, 2×). The combined organic layers were dried over $MgSO_4$, filtered over cotton and concentrated under reduced pressure to yield 5-(3-azidopropoxy)-2-((ethoxycarbonyl)amino)benzoic acid **30** (106 mg, 0.34 mmol, 99%) as a white solid. ^{1}H NMR (400 MHz, Chloroform-*d*) δ 9.94 (s, 1H), 8.40 (d, J = 9.3 Hz, 1H), 7.58 (d, J = 3.1 Hz, 1H), 7.18 (dd, J = 9.3, 3.1 Hz, 1H), 4.24 (q, J = 7.1 Hz, 2H), 4.07 (t, J = 5.9 Hz, 2H), 3.53 (t, J = 6.6 Hz, 2H), 1.34 (t, J = 7.1 Hz, 3H). ^{13}C NMR (101 MHz, Chloroform-*d*) δ 171.4, 153.9, 153.1, 136.6, 123.3, 120.9, 115.9, 114.2, 65.2, 61.4, 48.3, 28.9, 14.7. HRMS (ESI-orbitrap) m/z calculated for $[M+Na]^+$ 331.1013, found 331.1011.

6-(3-azidopropoxy)-2H-benzo[d][1,3]oxazine-2,4(1H)-dione (31)

Oxalyl chloride (0.24 mL, 2.8 mmol) was added to a solution of **30** (106 mg, 0.34 mmol) in dry THF (3.5 mL) under nitrogen atmosphere and the reaction mixture was heated at reflux for 4 h. The mixture was concentrated under reduced pressure, suspended in diethyl ether (10 mL; effervescence was observed), sonicated for one minute and again concentrated under reduced pressure. Purification by column chromatography (dry-loaded on celite, 40% EtOAc/pentane, R_f = 0.33) yielded 6-(3-azidopropoxy)-2H-benzo[d][1,3]oxazine-2,4(1H)-dione **31** (63 mg, 0.24 mmol, 70%) as a white solid. ^{1}H NMR (400 MHz, DMSO-*d₆*) δ 11.60 (s, 1H), 7.39 (m, 2H), 7.35 (m, 1H), 7.11 (m, 1H), 4.09 (t, J = 5.5 Hz, 2H), 3.51 (t, J = 6.6 Hz, 2H), 1.98 (p, J = 6.2 Hz, 2H). ^{13}C NMR (101 MHz, DMSO-d_6) δ 159.8, 154.3, 146.9, 145.7, 135.6, 126.0, 117.0, 110.8, 65.5, 47.6, 28.0. HRMS (ESI-orbitrap) m/z calculated for $[M+Na]^+$ 285.0594, found 285.0593. Note: the m*ain peak observed was that of a methanol adduct minus CO_2, due to the use of methanol as the solvent for sample preparation for the HRMS. m/z calculated for $[M+H+CH_3OH-CO_2]^+$251.1139, found 251.1137.*

2-amino-5-(3-azidopropoxy)benzohydrazide (E11)

Water (1 mL) and hydrazine hydrate (1 mL, 20.6 mmol) were added to **31** (62 mg, 0.24 mmol) and the resultant suspension was stirred at room temperature overnight. The reaction mixture was concentrated under reduced pressure and co-evaporated with methanol (4×) to yield 2-amino-5-(3-azidopropoxy)benzohydrazide **E11** (61 mg, 0.24 mmol, quantitative) as an off-white solid. ^{1}H NMR (400 MHz, DMSO-d_6) δ 9.49 (s, 1H), 7.02 (d, J = 2.8 Hz, 1H), 6.82 (dd, J = 8.8, 2.8 Hz, 1H), 6.64 (d, J = 8.9 Hz, 1H), 5.93 (s, 2H), 4.38 (bs, 1H), 3.94 (t, J = 6.2 Hz, 2H), 3.49 (t, J = 6.7 Hz, 2H), 1.92 (p, J = 6.4 Hz, 2H). ^{13}C NMR (101 MHz, DMSO-d_6) δ 168.2, 148.3, 143.8, 120.3, 117.6, 113.7, 112.4, 65.2, 47.8, 28.2. HRMS (ESI-orbitrap) m/z calculated for $[M+Na]^+$ 273.1070, found 273.1070.

References

(1) Gu, H.; Ghosh, S.; Staples, R. J.; Bane, S. L. β-Hydroxy Stabilized Boron-Nitrogen Heterocycles Enable Rapid and Efficient C-Terminal Protein Modification. *Bioconjug. Chem.* **2019**, *30*, 2604–2613.

(2) Van Der Zouwen, A. J.; Lohse, J.; Wieske, L. H. E.; Hohmann, K. F.; Van Der Vlag, R.; Witte, M. D. An in Situ Combinatorial Methodology to Synthesize and Screen Chemical Probes. *Chem. Commun.* **2019**, *55*, 2050–2053.

(3) Zouwen, A. J.; Jeucken, A.; Steneker, R.; Hohmann, K. F.; Lohse, J.; Slotboom, D. J.; Witte, M. D. Iminoboronates as Dual-Purpose Linkers in Chemical Probe Development. *Chem. – A Eur. J.* **2021**, *27* (10), 3292–3296.

(4) Chen, X.; Parelkar, S. S.; Henchey, E.; Schneider, S.; Emrick, T. PolyMPC–Doxorubicin Prodrugs. *Bioconjug. Chem.* **2012**, *23* (9), 1753–1763.

(5) Jain, A.; Huang, S. G.; Whitesides, G. M. Lack of Effect of the Length of Oligoglycine- and Oligo(Ethylene Glycol)-Derived Para-Substituents on the Affinity of Benzenesulfonamides for Carbonic Anhydrase II in Solution. *J. Am. Chem. Soc.* **1994**, *116* (12), 5057–5062.

(6) Li, F.-Y.; Liu, J.-B.; Gong, J.-N.; Li, G. (R)-2-Phenyl-4,5-Dihydrothiazole-4-Carboxamide Derivatives Containing a Diacylhydrazine Group: Synthesis, Biological Evaluation, and SARs. *Molecules* **2019**, *24* (24), 4440.

(7) Nakatani, S.; Ikura, M.; Yamamoto, S.; Nishita, Y.; Itadani, S.; Habashita, H.; Sugiura, T.; Ogawa, K.; Ohno, H.; Takahashi, K.; et al. Design and Synthesis of Novel Metalloproteinase Inhibitors. *Bioorg. Med. Chem.* **2006**, *14* (15), 5402–5422.

(8) Guizzunti, G.; Brady, T. P.; Malhotra, V.; Theodorakis, E. A. Trifunctional Norrisolide Probes for the Study of Golgi Vesiculation. *Bioorg. Med. Chem. Lett.* **2007**, *17* (2), 320–325.

(9) Steinhilber, D.; Rossow, T.; Wedepohl, S.; Paulus, F.; Seiffert, S.; Haag, R. A Microgel Construction Kit for Bioorthogonal Encapsulation and PH-Controlled Release of Living Cells. *Angew. Chem. Int. Ed.* **2013**, *52* (51), 13538–13543.

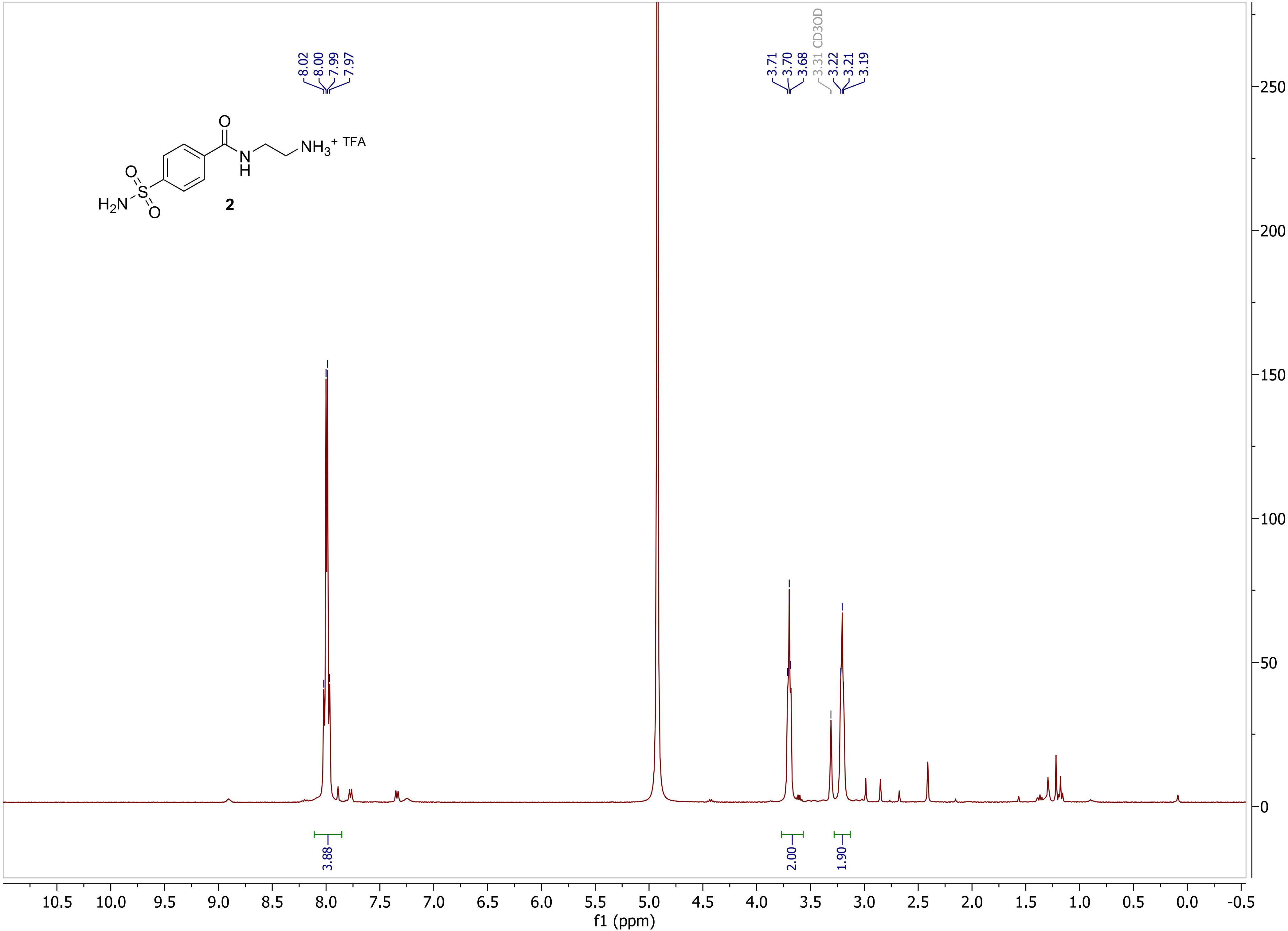
8.02
8.00
7.99
7.97
3.71
3.70
3.68
3.31 CD3OD
3.22
3.21
3.19
O
N
H
NH3+ TFA
O
S
O
H2N
2
3.88
2.00
1.90
250
200
150
100
50
0
10.5 10.0 9.5 9.0 8.5 8.0 7.5 7.0 6.5 6.0 5.5 5.0 4.5 4.0 3.5 3.0 2.5 2.0 1.5 1.0 0.5 0.0 -0.5
f1 (ppm)

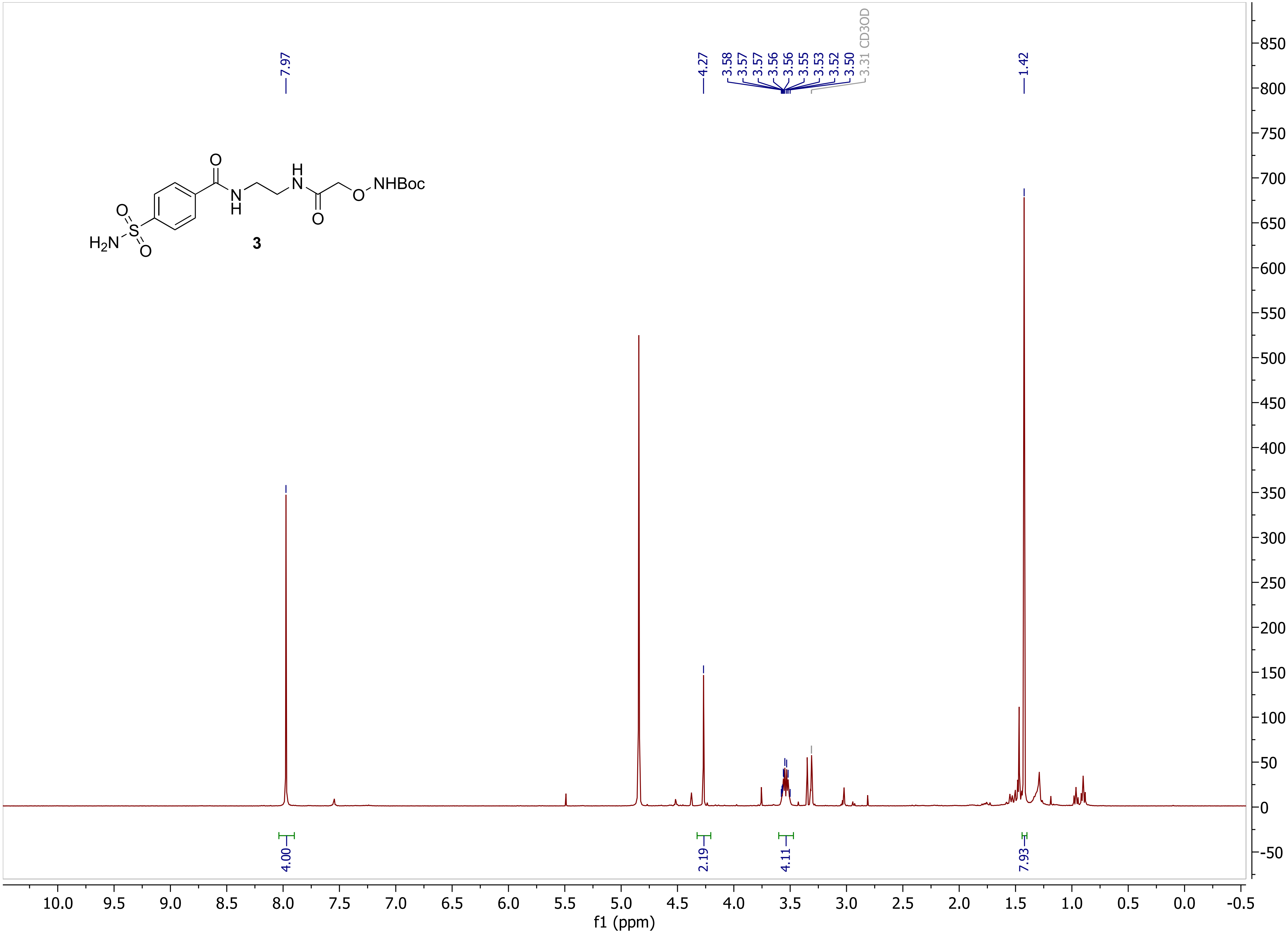

7.97
4.27
3.58
3.57
3.57
3.56
3.56
3.55
3.53
3.52
3.50
3.31 CD3OD
1.42
NHBoc
3
4.00
2.19
4.11
7.93
f1 (ppm)

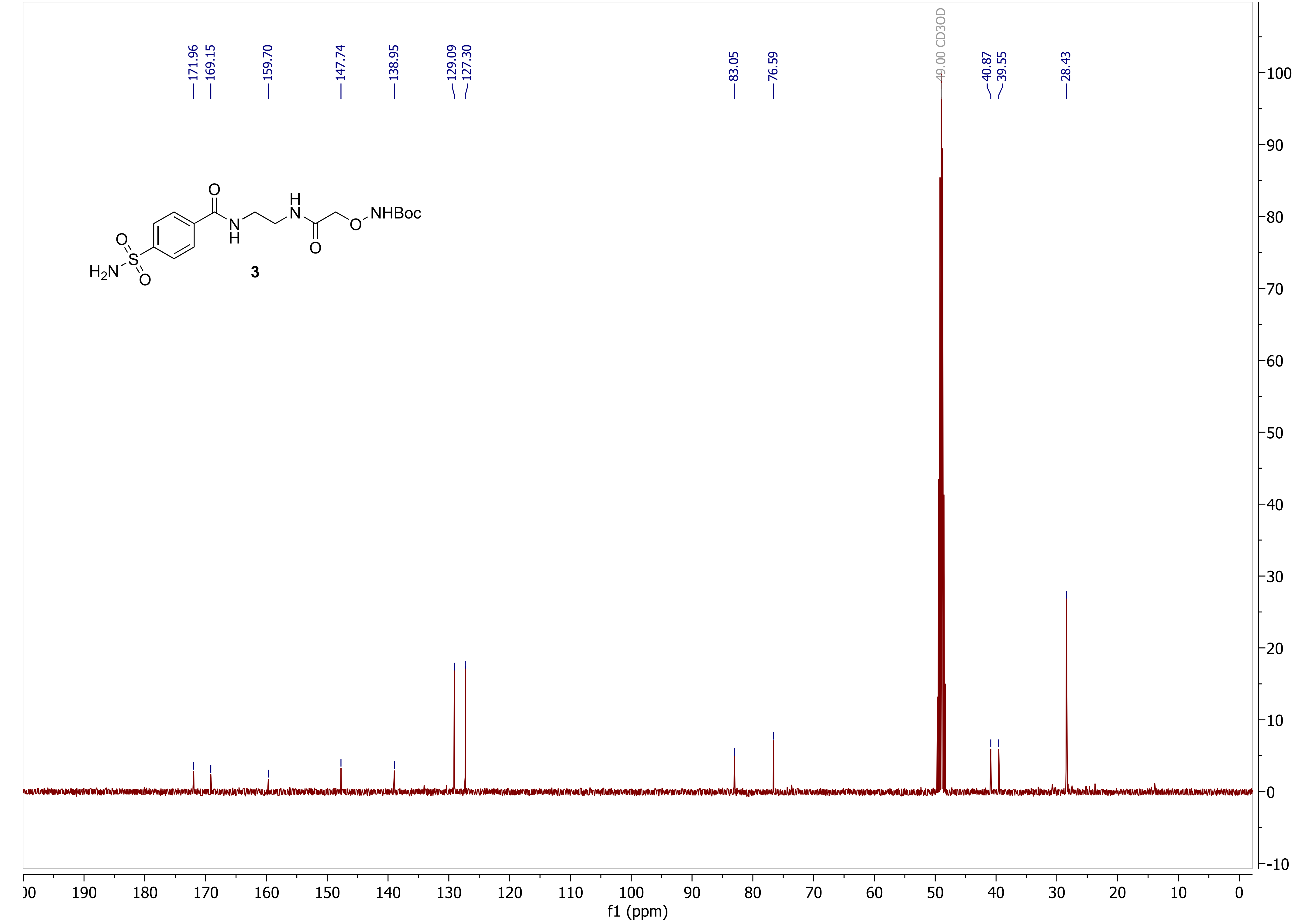
171.96
169.15
159.70
147.74
138.95
129.09
127.30
83.05
76.59
49.00 CD3OD
40.87
39.55
28.43
NHBoc
H_2N
3
f1 (ppm)

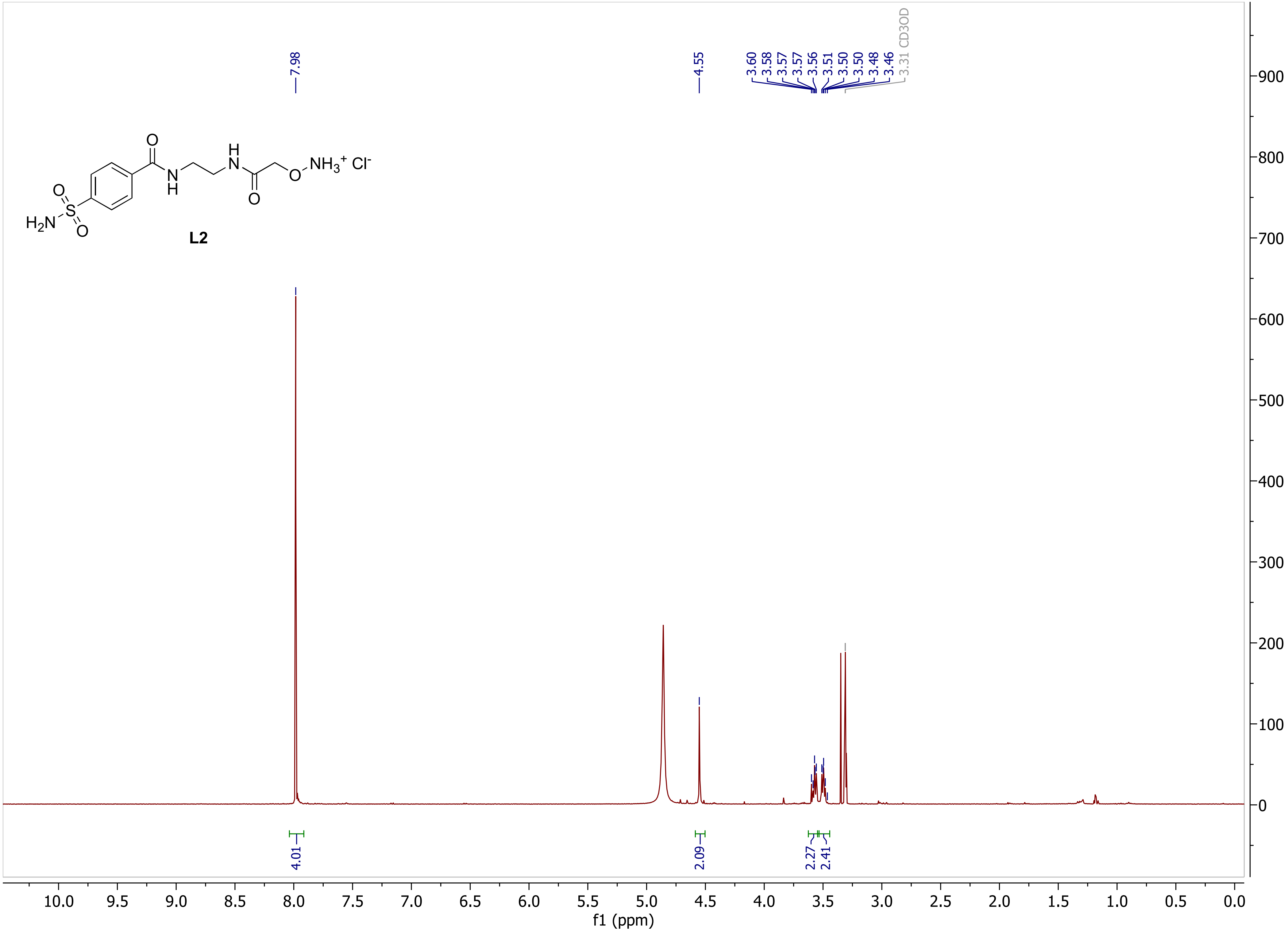
L2
7.98
4.55
3.60 3.58 3.57 3.57 3.56 3.51 3.50 3.50 3.48 3.46
3.31 CD3OD
4.01
2.09
2.27
2.41
10.0 9.5 9.0 8.5 8.0 7.5 7.0 6.5 6.0 5.5 5.0 4.5 4.0 3.5 3.0 2.5 2.0 1.5 1.0 0.5 0.0
f1 (ppm)
900 800 700 600 500 400 300 200 100 0

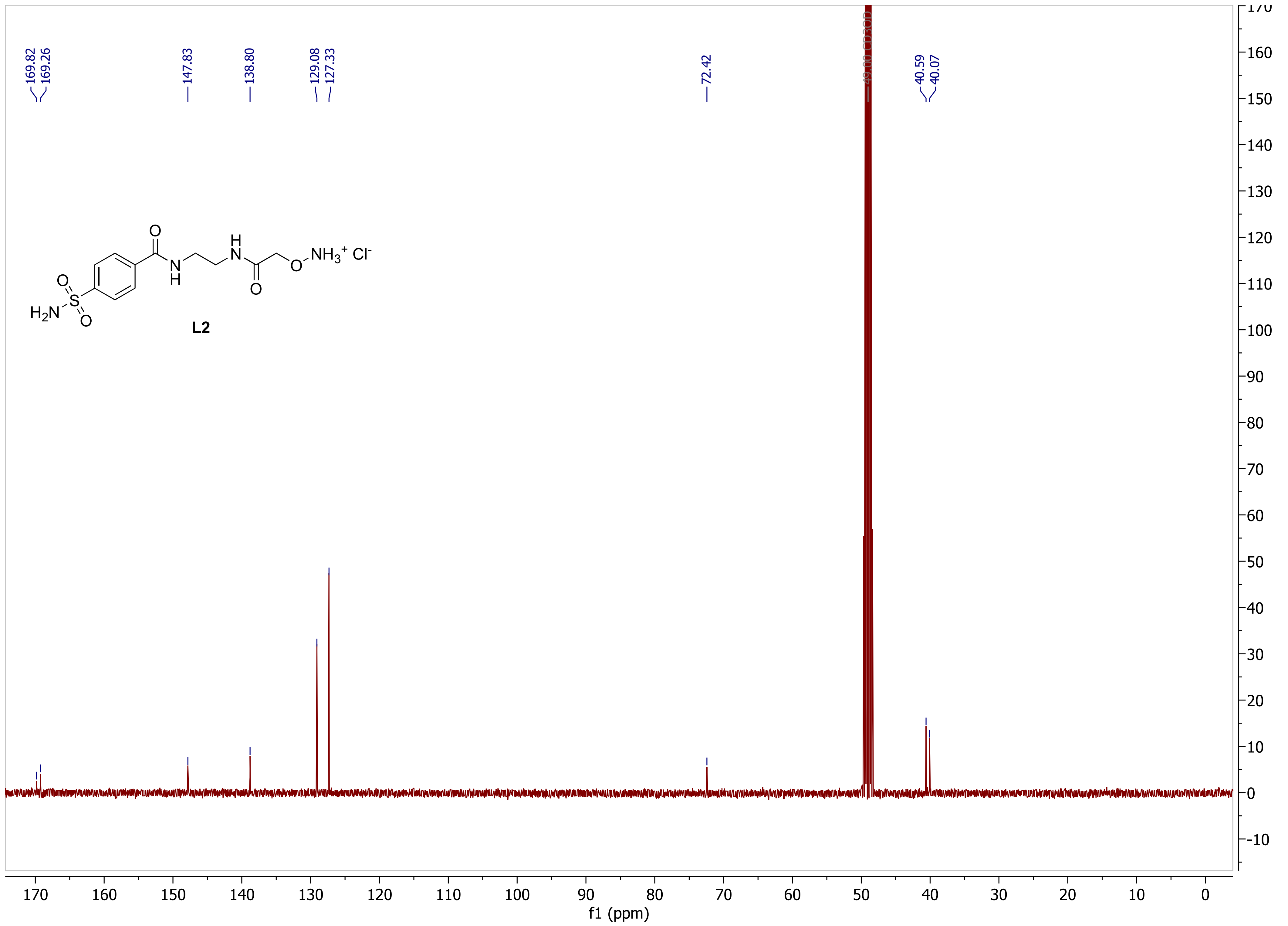

169.82
169.26
147.83
138.80
129.08
127.33
72.42
49.00 CD3OD
40.59
40.07
L2
f1 (ppm)

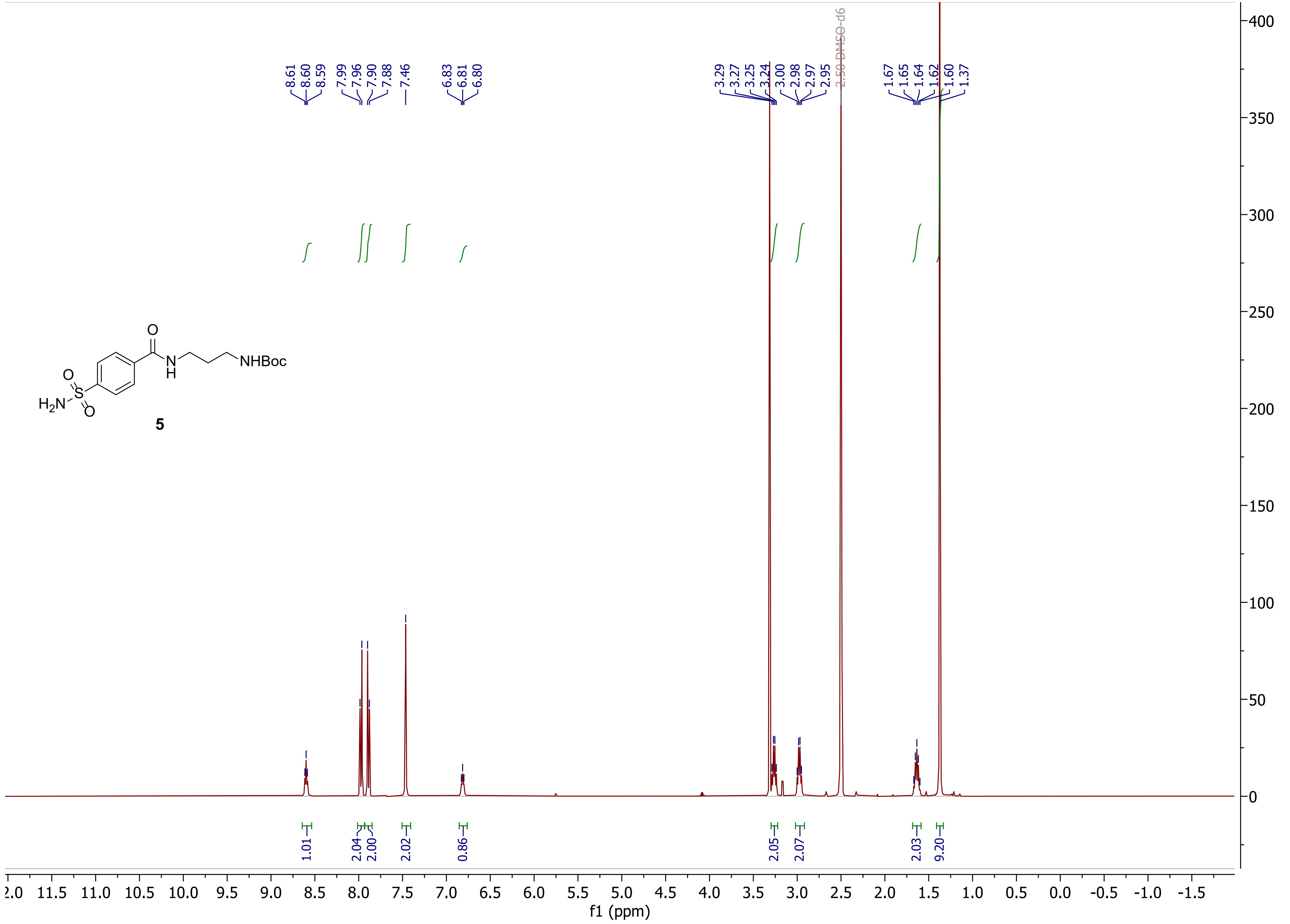

8.61
8.60
8.59
7.99
7.96
7.90
7.88
7.46
6.83
6.81
6.80
3.29
3.27
3.25
3.24
3.00
2.98
2.97
2.95
2.50 DMSO-d6
1.67
1.65
1.64
1.62
1.60
1.37
1.01
2.04
2.00
2.02
0.86
2.05
2.07
2.03
9.20
O
N
H
NHBoc
O
S
O
H2N
5
f1 (ppm)

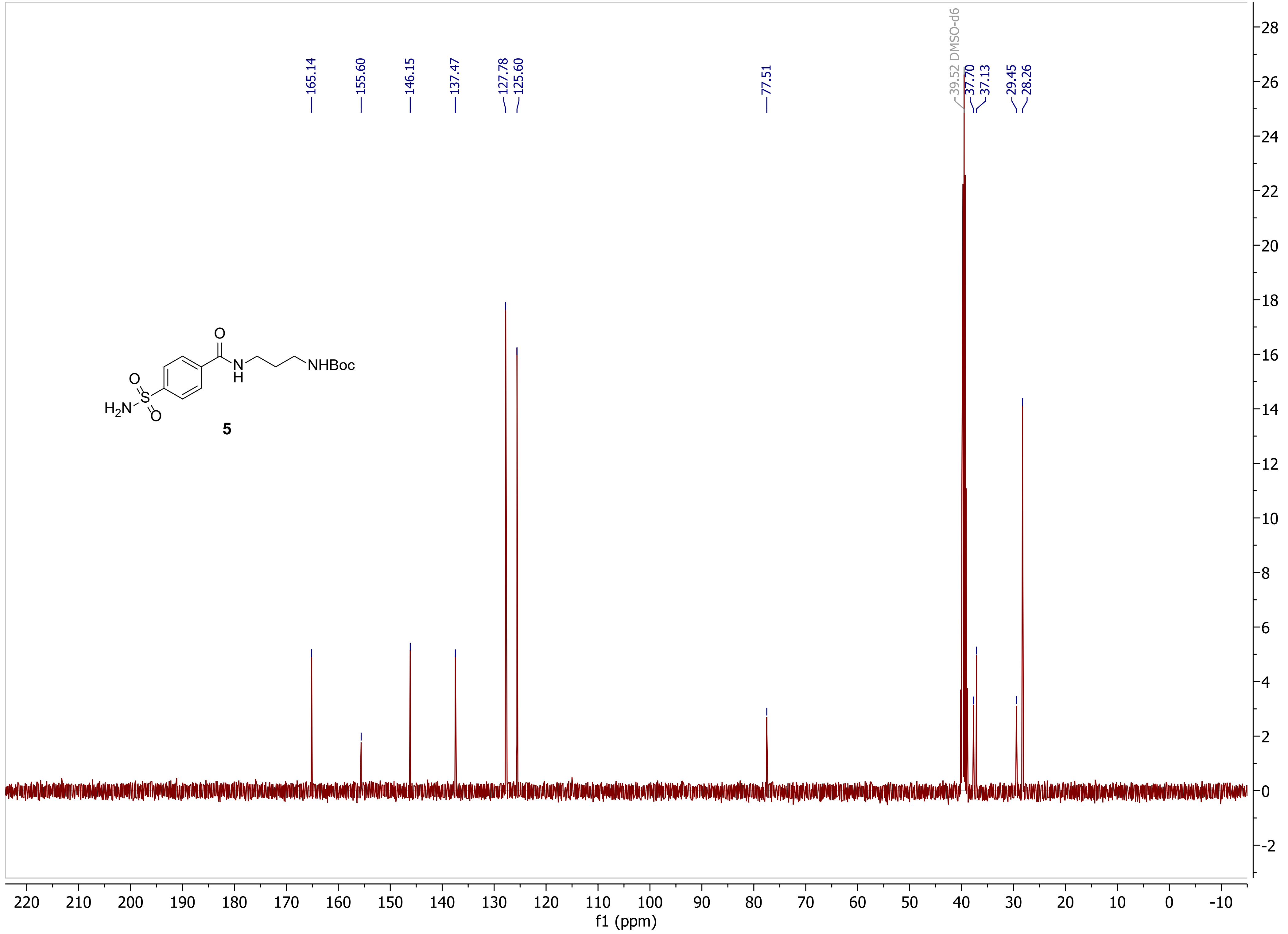
165.14
155.60
146.15
137.47
127.78
125.60
77.51
39.52 DMSO-d6
37.70
37.13
29.45
28.26
O
N
H
NHBoc
O
S
H2N
O
5
f1 (ppm)

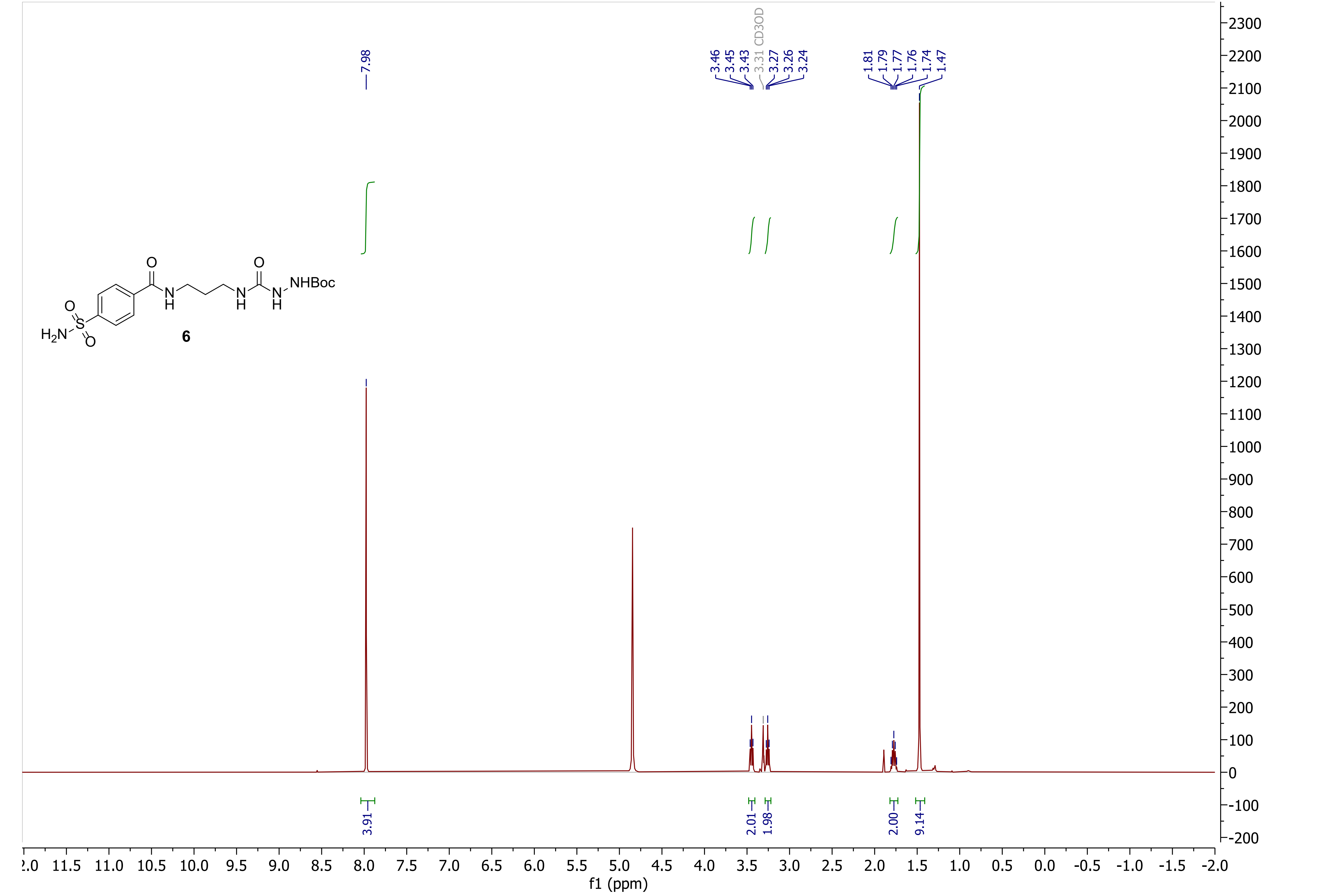

7.98
3.46
3.45
3.43
3.31 CD3OD
3.27
3.26
3.24
1.81
1.79
1.77
1.76
1.74
1.47
NHBoc
6
3.91
2.01
1.98
2.00
9.14
f1 (ppm)

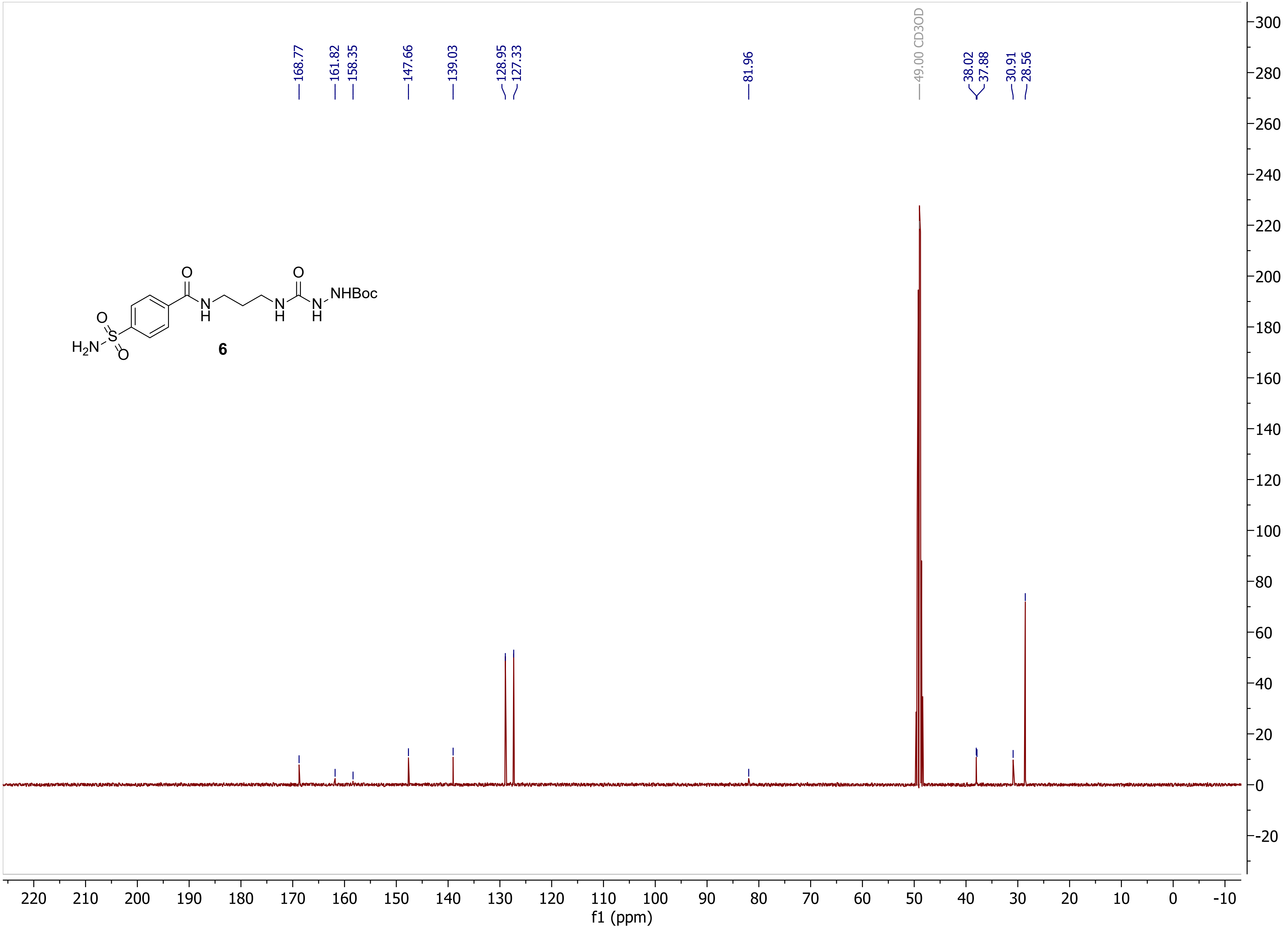

168.77
161.82
158.35
147.66
139.03
128.95
127.33
81.96
49.00 CD3OD
38.02
37.88
30.91
28.56
NHBoc
6
f1 (ppm)

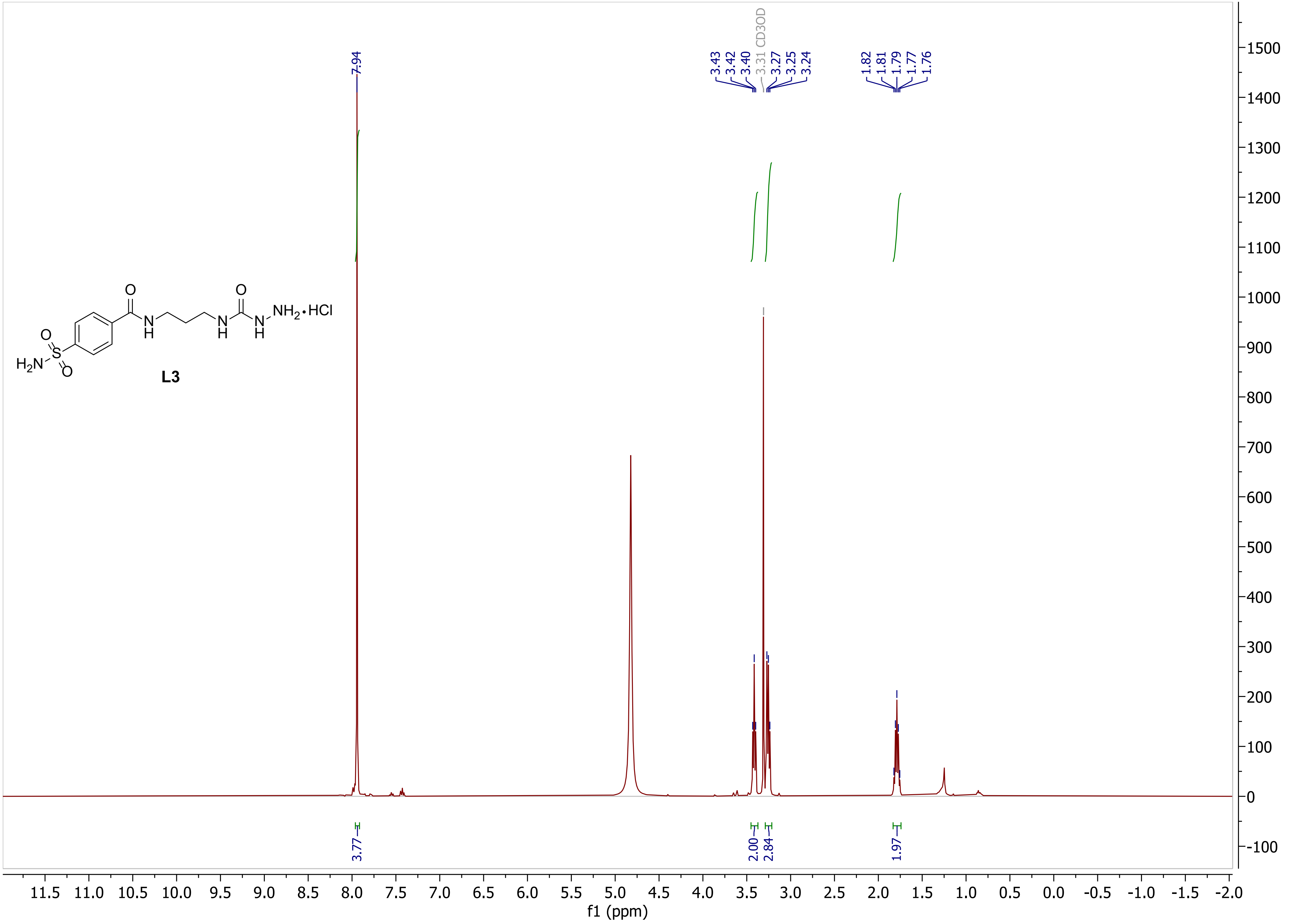
7.94
3.43
3.42
3.40
3.31 CD3OD
3.27
3.25
3.24
1.82
1.81
1.79
1.77
1.76
3.77
2.00
2.84
1.97
NH2•HCl
H2N
L3
f1 (ppm)

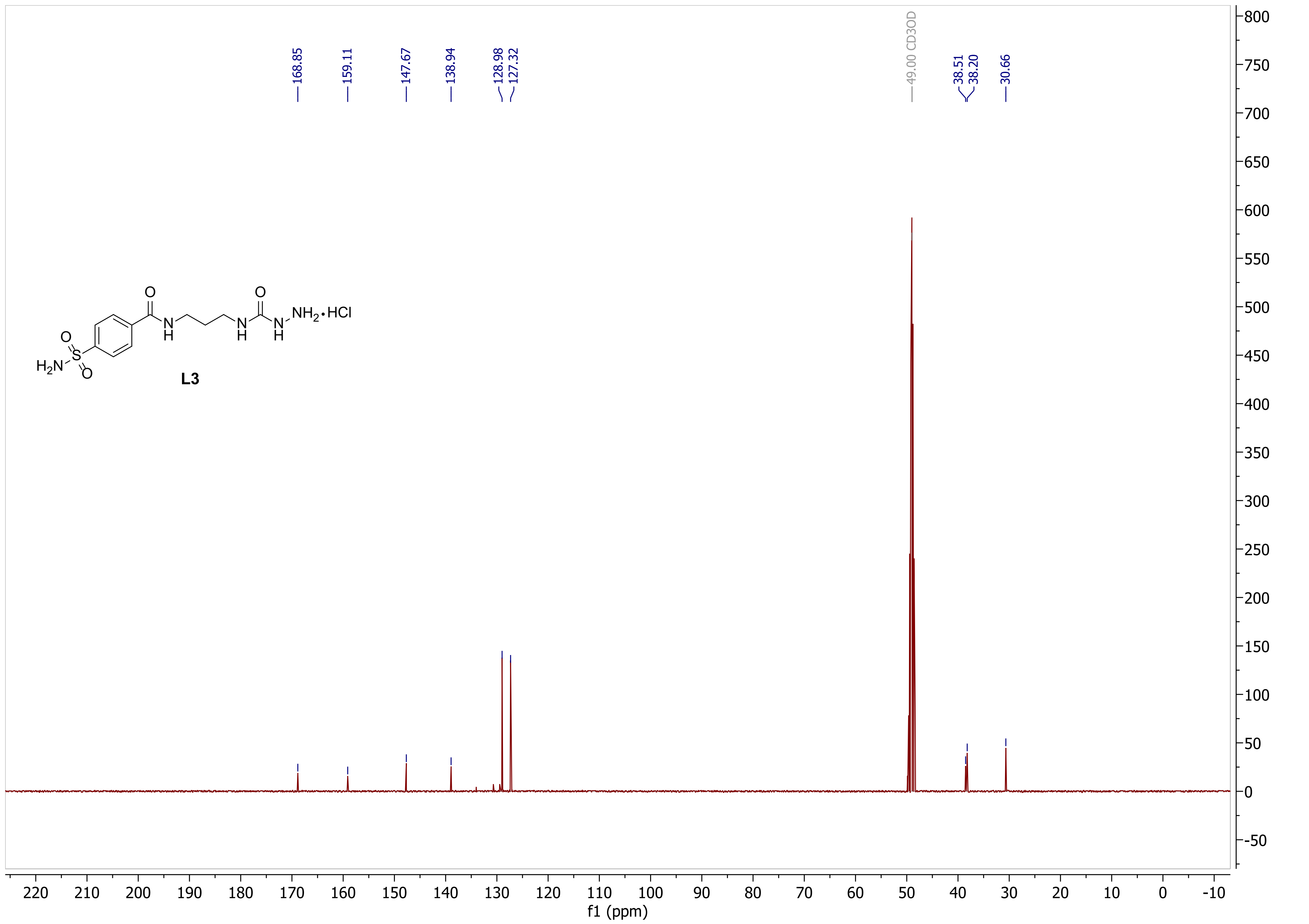

168.85
159.11
147.67
138.94
128.98
127.32
49.00 CD3OD
38.51
38.20
30.66
O
O
H
N
N
H
N
H
NH2•HCl
O
O
S
H2N
L3
800
750
700
650
600
550
500
450
400
350
300
250
200
150
100
50
0
-50
220 210 200 190 180 170 160 150 140 130 120 110 100 90 80 70 60 50 40 30 20 10 0 -10
f1 (ppm)

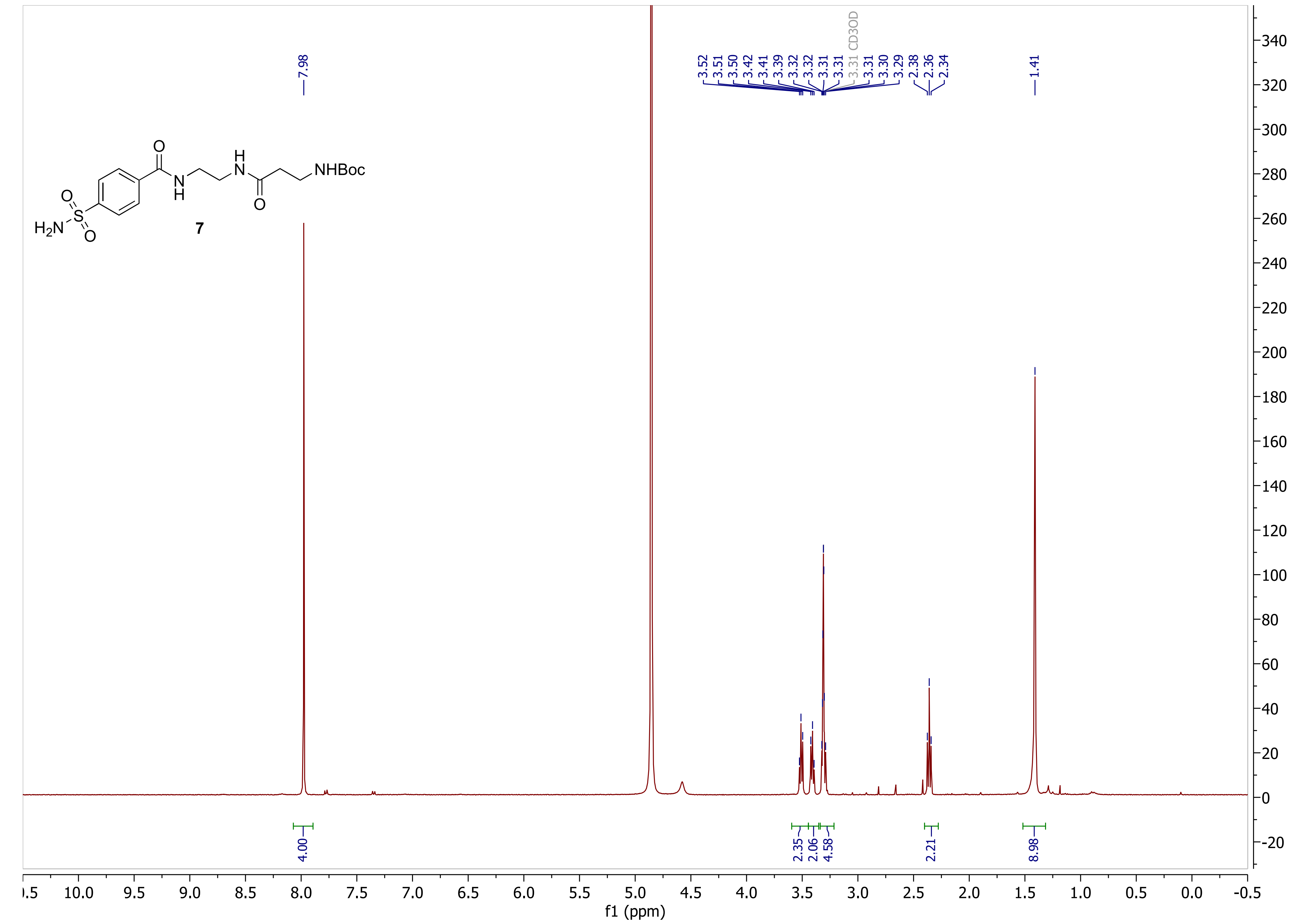
7.98
3.52 3.51 3.50 3.42 3.41 3.39 3.32 3.32 3.31 3.31 3.31 CD3OD 3.31 3.30 3.29 2.38 2.36 2.34
1.41
NHBoc
H2N
7
4.00
2.35 2.06 4.58
2.21
8.98
f1 (ppm)

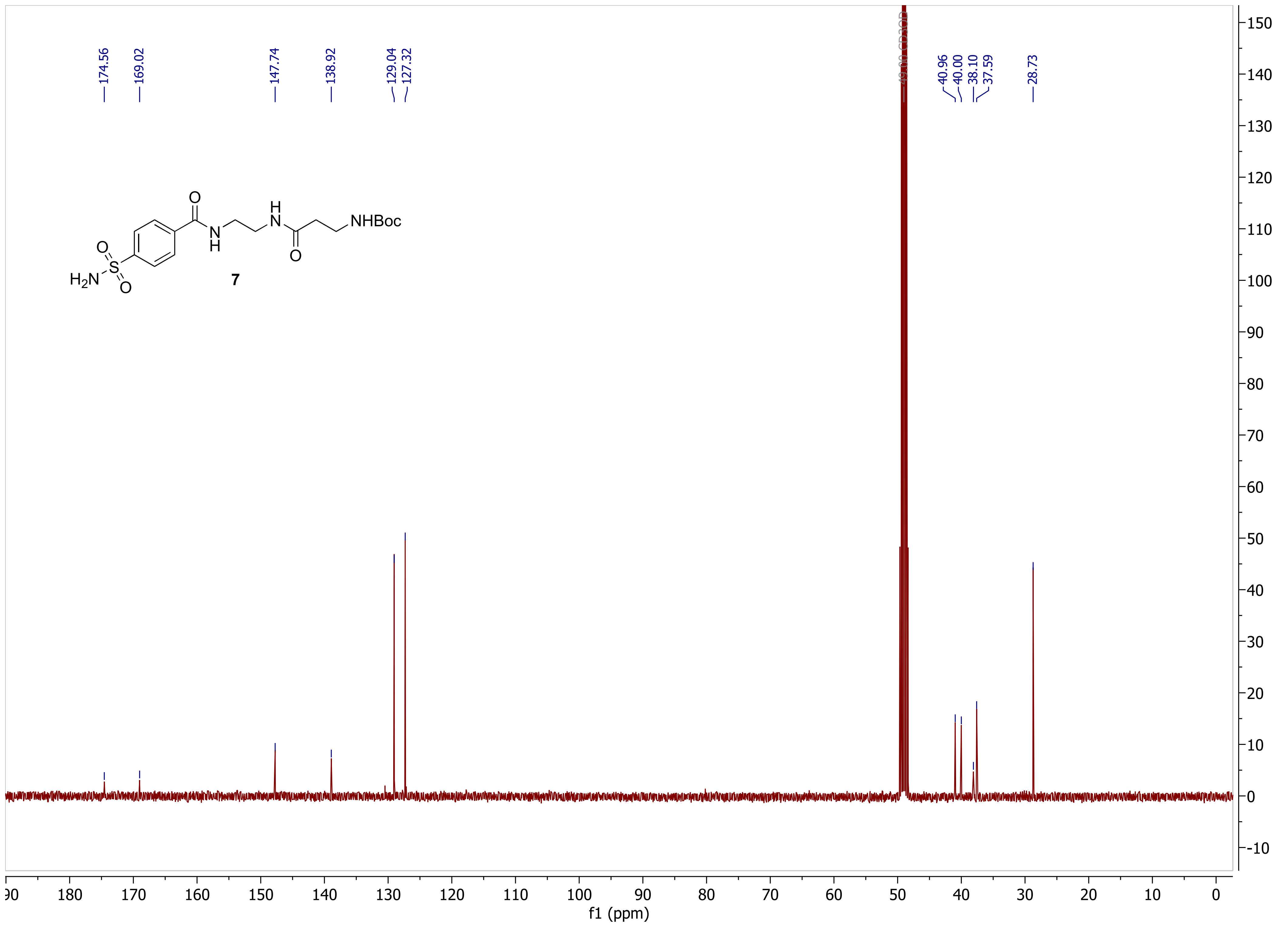

174.56
169.02
147.74
138.92
129.04
127.32
40.96
40.00
38.10
37.59
28.73
O
H
N
NHBoc
N
H
O
O
S
H2N
O
7
f1 (ppm)

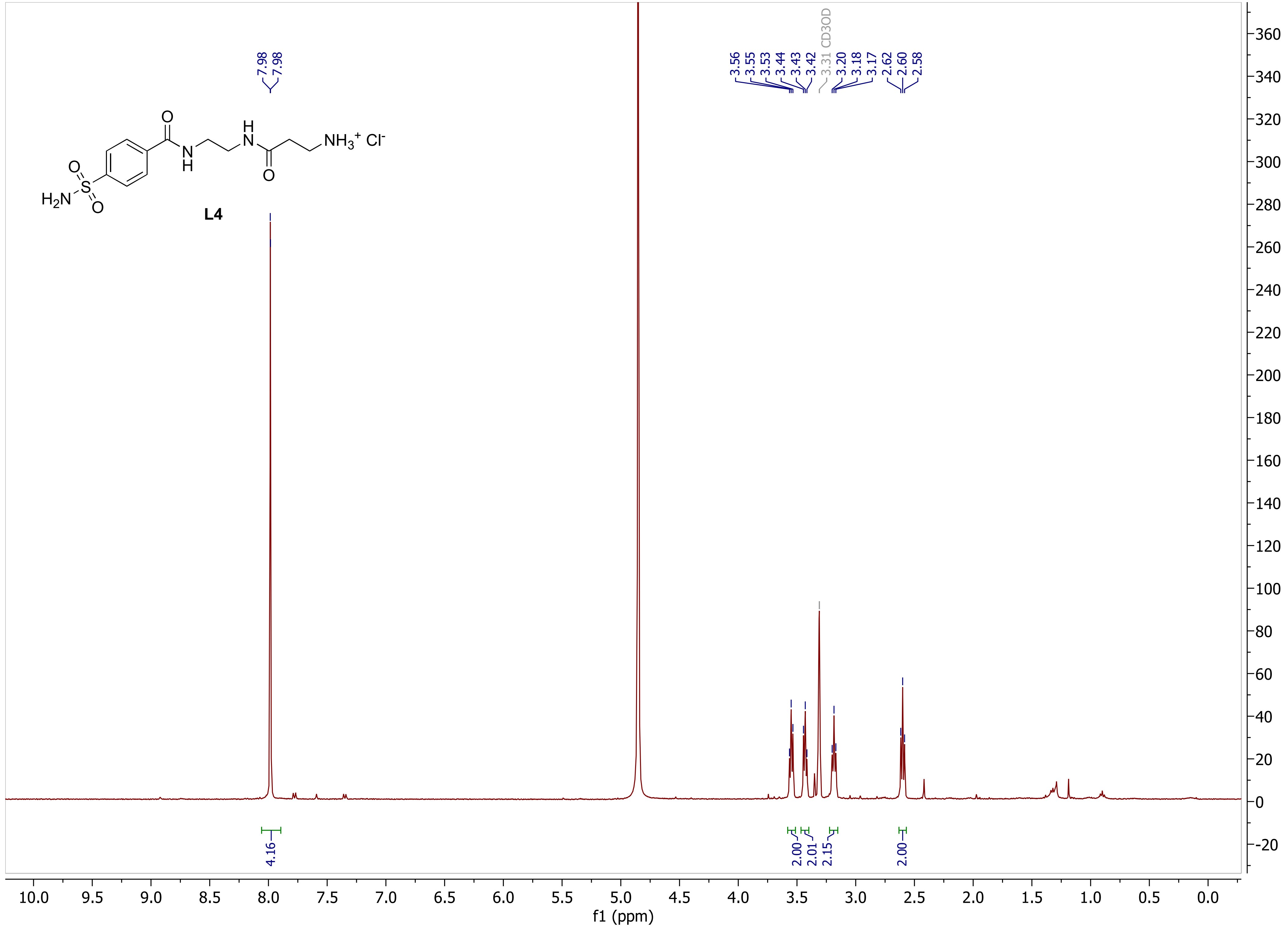
7.98
7.98
3.56
3.55
3.53
3.44
3.43
3.42
3.31 CD3OD
3.20
3.18
3.17
2.62
2.60
2.58
L4
4.16
2.00
2.01
2.15
2.00
f1 (ppm)

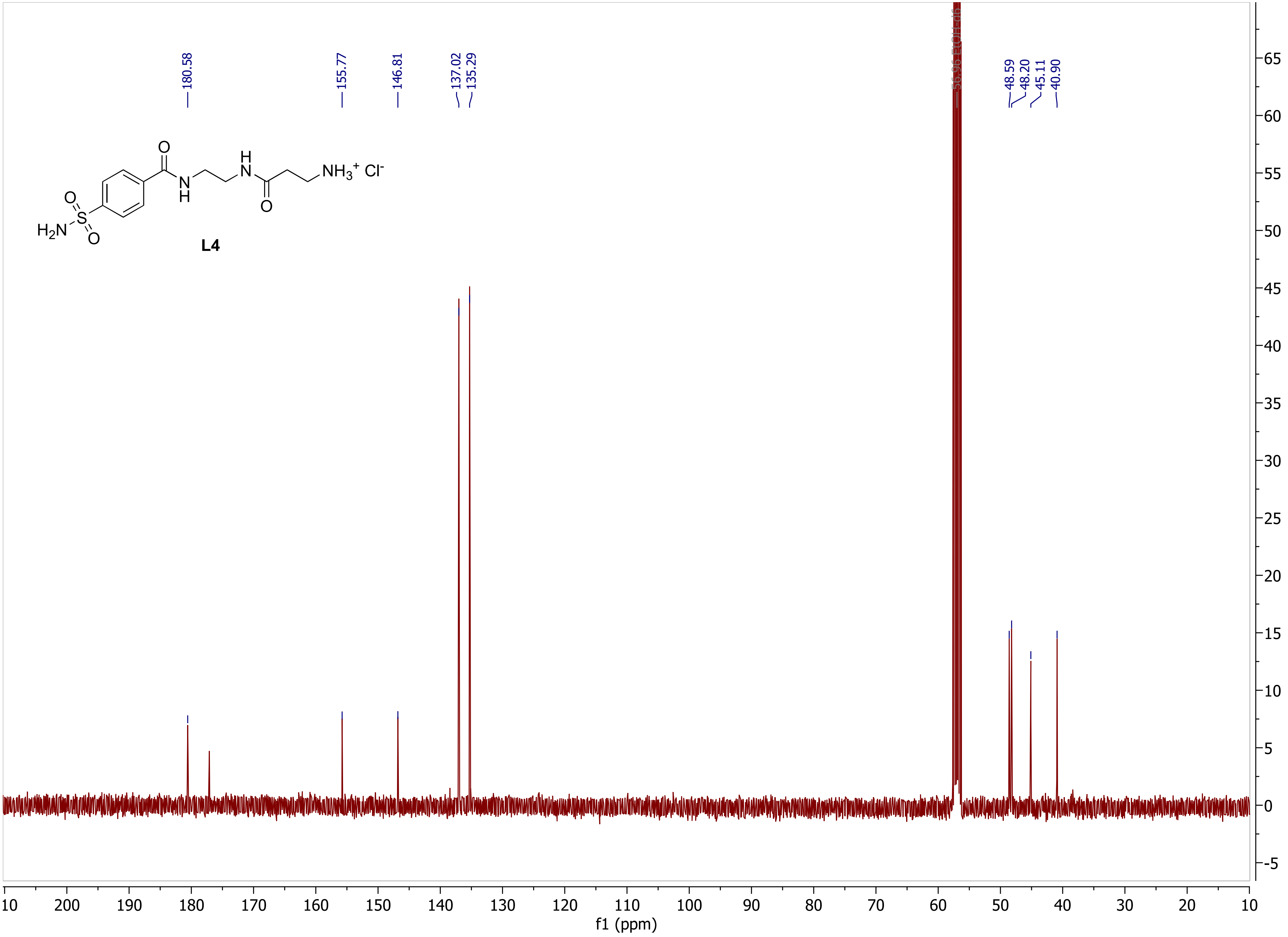
180.58
155.77
146.81
137.02
135.29
56.96 EtOH-d6
48.59
48.20
45.11
40.90
L4
f1 (ppm)

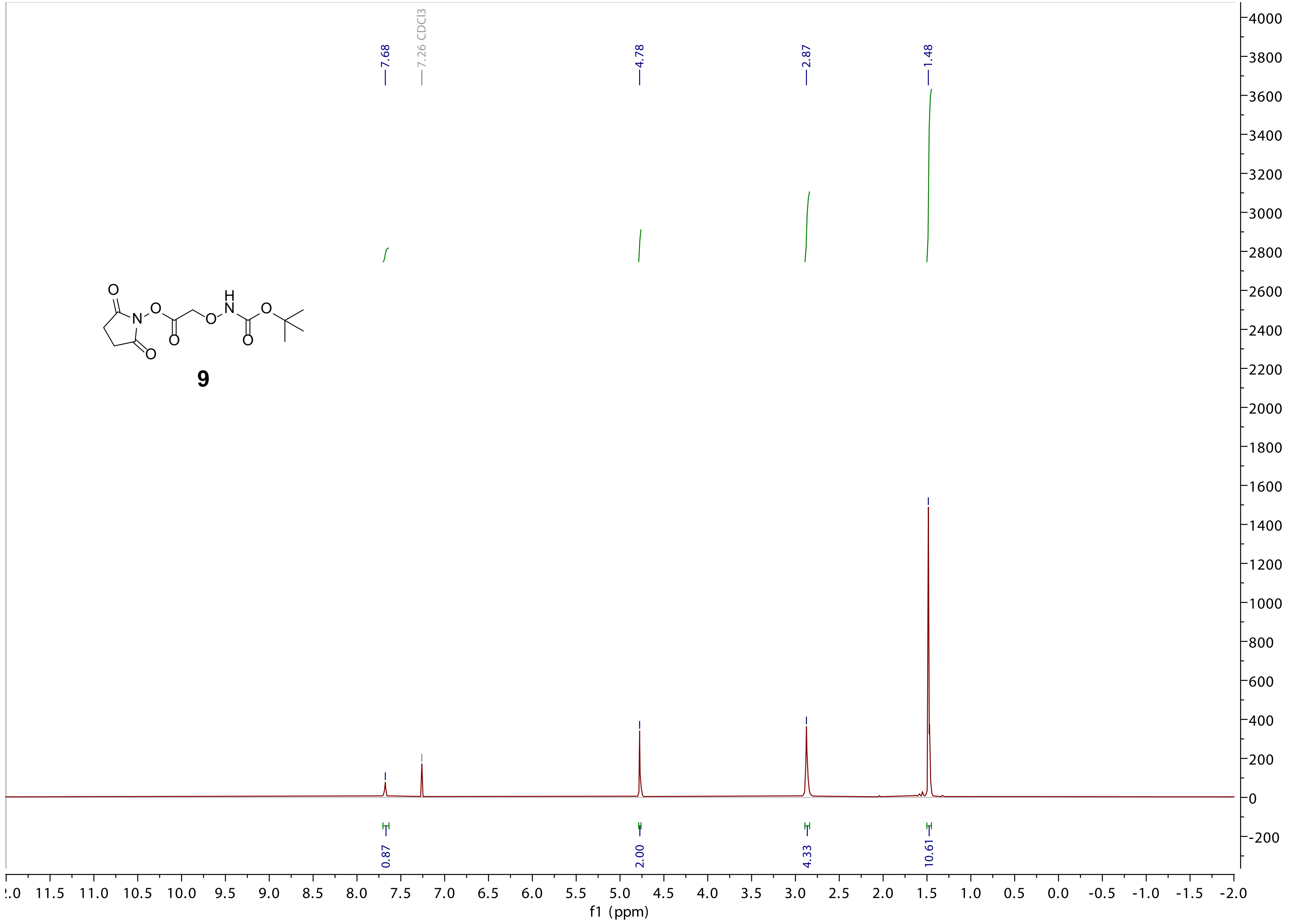
7.68
7.26 CDCl3
4.78
2.87
1.48
0.87
2.00
4.33
10.61
9
f1 (ppm)

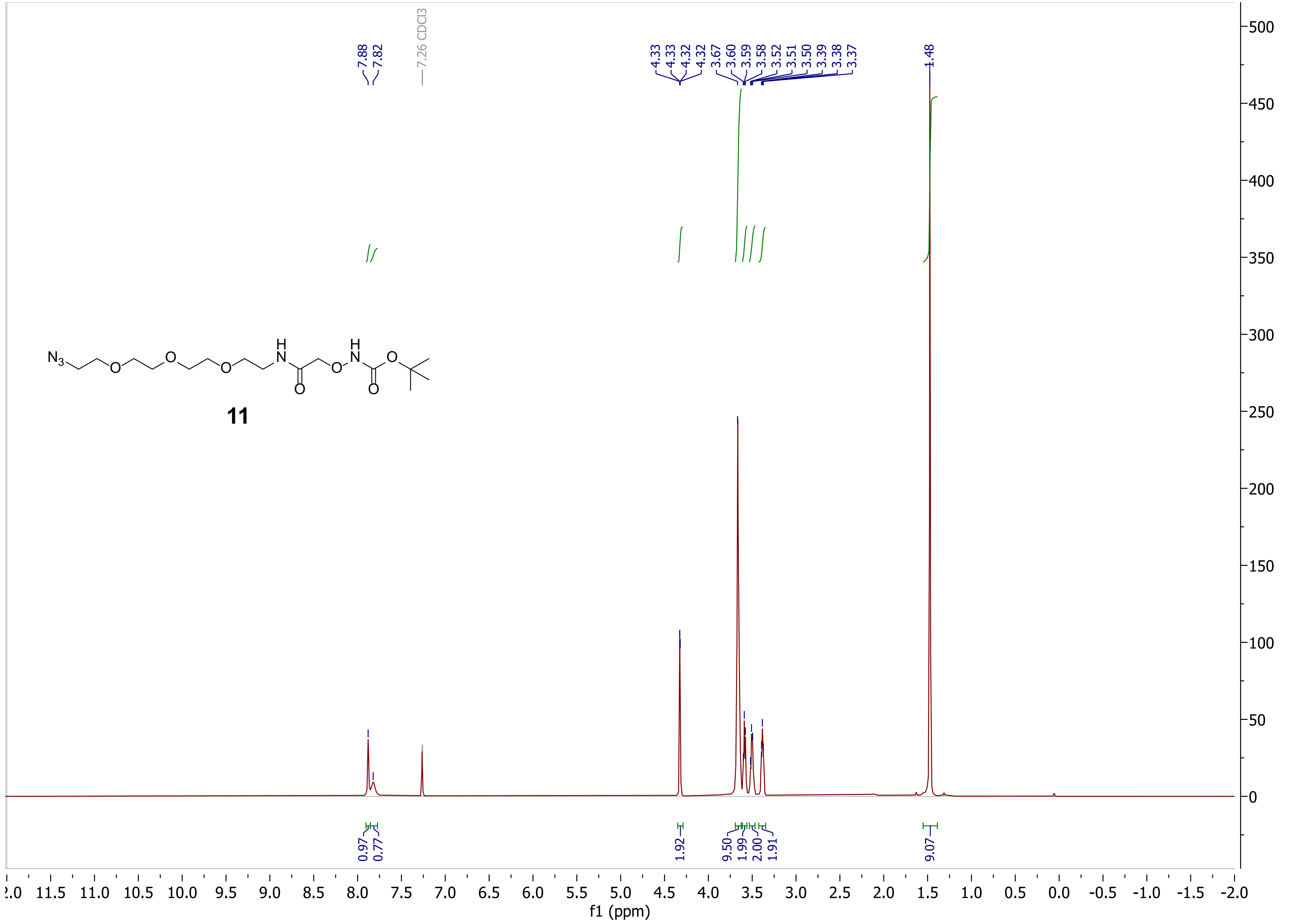

7.88
7.82
7.26 CDCl3
4.33
4.33
4.32
4.32
3.67
3.60
3.59
3.58
3.52
3.51
3.50
3.39
3.38
3.37
1.48
N_3
O
H
N
11
0.97
0.77
1.92
9.50
1.99
2.00
1.91
9.07
f1 (ppm)

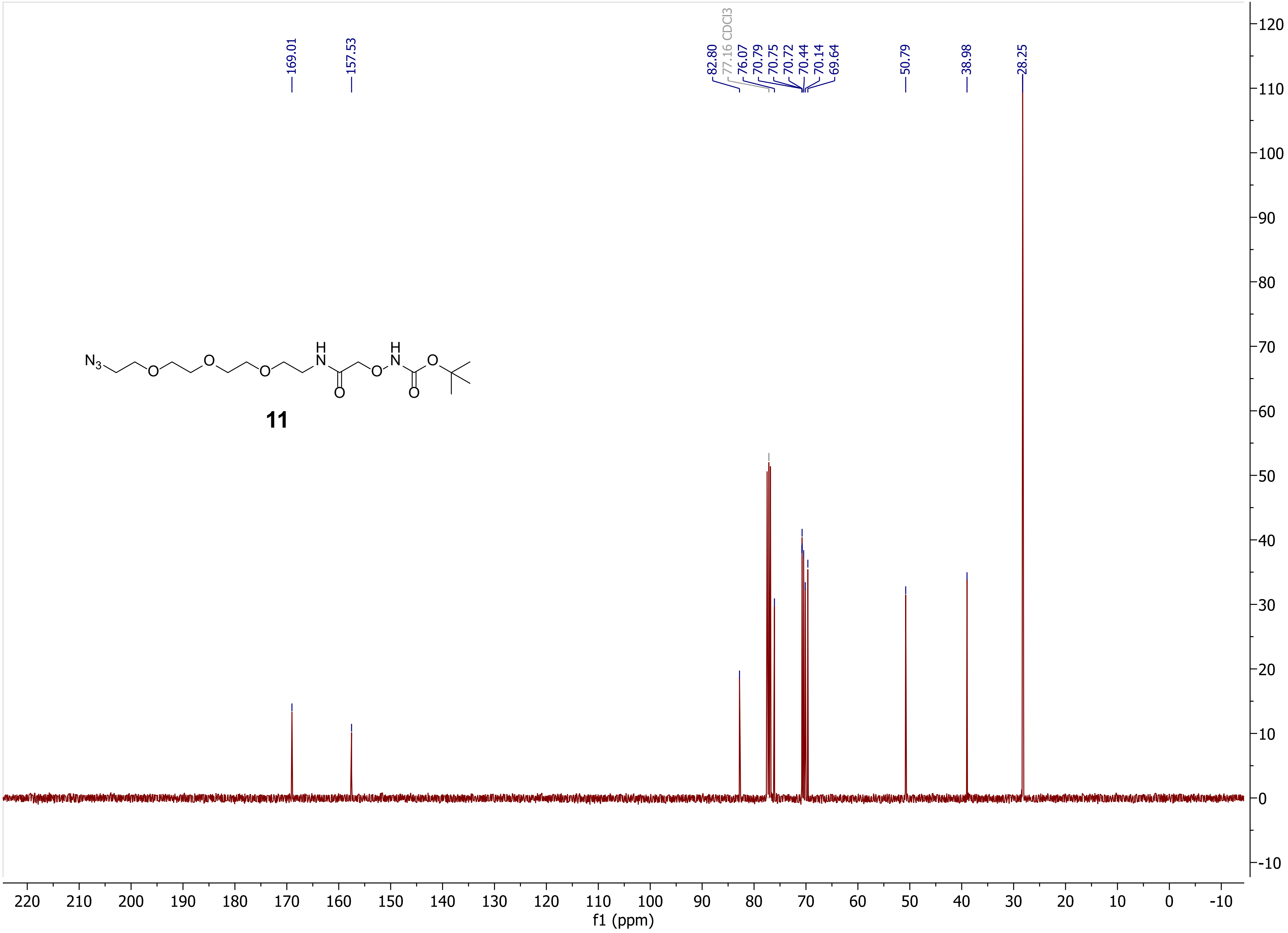
169.01
157.53
82.80
77.16 CDCl3
76.07
70.79
70.75
70.72
70.44
70.14
69.64
50.79
38.98
28.25
11
f1 (ppm)

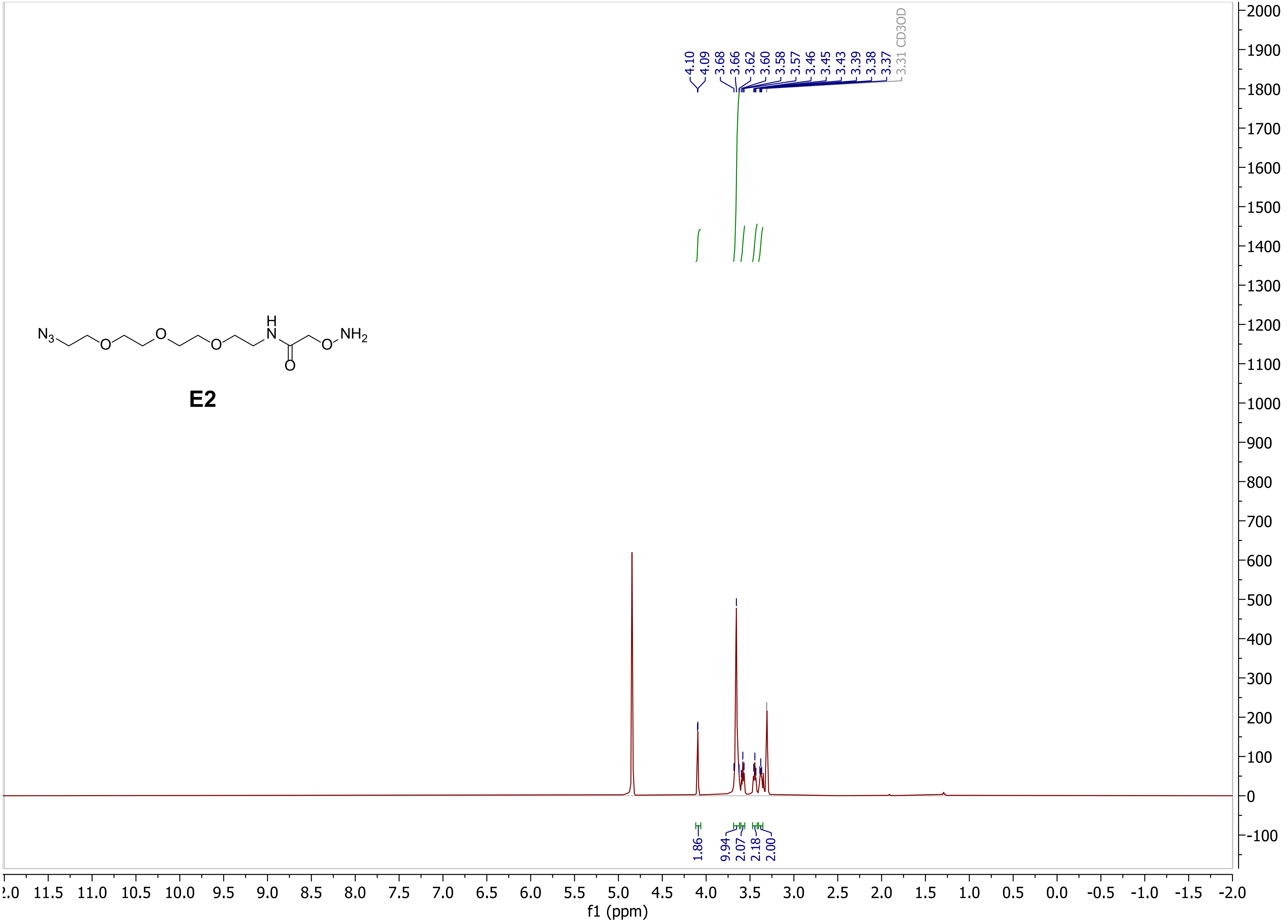

4.10
4.09
3.68
3.66
3.62
3.60
3.58
3.57
3.46
3.45
3.43
3.39
3.38
3.37
3.31 CD3OD
1.86
9.94
2.07
2.18
2.00
f1 (ppm)
E2

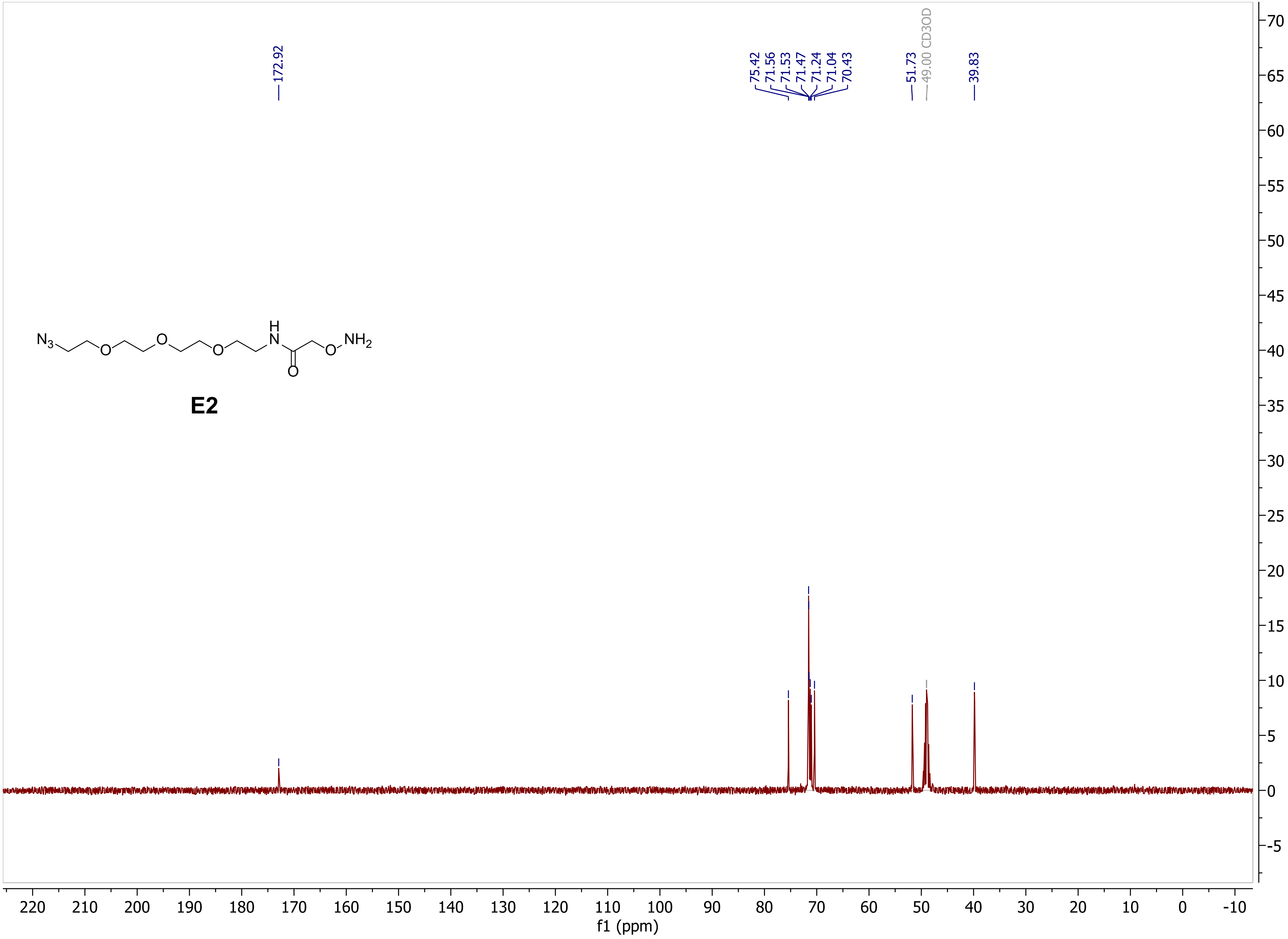
172.92
75.42
71.56
71.53
71.47
71.24
71.04
70.43
51.73
49.00 CD3OD
39.83
N3
O
O
O
H
N
O
O
NH2
E2
70
65
60
55
50
45
40
35
30
25
20
15
10
5
0
-5
220 210 200 190 180 170 160 150 140 130 120 110 100 90 80 70 60 50 40 30 20 10 0 -10
f1 (ppm)

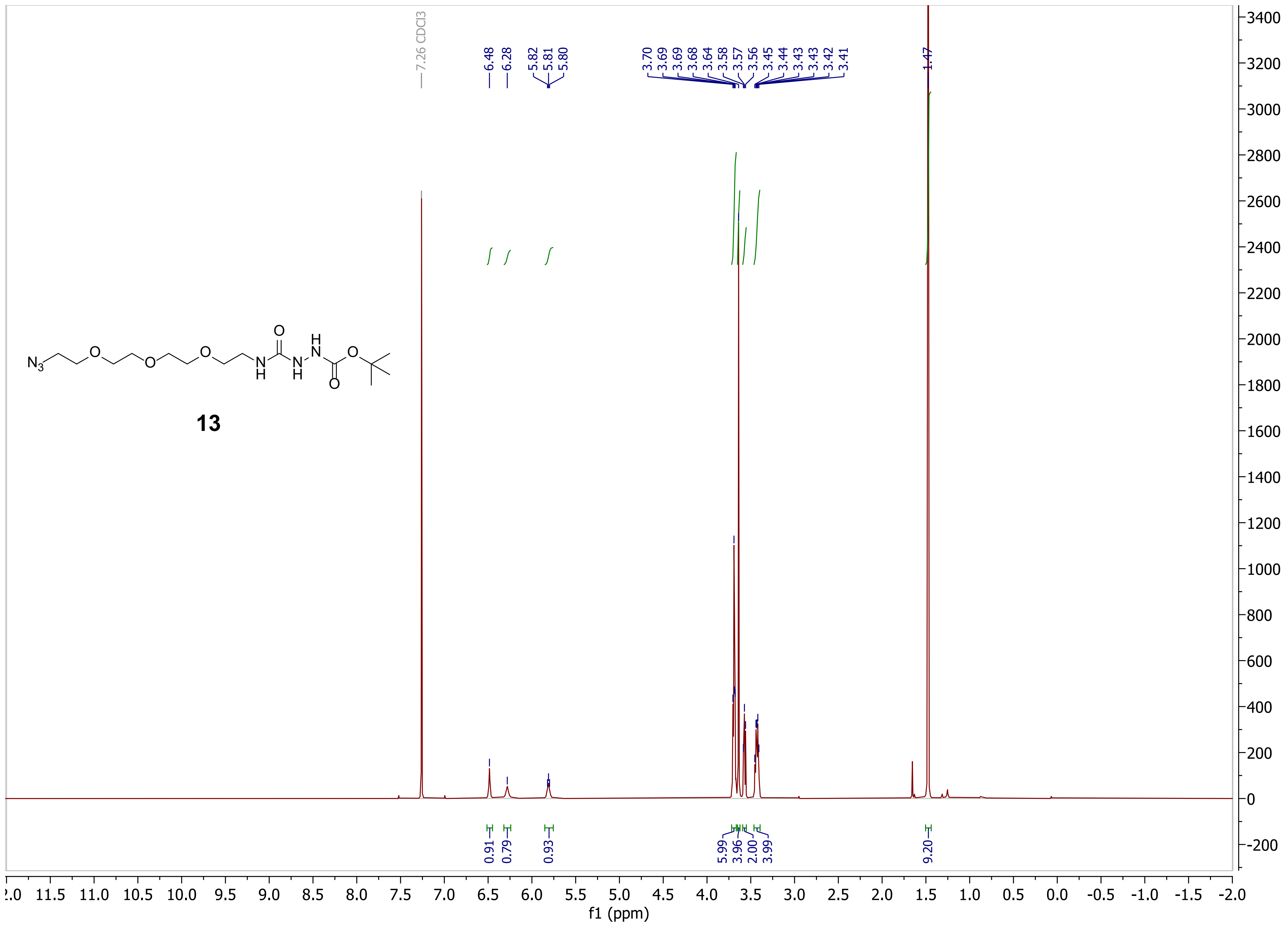
7.26 CDCl3
6.48
6.28
5.82
5.81
5.80
3.70
3.69
3.69
3.68
3.64
3.58
3.57
3.56
3.45
3.44
3.43
3.43
3.42
3.41
1.47
13
0.91
0.79
0.93
5.99
3.96
2.00
3.99
9.20
f1 (ppm)

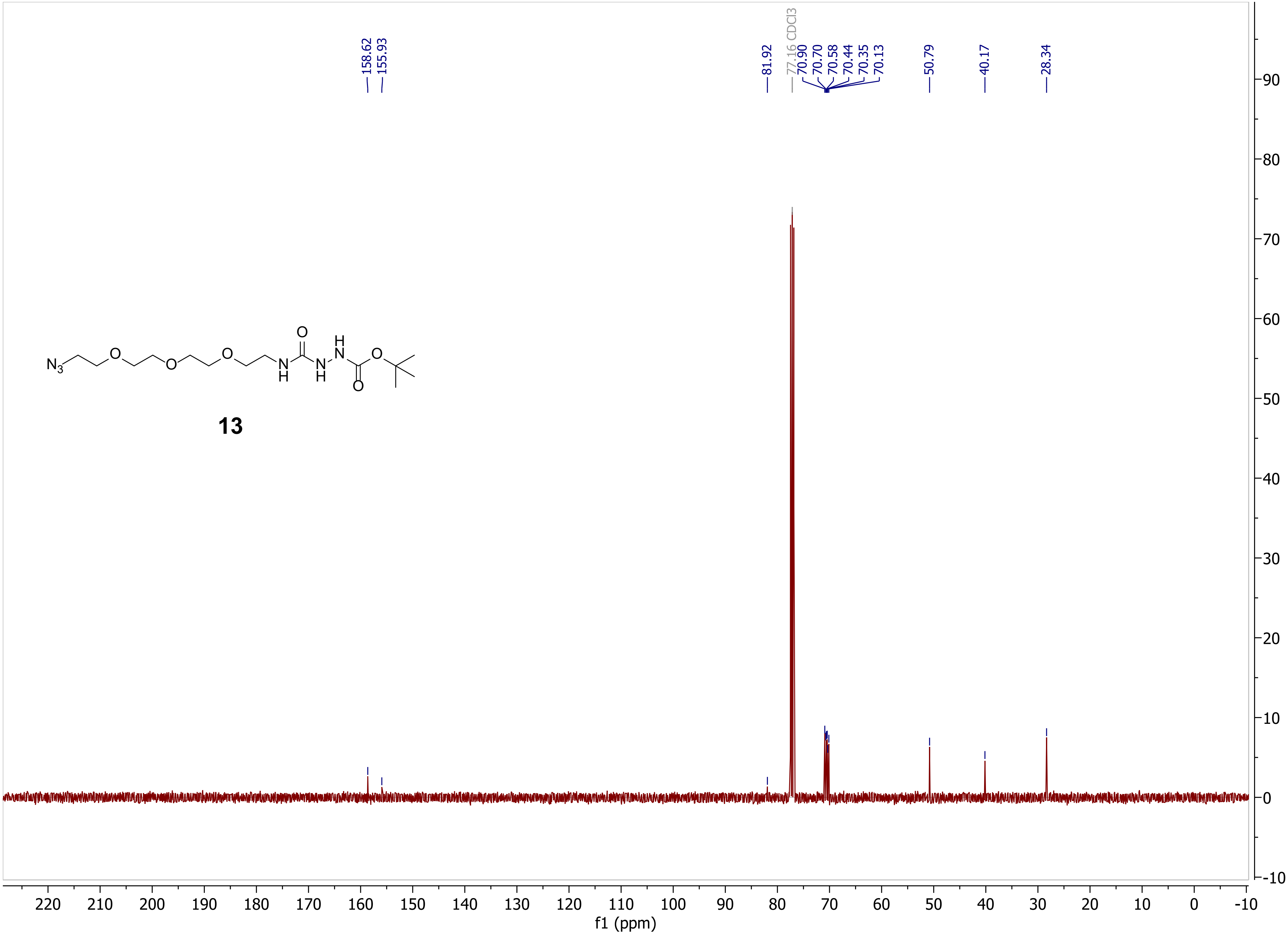
158.62
155.93
81.92
77.16 CDCl3
70.90
70.70
70.58
70.44
70.35
70.13
50.79
40.17
28.34
13
f1 (ppm)

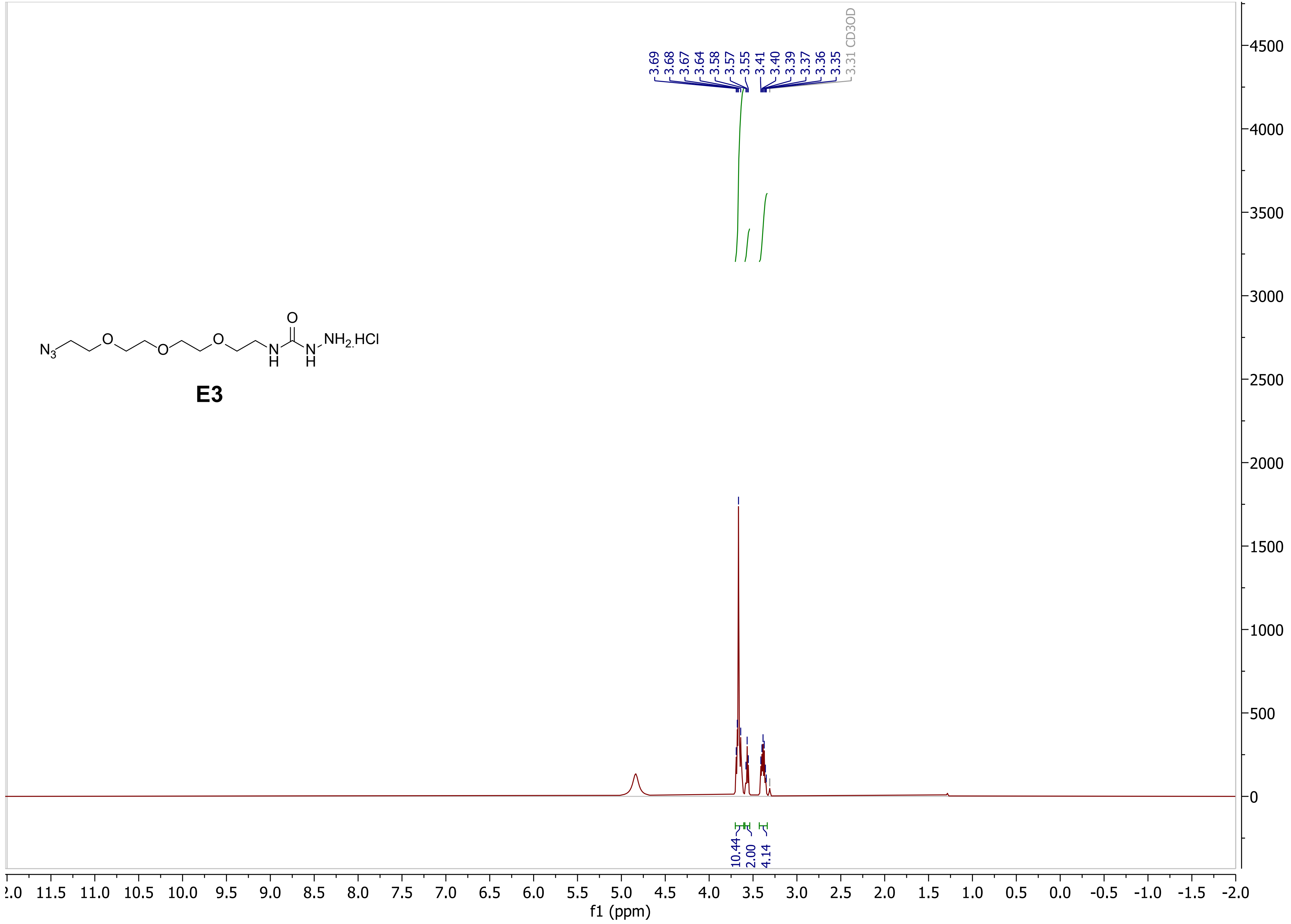
3.69
3.68
3.67
3.64
3.58
3.57
3.55
3.41
3.40
3.39
3.37
3.36
3.35
3.31 CD3OD
N3
O
O
O
N
H
O
N
H
NH2.HCl
E3
10.44
2.00
4.14
f1 (ppm)

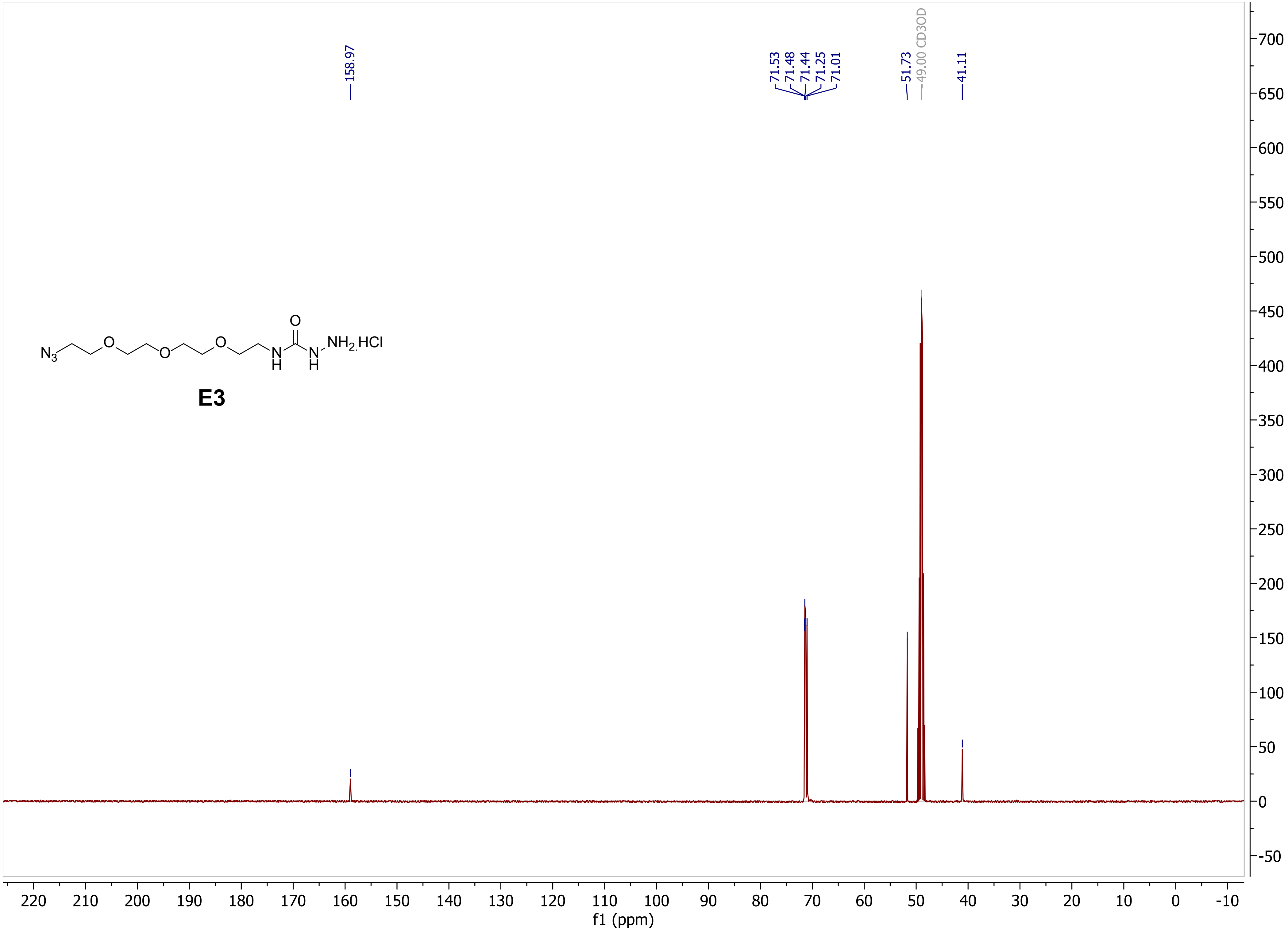
158.97
71.53
71.48
71.44
71.25
71.01
51.73
49.00 CD3OD
41.11
N3
O
O
O
O
N
H
N
H
NH2.HCl
E3
f1 (ppm)

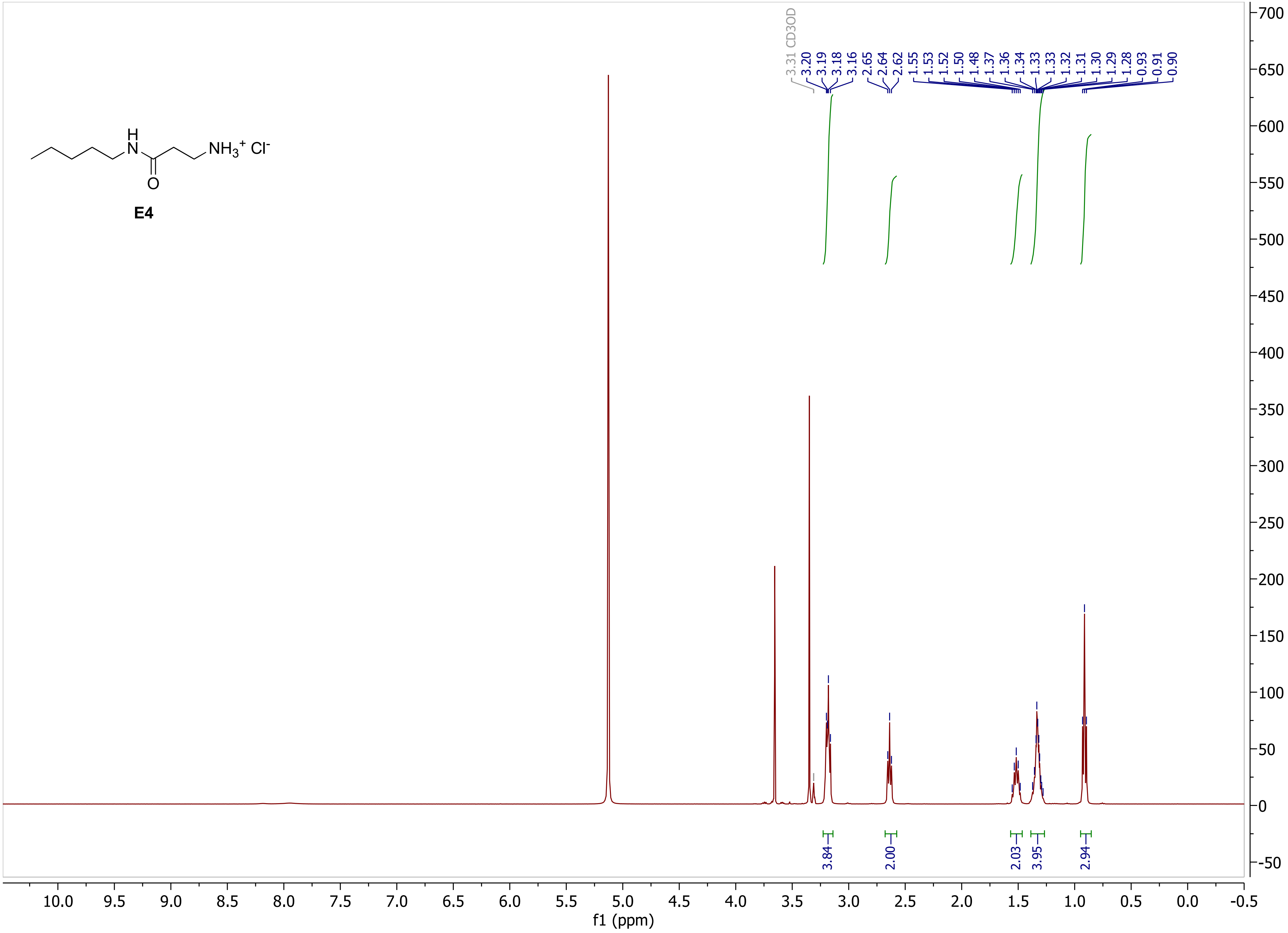

H
N
NH_3^+ Cl$^-$
O
E4
3.31 CD3OD
3.20
3.19
3.18
3.16
2.65
2.64
2.62
1.55
1.53
1.52
1.50
1.48
1.37
1.36
1.34
1.33
1.33
1.32
1.31
1.30
1.29
1.28
0.93
0.91
0.90
3.84
2.00
2.03
3.95
2.94
f1 (ppm)

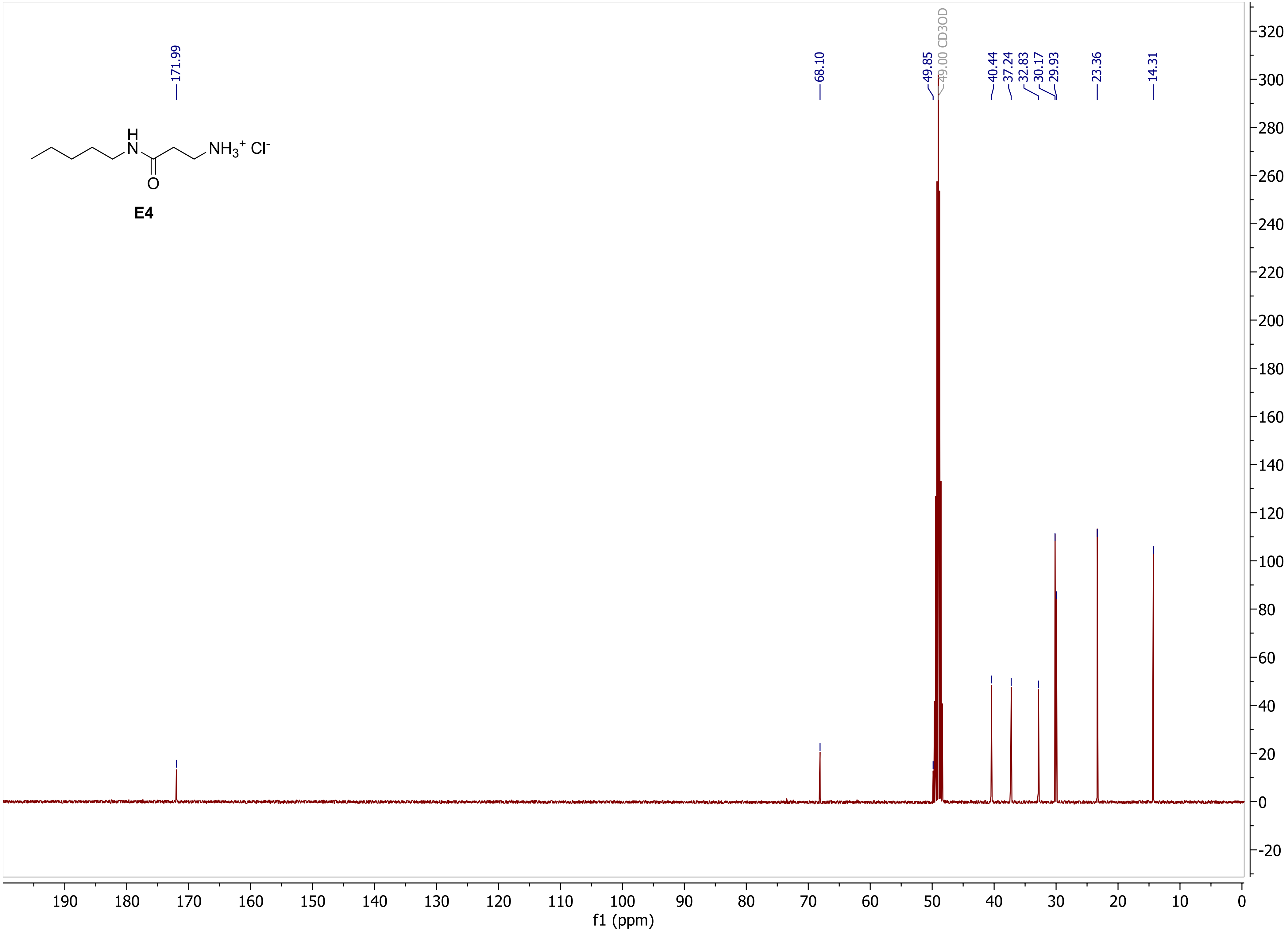

171.99
68.10
49.85
49.00 CD3OD
40.44
37.24
32.83
30.17
29.93
23.36
14.31
H
N
NH3+ Cl-
O
E4
f1 (ppm)

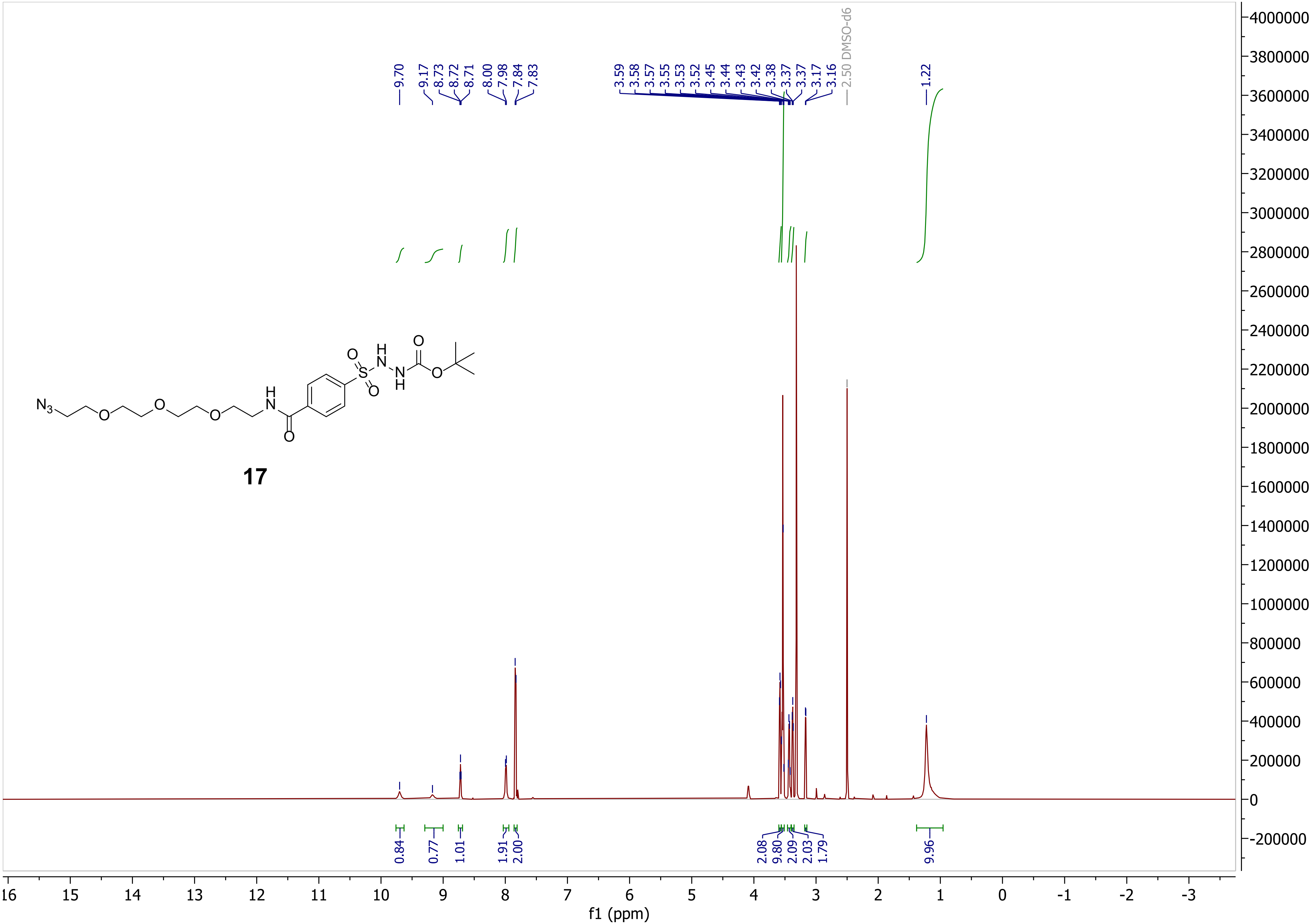

9.70
9.17
8.73
8.72
8.71
8.00
7.98
7.84
7.83
3.59
3.58
3.57
3.55
3.53
3.52
3.45
3.44
3.43
3.42
3.38
3.37
3.37
3.17
3.16
2.50 DMSO-d6
1.22
N3
O
H
N
S
17
0.84
0.77
1.01
1.91
2.00
2.08
9.80
2.09
2.03
1.79
9.96
f1 (ppm)

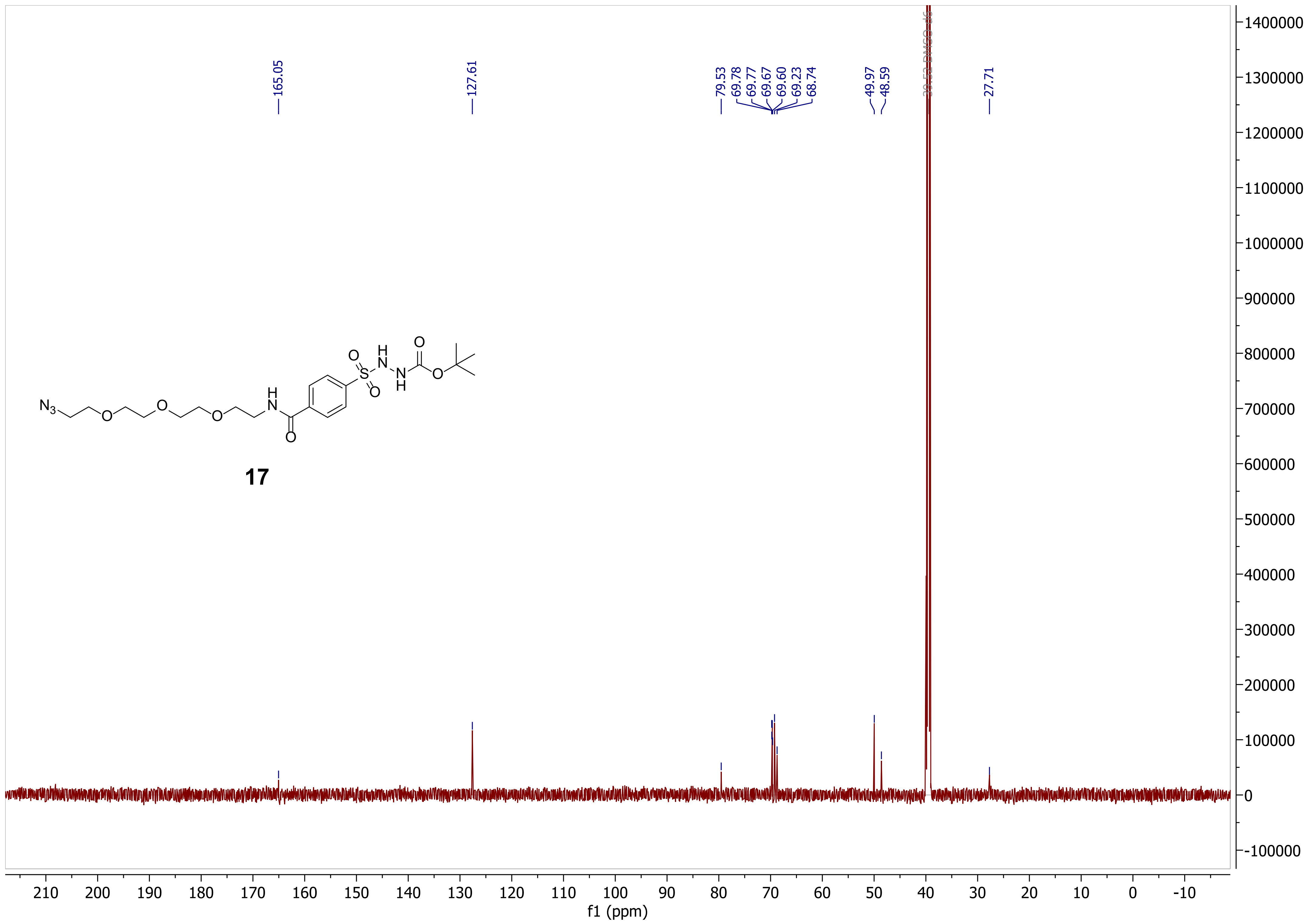

165.05
127.61
79.53
69.78
69.77
69.67
69.60
69.23
68.74
49.97
48.59
39.52 DMSO-d6
27.71
17
f1 (ppm)

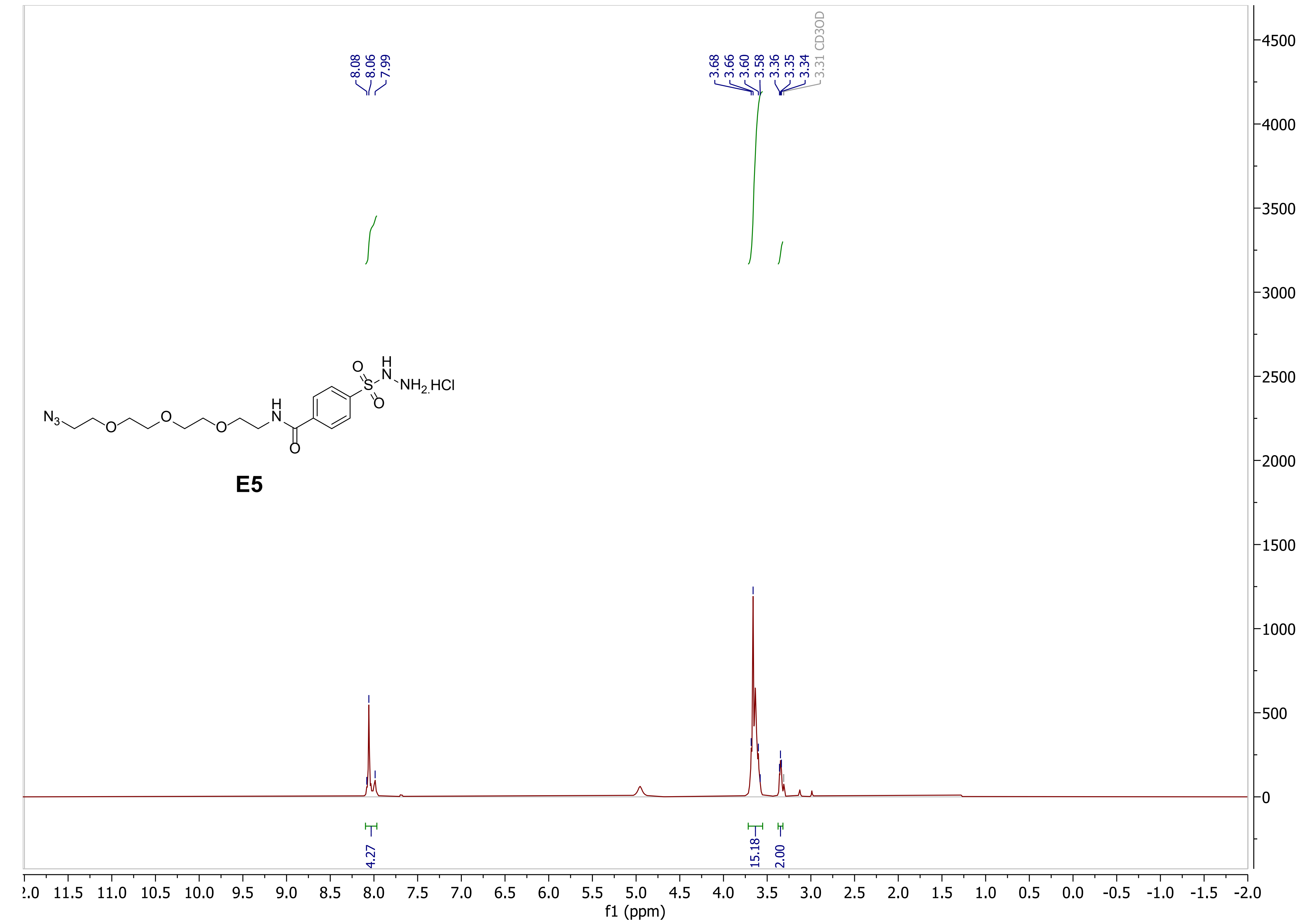
8.08
8.06
7.99
3.68
3.66
3.60
3.58
3.36
3.35
3.34
3.31 CD3OD
4.27
15.18
2.00
E5
N3
O
H
N
S
NH2.HCl
4500
4000
3500
3000
2500
2000
1500
1000
500
0
f1 (ppm)

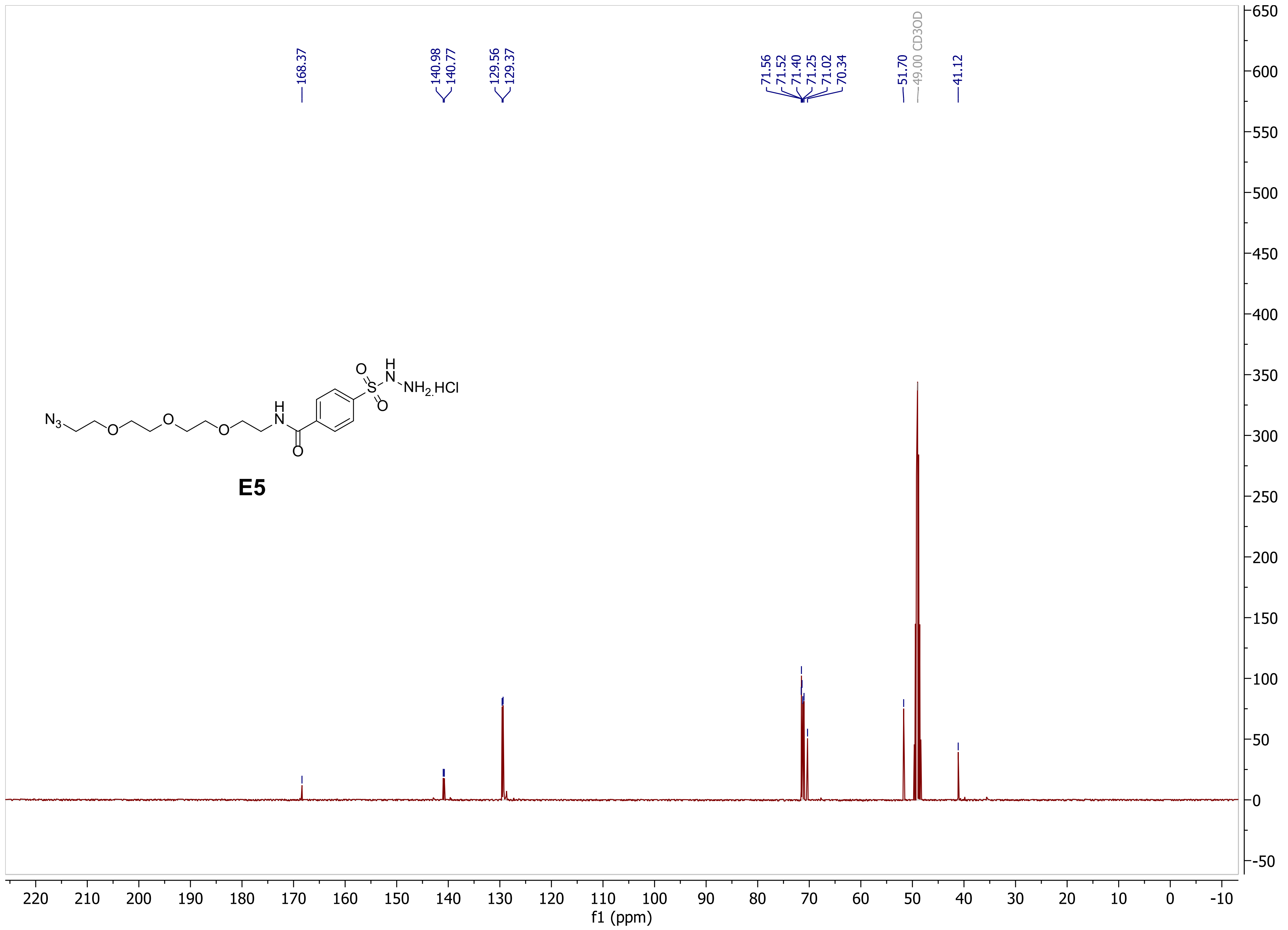

168.37
140.98
140.77
129.56
129.37
71.56
71.52
71.40
71.25
71.02
70.34
51.70
49.00 CD3OD
41.12
N3
O
H
N
S
NH2.HCl
E5
f1 (ppm)

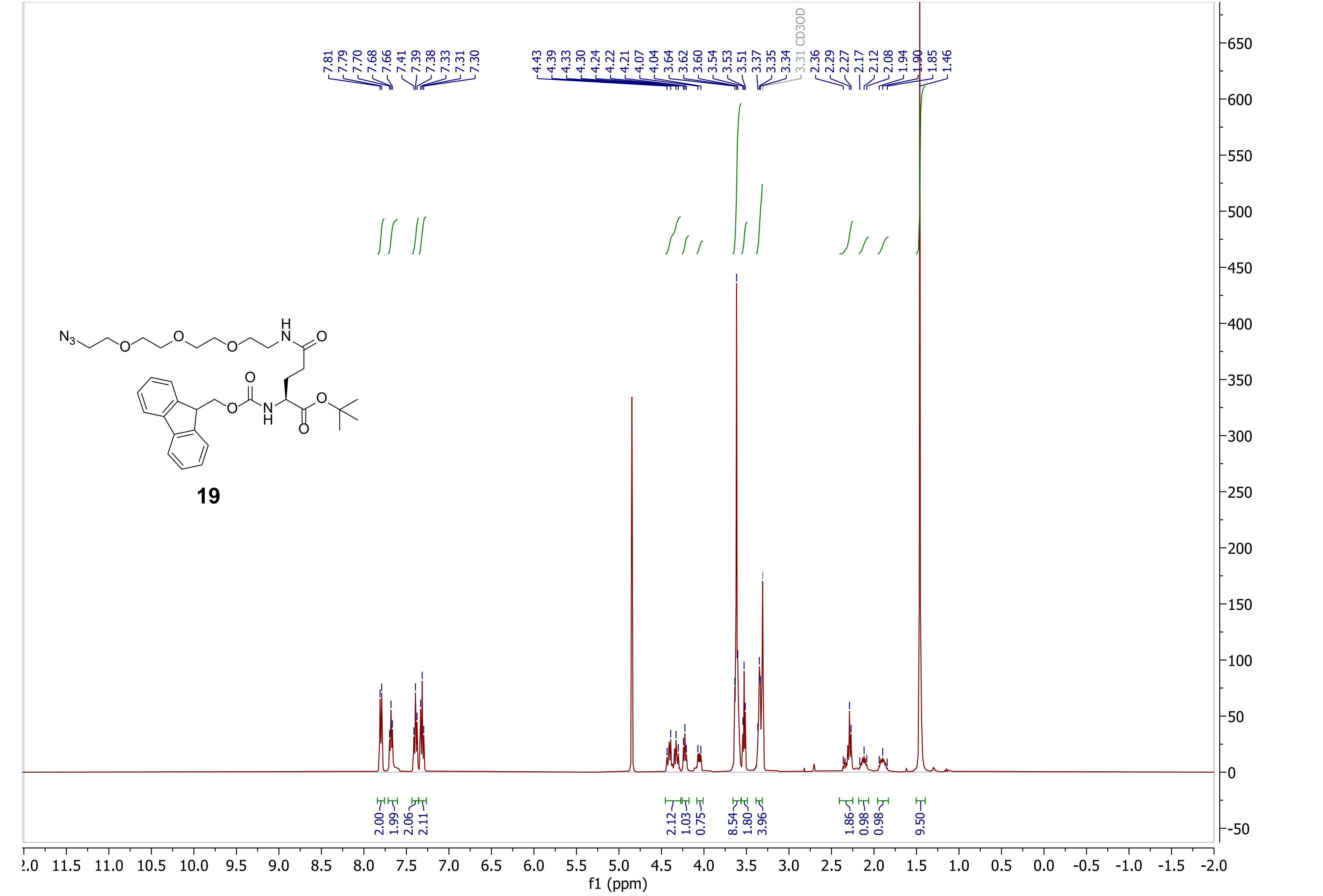
7.81
7.79
7.70
7.68
7.66
7.41
7.39
7.38
7.33
7.31
7.30
4.43
4.39
4.33
4.30
4.24
4.22
4.21
4.07
4.04
3.64
3.62
3.60
3.54
3.53
3.51
3.37
3.35
3.34
3.31 CD3OD
2.36
2.29
2.27
2.17
2.12
2.08
1.94
1.90
1.85
1.46
2.00
1.99
2.06
2.11
2.12
1.03
0.75
8.54
1.80
3.96
1.86
0.98
0.98
9.50
N3
O
H
N
19
f1 (ppm)

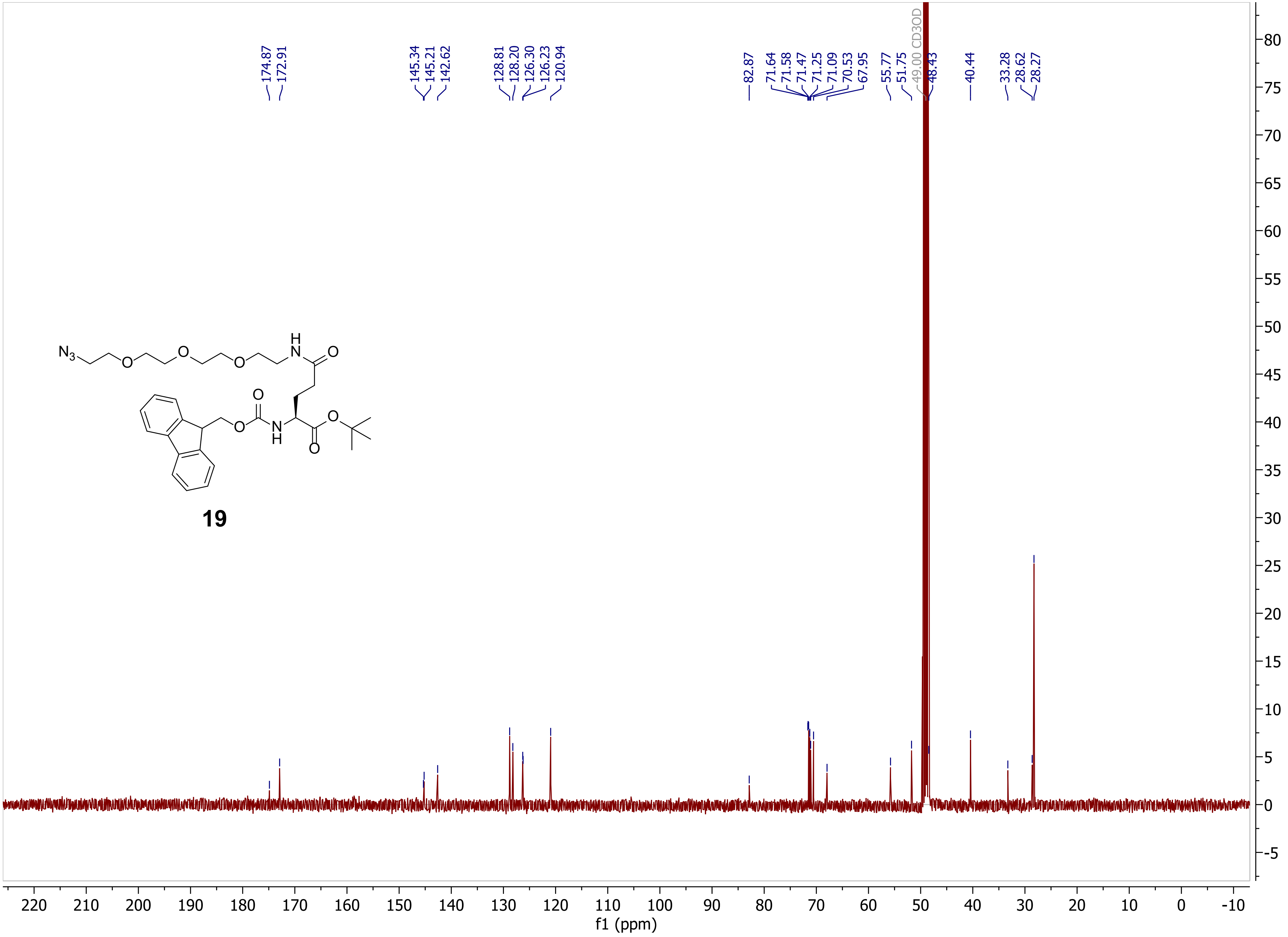

174.87
172.91
145.34
145.21
142.62
128.81
128.20
126.30
126.23
120.94
82.87
71.64
71.58
71.47
71.25
71.09
70.53
67.95
55.77
51.75
49.00 CD3OD
48.43
40.44
33.28
28.62
28.27
N3
O
H
N
19
f1 (ppm)

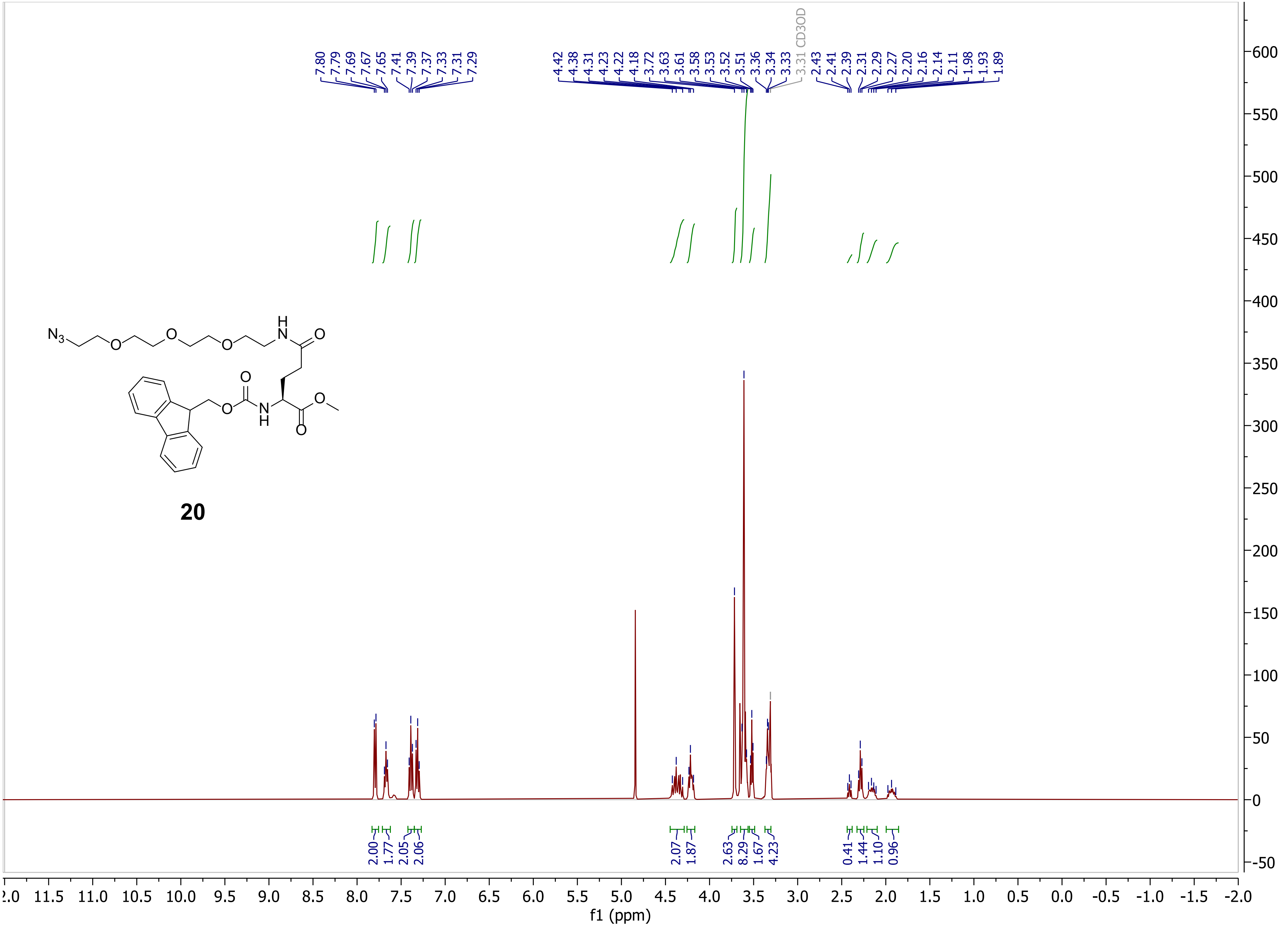
7.80 7.79 7.69 7.67 7.65 7.41 7.39 7.37 7.33 7.31 7.29
4.42 4.38 4.31 4.23 4.22 4.18 3.72 3.63 3.61 3.58 3.53 3.52 3.51 3.36 3.34 3.33
3.31 CD3OD
2.43 2.41 2.39 2.31 2.29 2.27 2.20 2.16 2.14 2.11 1.98 1.93 1.89
2.00 1.77 2.05 2.06
2.07 1.87
2.63 8.29 1.67 4.23
0.41 1.44 1.10 0.96
20
f1 (ppm)

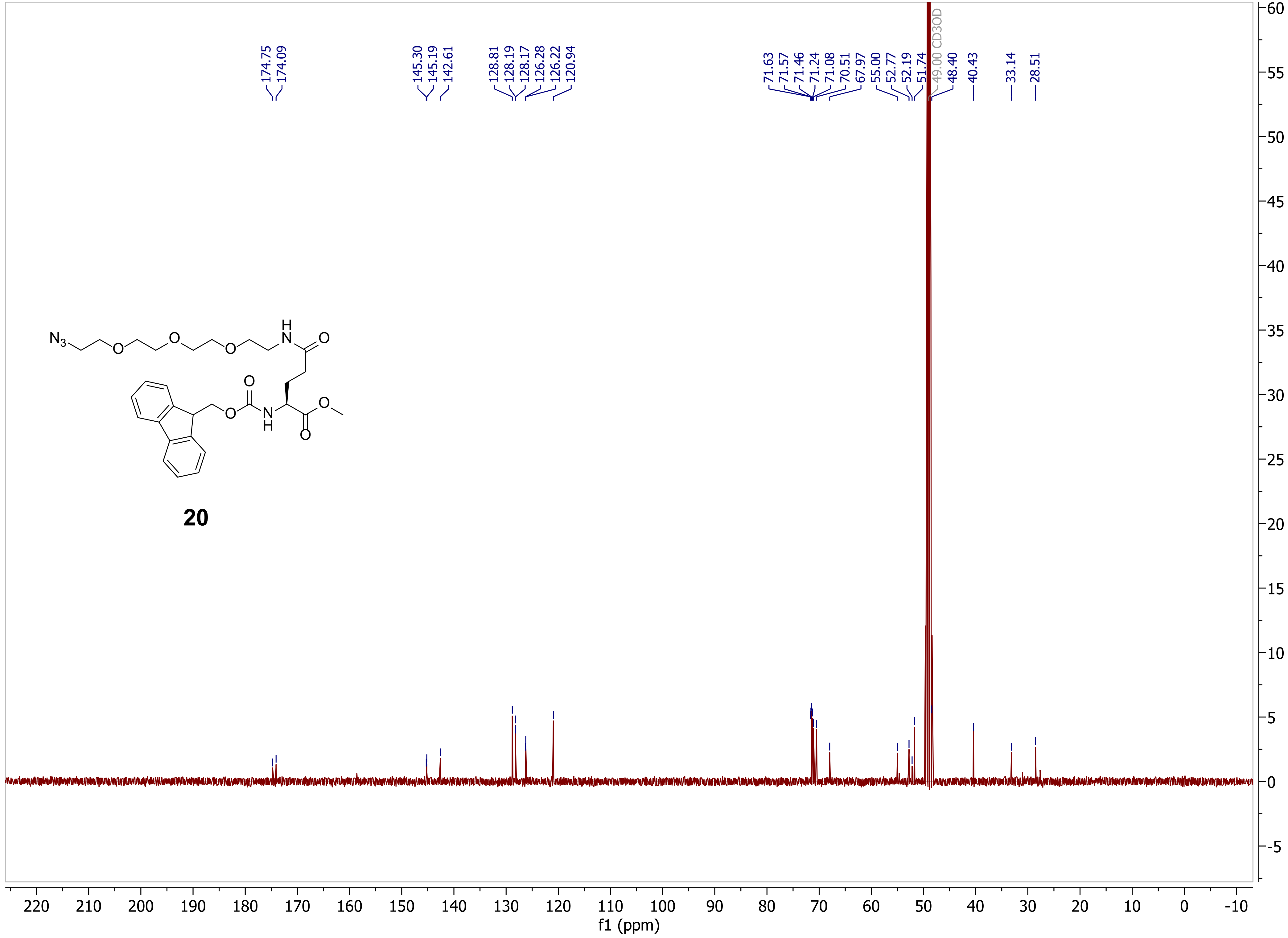
174.75
174.09
145.30
145.19
142.61
128.81
128.19
128.17
126.28
126.22
120.94
71.63
71.57
71.46
71.24
71.08
70.51
67.97
55.00
52.77
52.19
51.74
49.00 CD3OD
48.40
40.43
33.14
28.51
20
f1 (ppm)

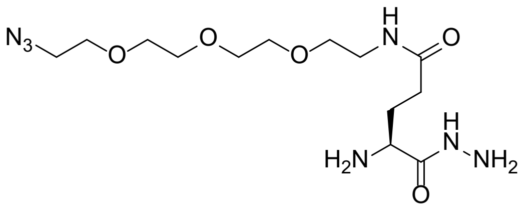

E6

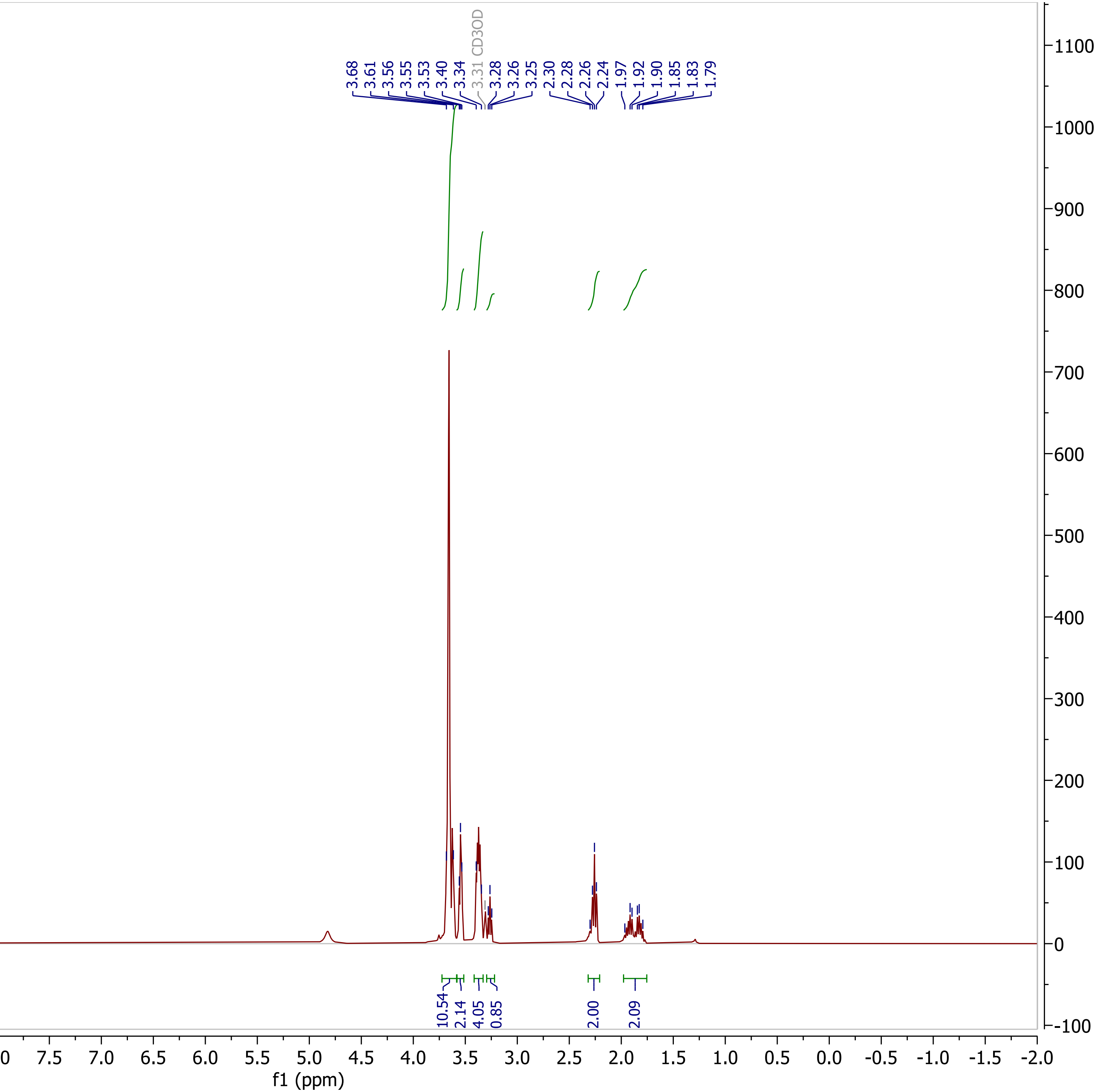

3.68
3.61
3.56
3.55
3.53
3.40
3.34
3.31 CD3OD
3.28
3.26
3.25
2.30
2.28
2.26
2.24
1.97
1.92
1.90
1.85
1.83
1.79
10.54
2.14
4.05
0.85
2.00
2.09
f1 (ppm)

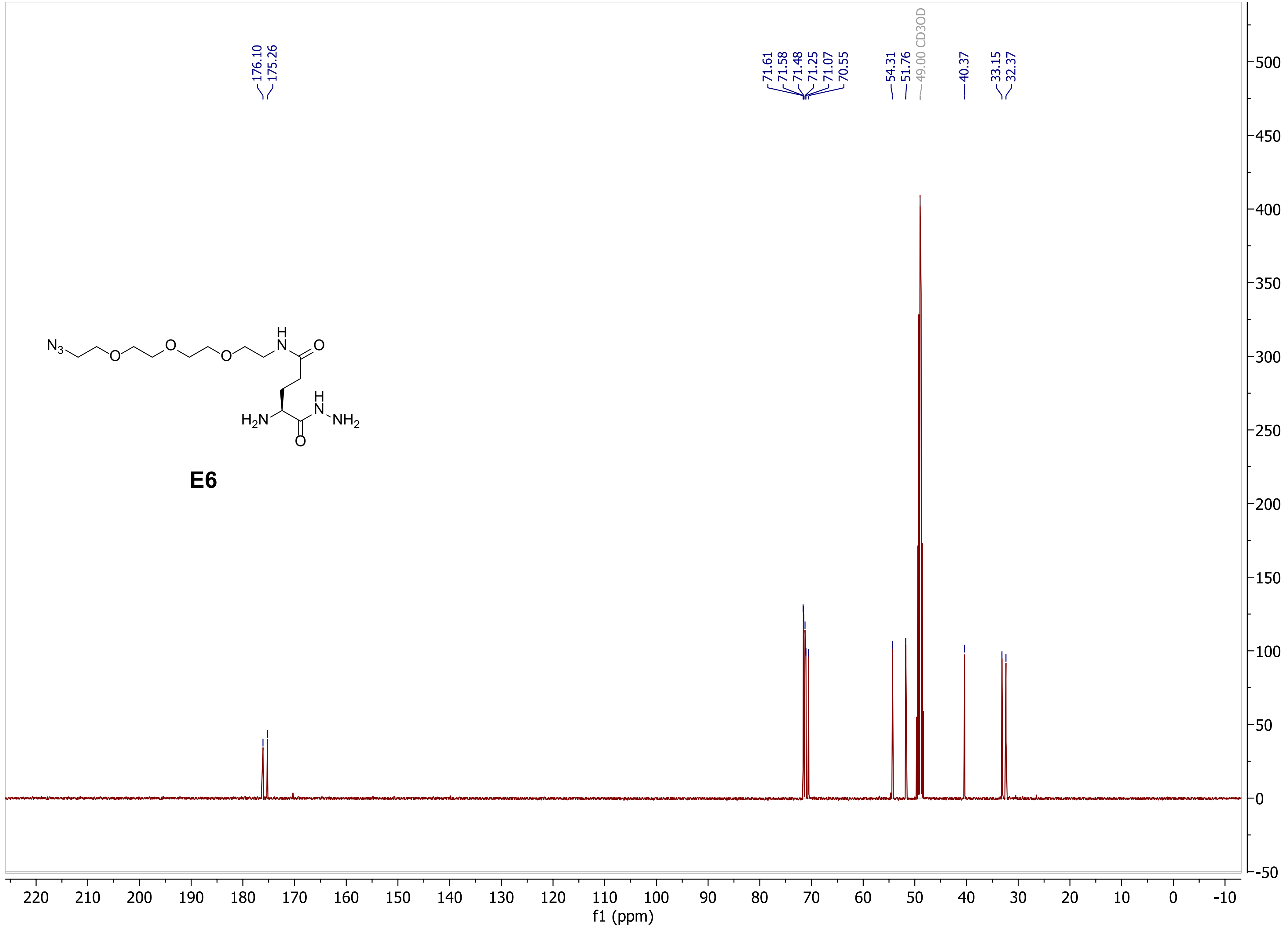
176.10
175.26
71.61
71.58
71.48
71.25
71.07
70.55
54.31
51.76
49.00 CD3OD
40.37
33.15
32.37
N3
O
O
O
H
N
O
H2N
H
N
NH2
O
E6
f1 (ppm)
220
210
200
190
180
170
160
150
140
130
120
110
100
90
80
70
60
50
40
30
20
10
0
-10
500
450
400
350
300
250
200
150
100
50
0
-50

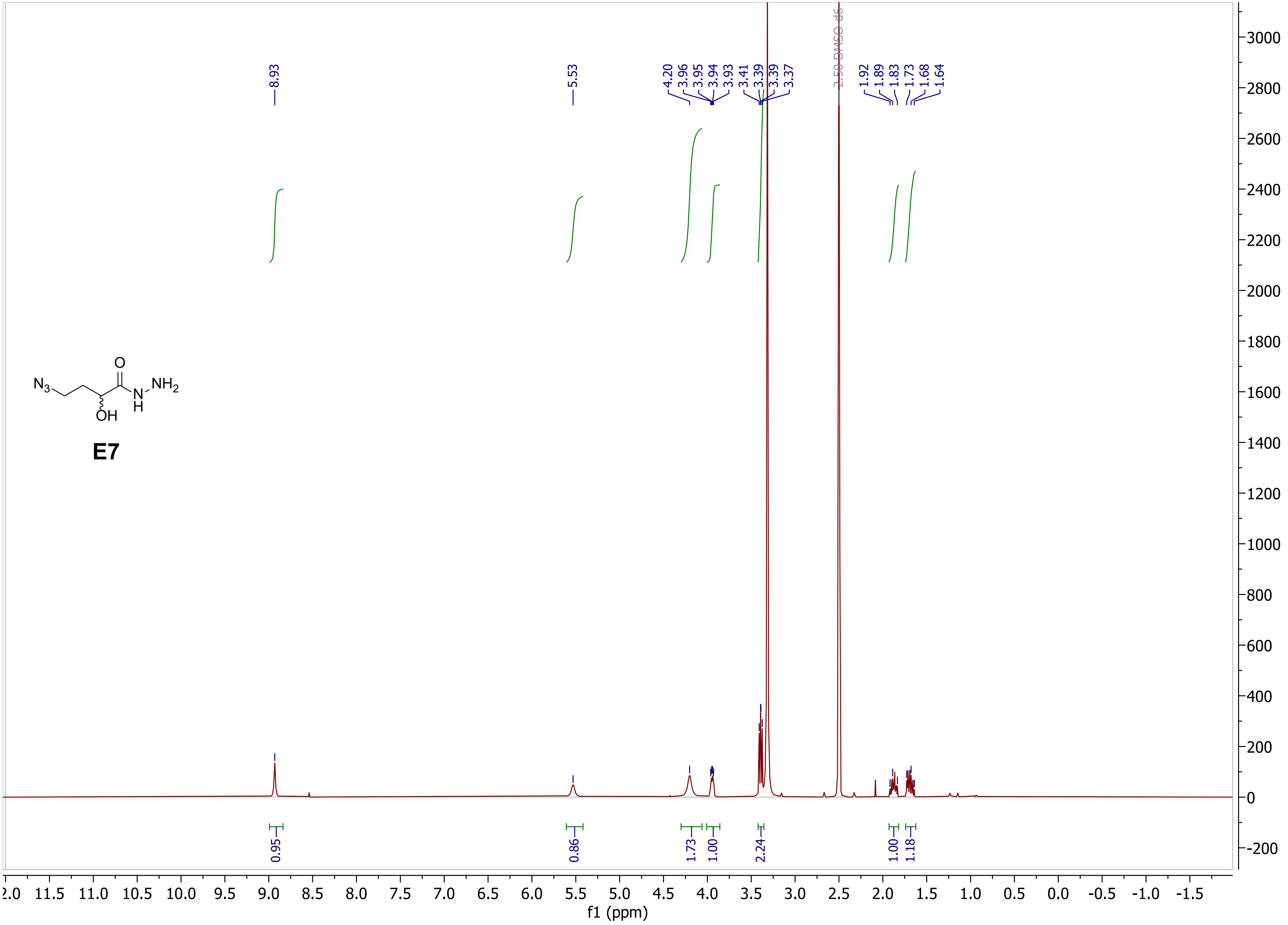
8.93
5.53
4.20
3.96
3.95
3.94
3.93
3.41
3.39
3.39
3.37
2.50 DMSO-d6
1.92
1.89
1.83
1.73
1.68
1.64
0.95
0.86
1.73
1.00
2.24
1.00
1.18
f1 (ppm)
N3
O
NH2
N
H
OH

E7

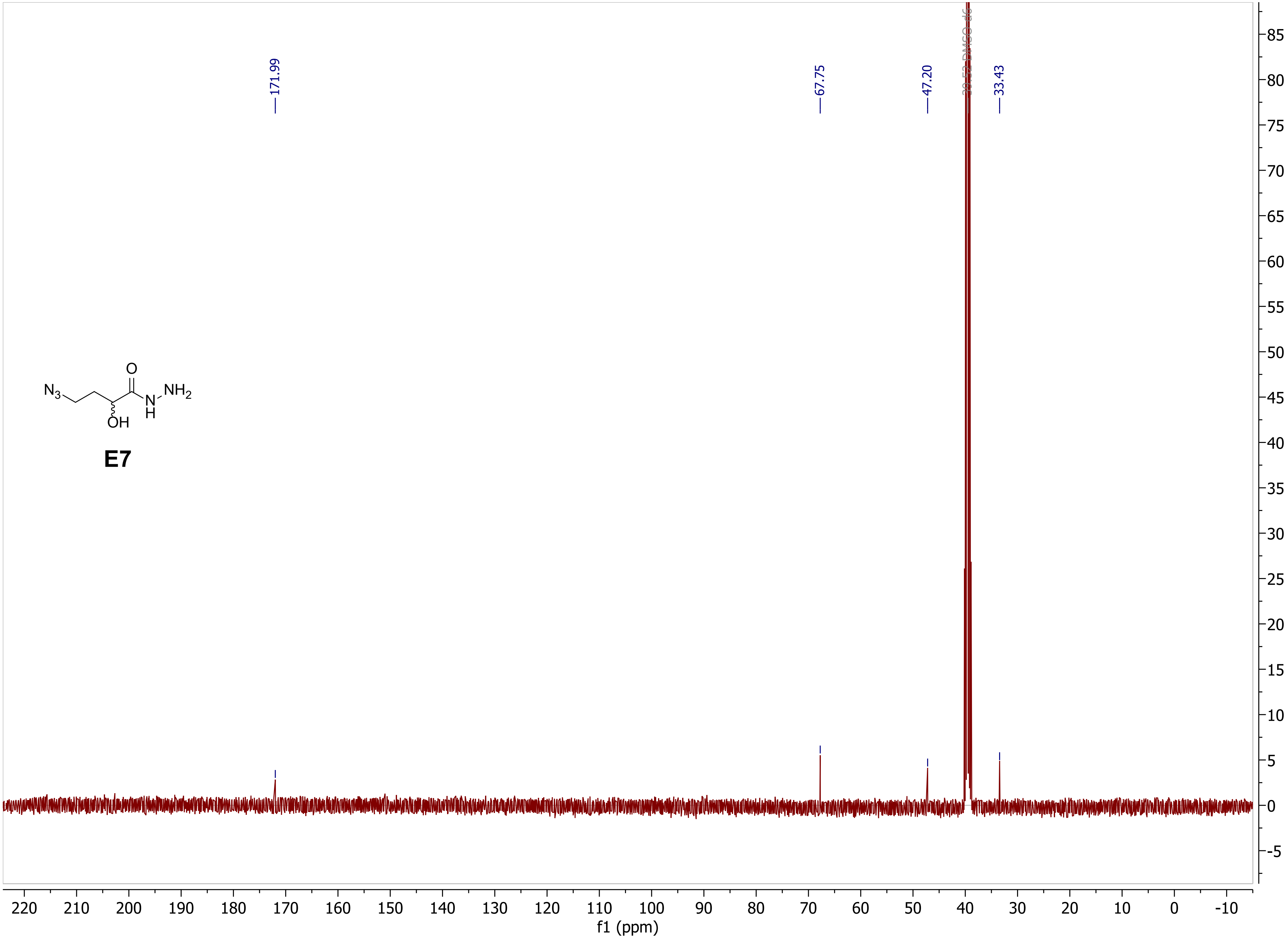
171.99
67.75
47.20
39.52 DMSO-d6
33.43
N3
O
NH2
N
H
OH
E7
220
210
200
190
180
170
160
150
140
130
120
110
100
90
80
70
60
50
40
30
20
10
0
-10
f1 (ppm)
85
80
75
70
65
60
55
50
45
40
35
30
25
20
15
10
5
0
-5

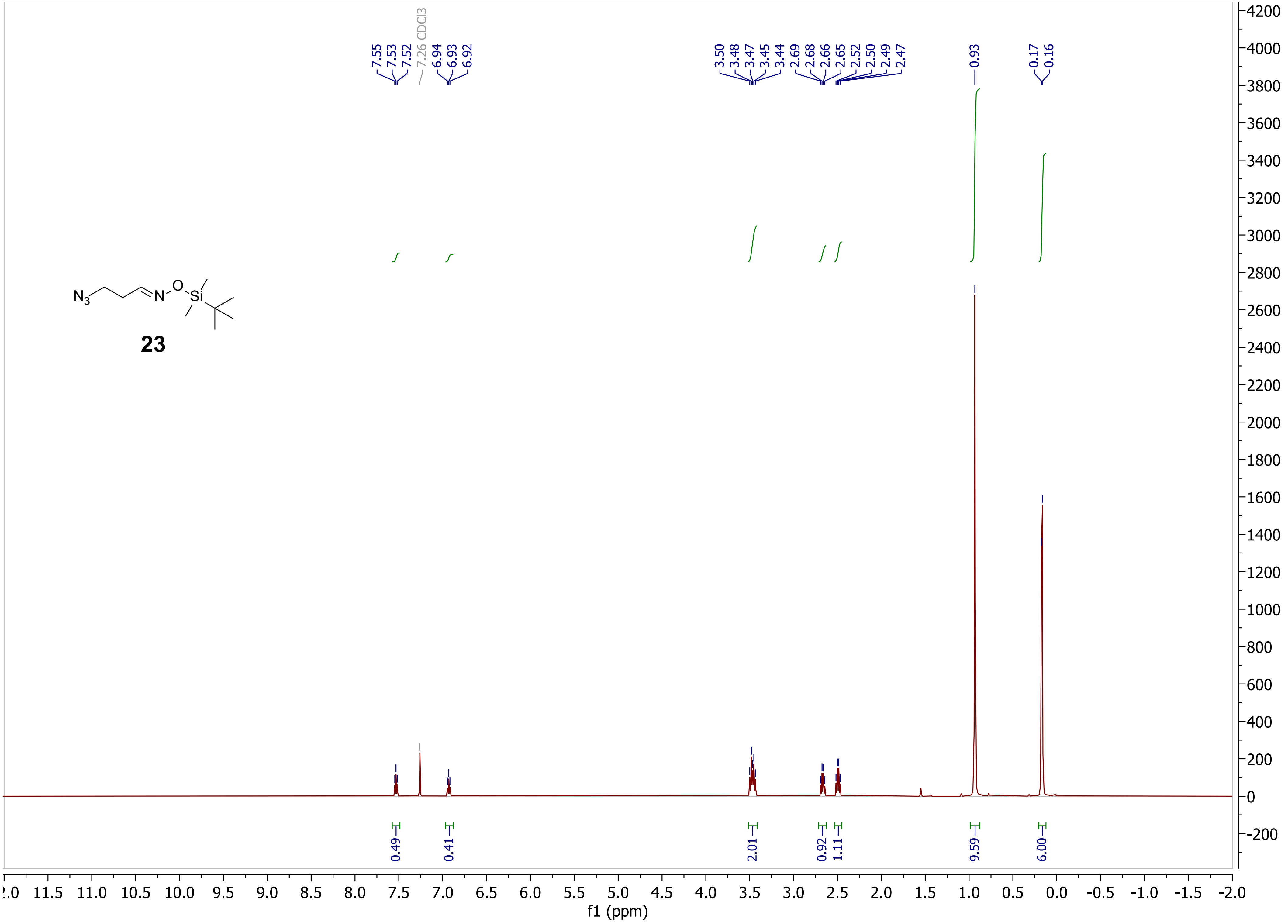

7.55
7.53
7.52
7.26 CDCl3
6.94
6.93
6.92
3.50
3.48
3.47
3.45
3.44
2.69
2.68
2.66
2.65
2.52
2.50
2.49
2.47
0.93
0.17
0.16
N_3
N
O
Si
23
0.49
0.41
2.01
0.92
1.11
9.59
6.00
f1 (ppm)

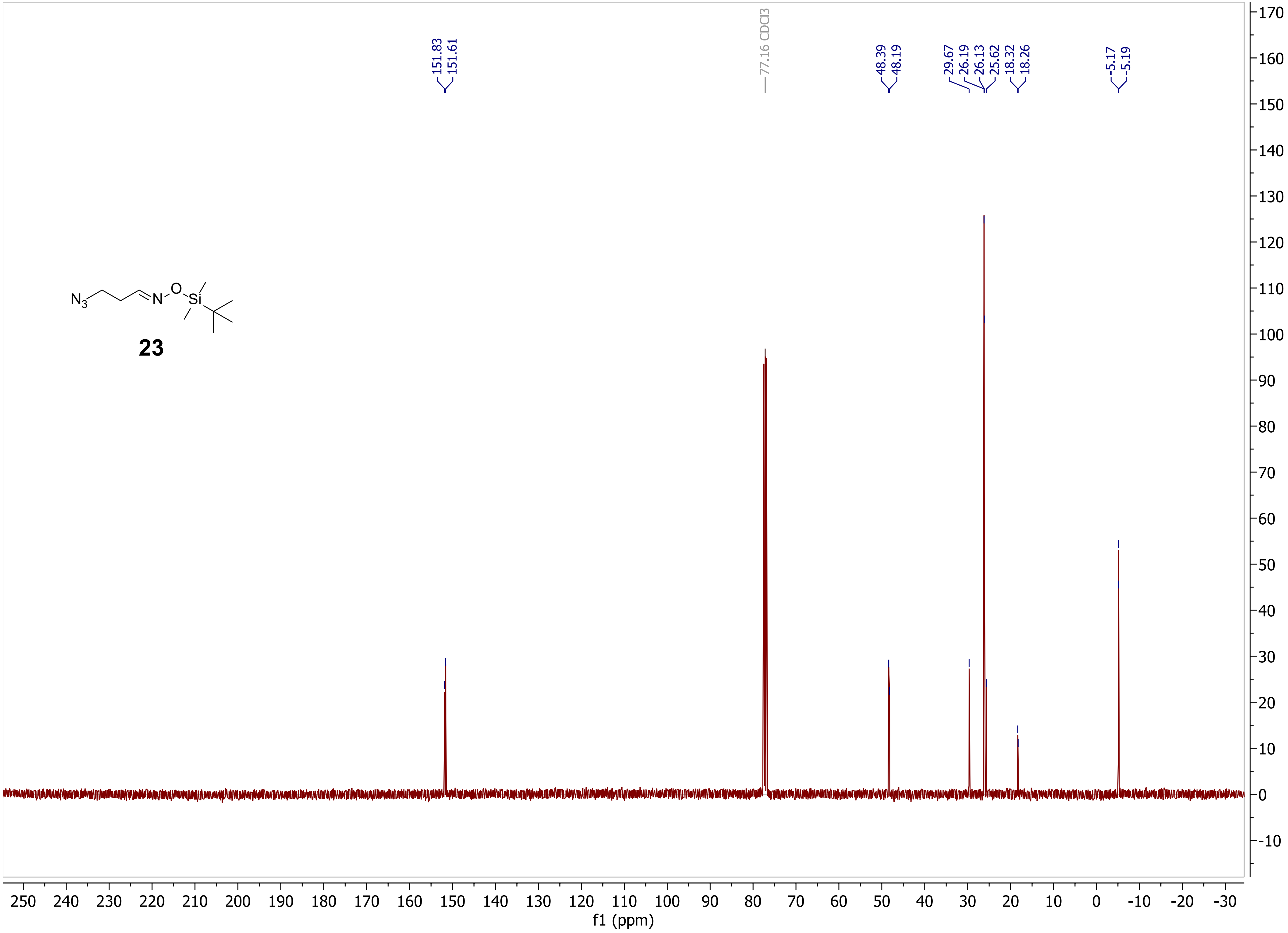

151.83
151.61
77.16 CDCl3
48.39
48.19
29.67
26.19
26.13
25.62
18.32
18.26
-5.17
-5.19
N3
N
O
Si
23
f1 (ppm)

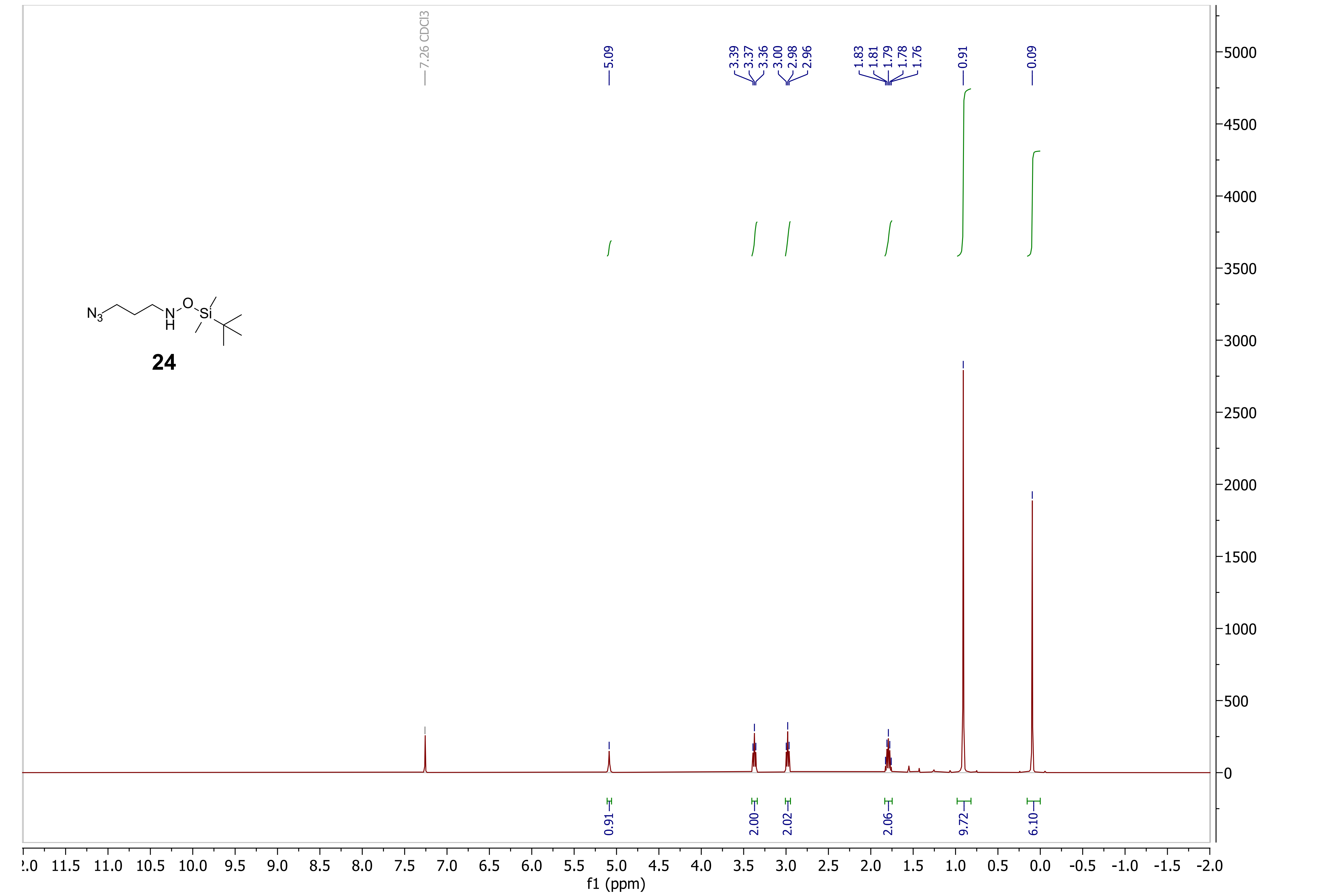
7.26 CDCl3
5.09
3.39
3.37
3.36
3.00
2.98
2.96
1.83
1.81
1.79
1.78
1.76
0.91
0.09
24
0.91
2.00
2.02
2.06
9.72
6.10
f1 (ppm)

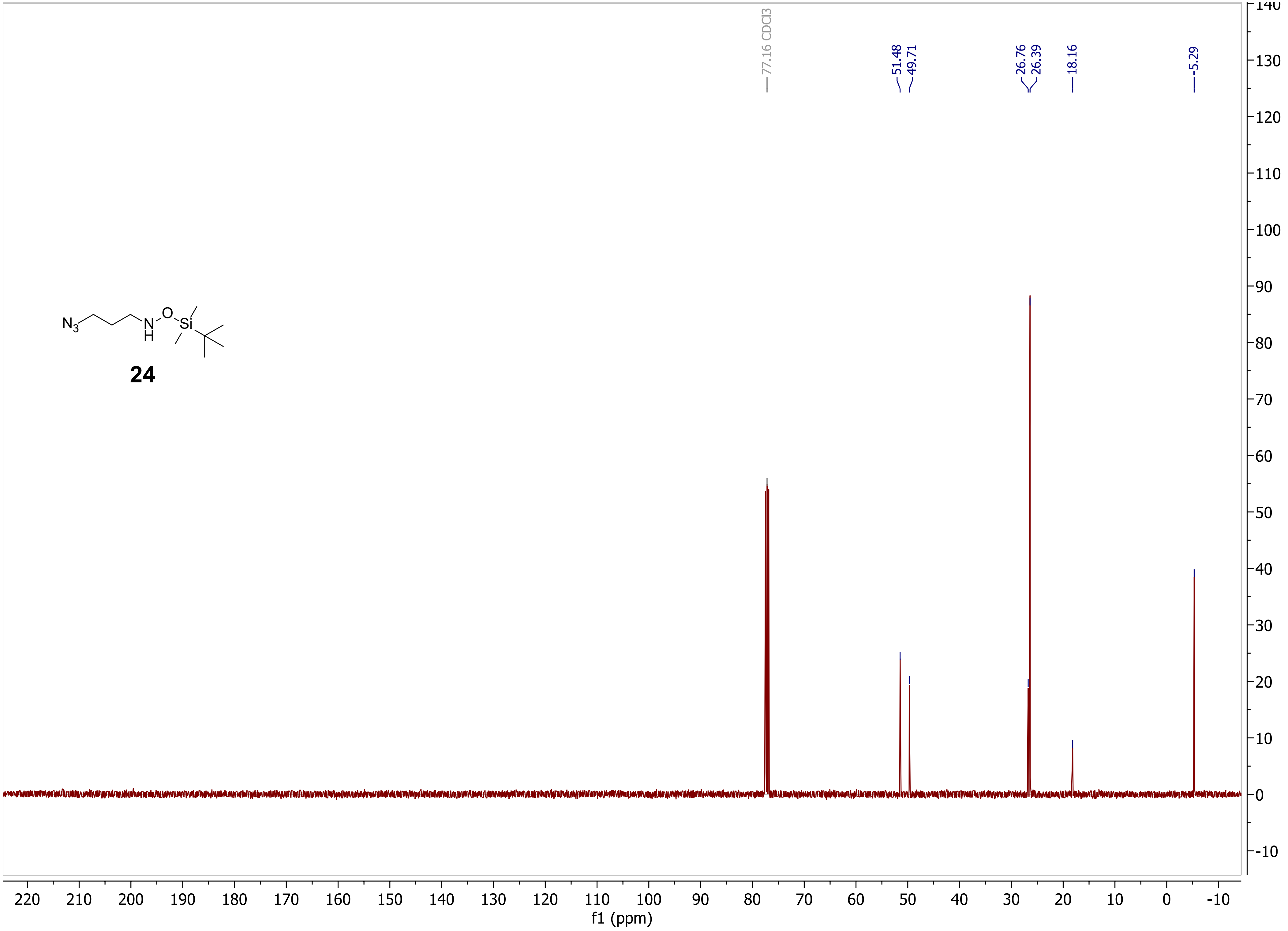
77.16 CDCl3
51.48
49.71
26.76
26.39
18.16
-5.29
N3
N
H
O
Si
24
f1 (ppm)

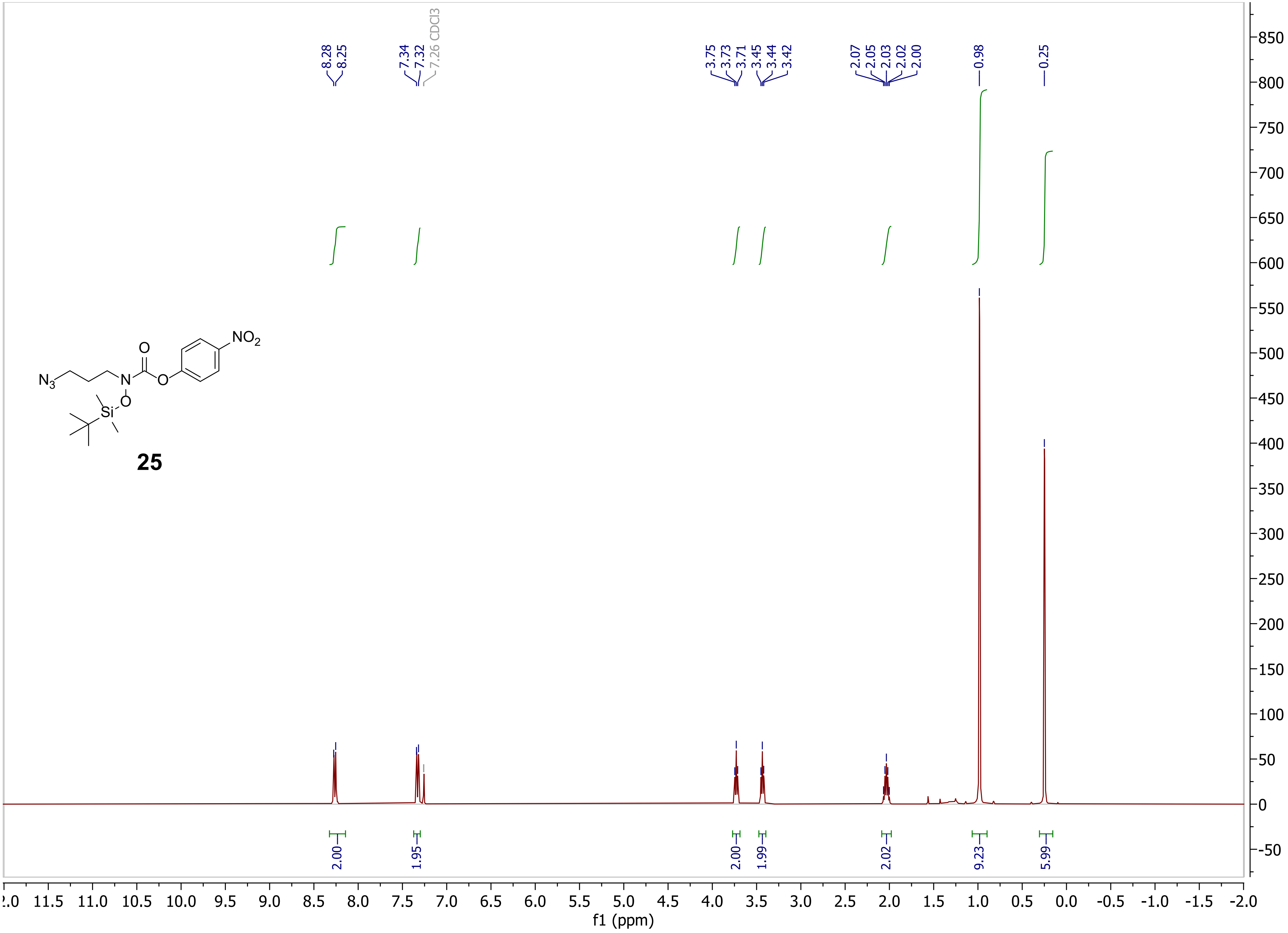
8.28
8.25
7.34
7.32
7.26 CDCl3
3.75
3.73
3.71
3.45
3.44
3.42
2.07
2.05
2.03
2.02
2.00
0.98
0.25
NO2
O
N3
N
O
O
Si
25
2.00
1.95
2.00
1.99
2.02
9.23
5.99
f1 (ppm)

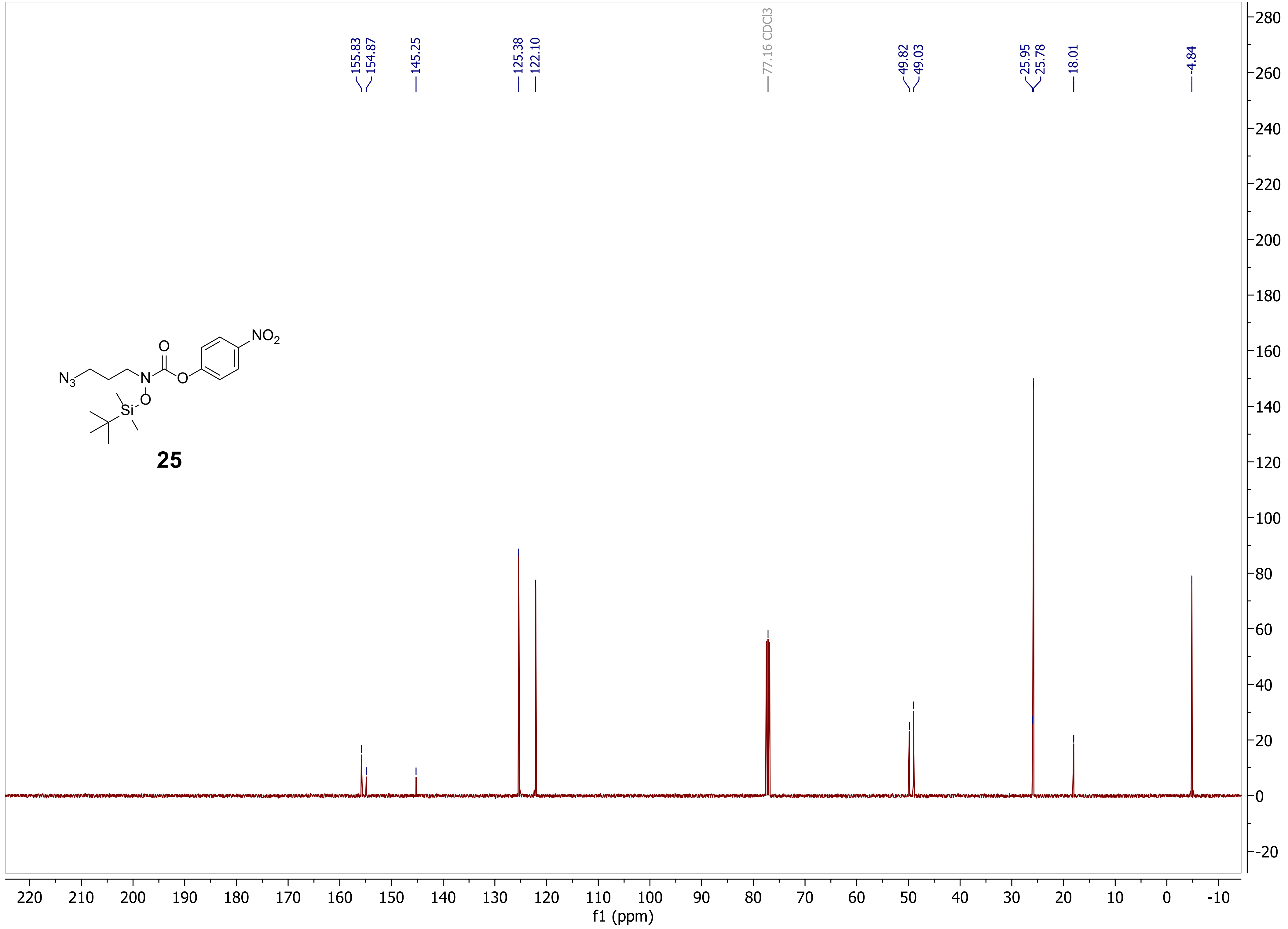
155.83
154.87
145.25
125.38
122.10
77.16 CDCl3
49.82
49.03
25.95
25.78
18.01
-4.84
NO2
O
N3
N
O
O
Si
25
220 210 200 190 180 170 160 150 140 130 120 110 100 90 80 70 60 50 40 30 20 10 0 -10
f1 (ppm)
280 260 240 220 200 180 160 140 120 100 80 60 40 20 0 -20

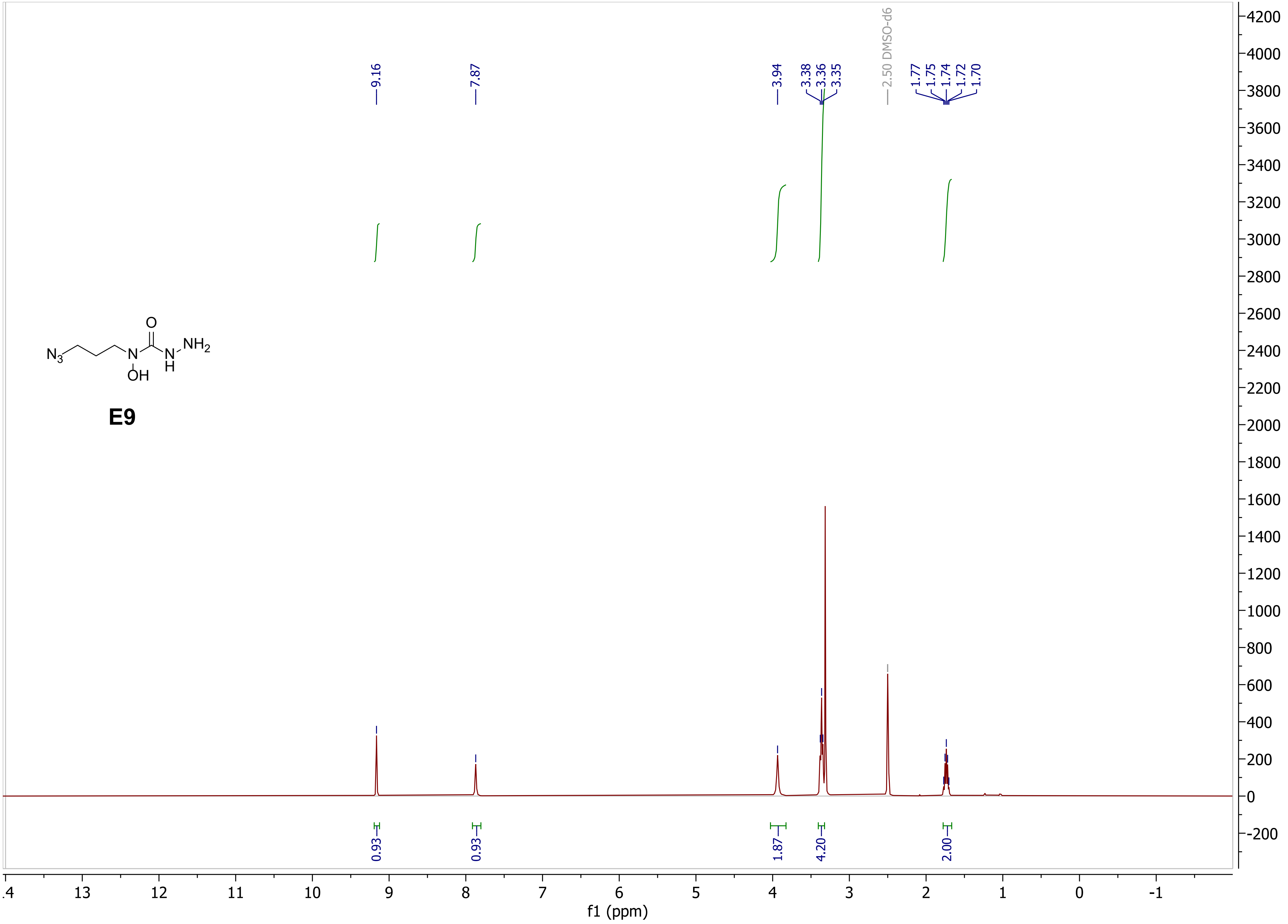

9.16
7.87
3.94
3.38
3.36
3.35
2.50 DMSO-d6
1.77
1.75
1.74
1.72
1.70
0.93
0.93
1.87
4.20
2.00
f1 (ppm)

E9

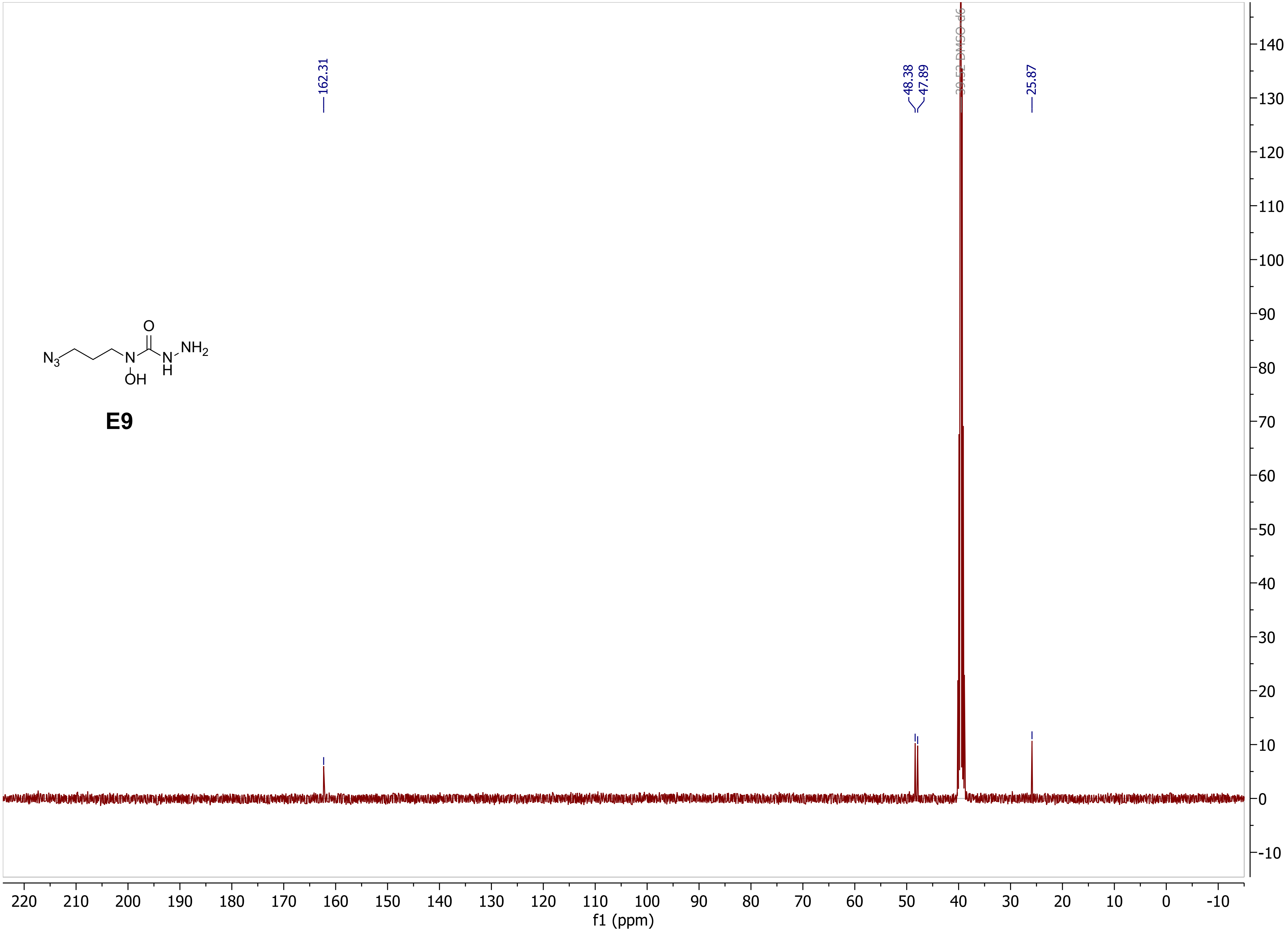

E9
162.31
48.38
47.89
39.52 DMSO-d6
25.87
f1 (ppm)

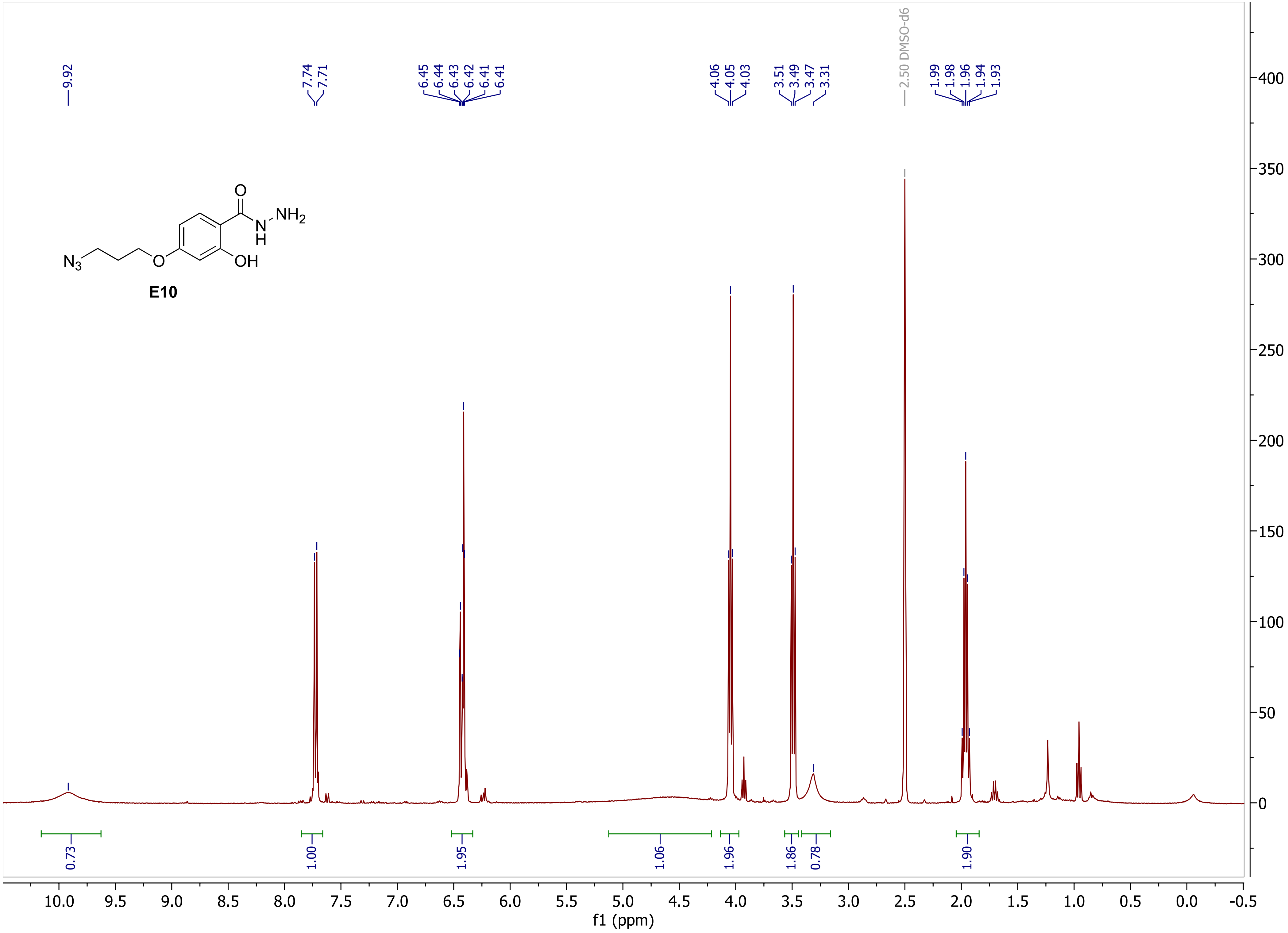

9.92
7.74
7.71
6.45
6.44
6.43
6.42
6.41
6.41
4.06
4.05
4.03
3.51
3.49
3.47
3.31
2.50 DMSO-d6
1.99
1.98
1.96
1.94
1.93
O
NH2
N
H
N3
O
OH
E10
0.73
1.00
1.95
1.06
1.96
1.86
0.78
1.90
f1 (ppm)

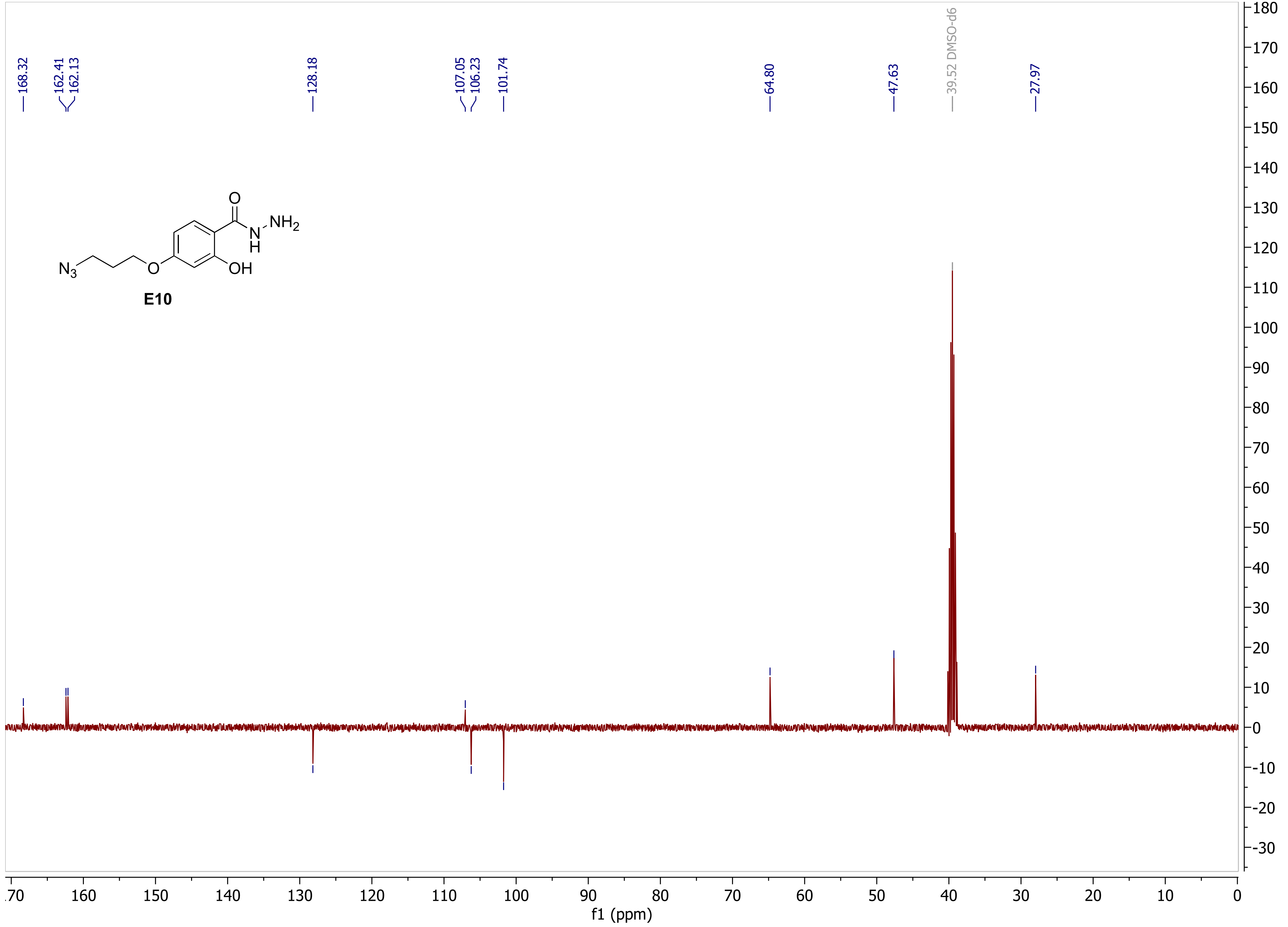

168.32
162.41
162.13
128.18
107.05
106.23
101.74
64.80
47.63
39.52 DMSO-d6
27.97
O
NH2
N
H
N3
O
OH
E10
f1 (ppm)

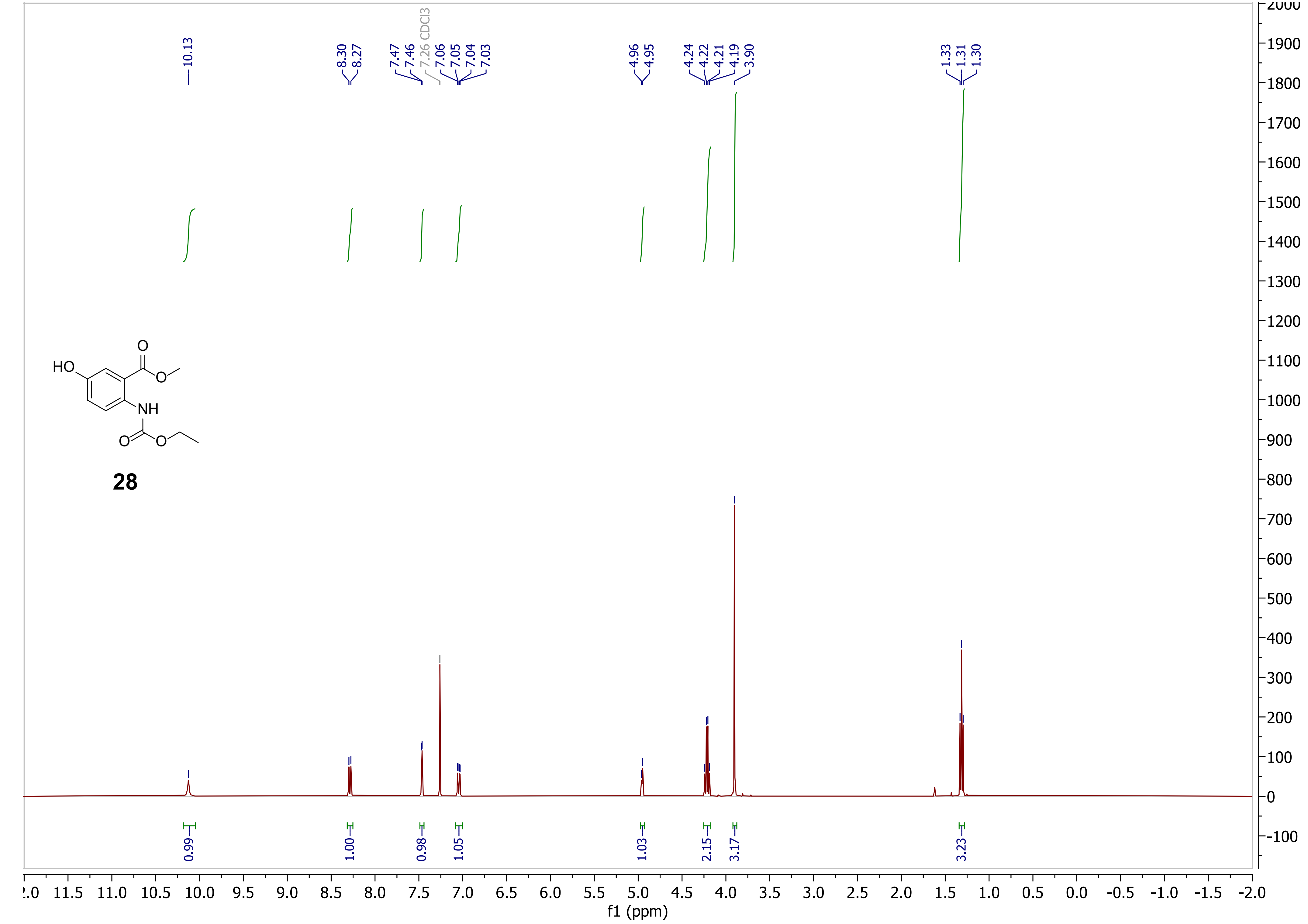

10.13
8.30
8.27
7.47
7.46
7.26 CDCl3
7.06
7.05
7.04
7.03
4.96
4.95
4.24
4.22
4.21
4.19
3.90
1.33
1.31
1.30
HO
O
O
NH
O
O
28
0.99
1.00
0.98
1.05
1.03
2.15
3.17
3.23
f1 (ppm)

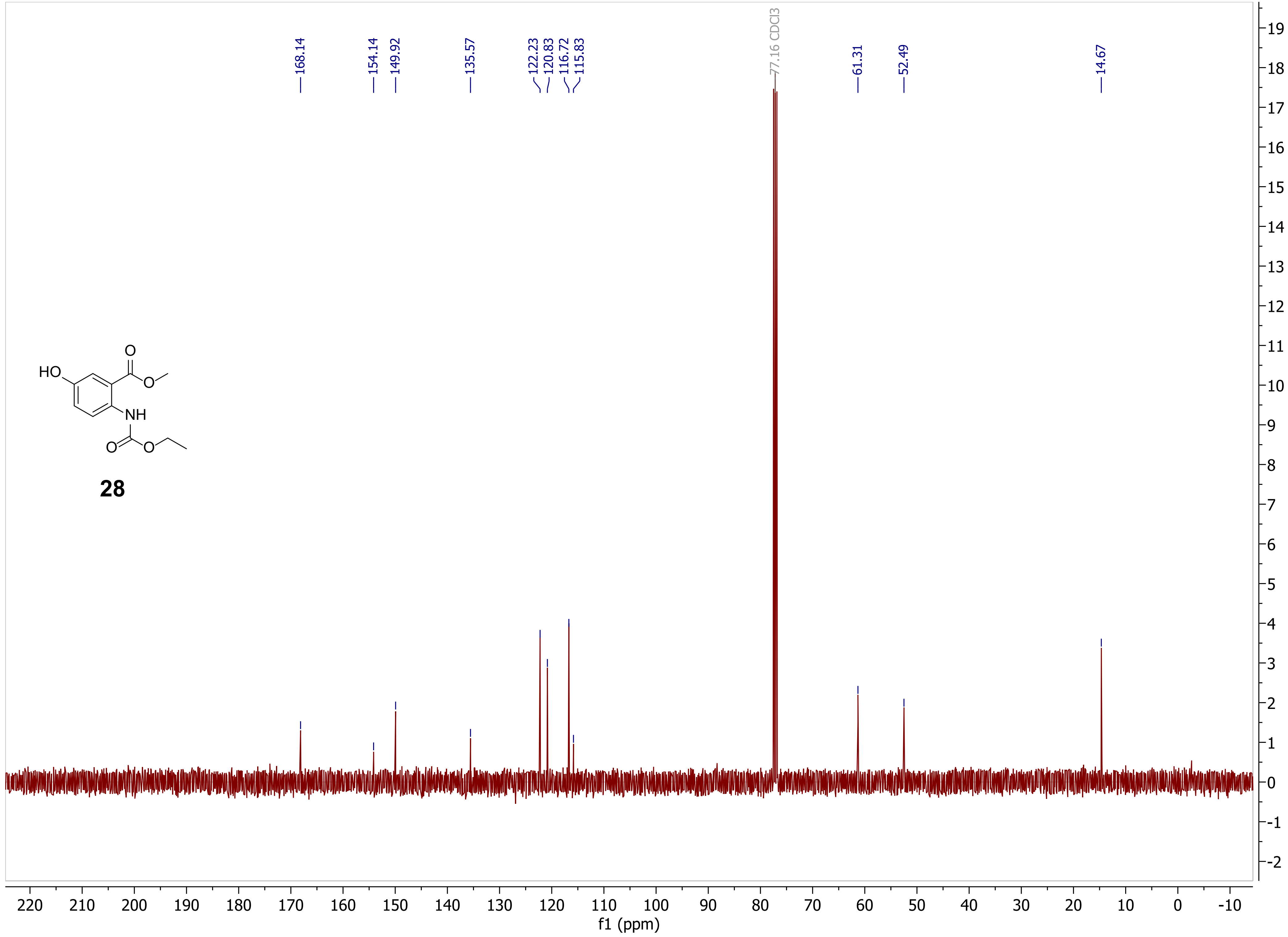

168.14
154.14
149.92
135.57
122.23
120.83
116.72
115.83
77.16 CDCl3
61.31
52.49
14.67
HO
O
O
NH
O
O
28
f1 (ppm)

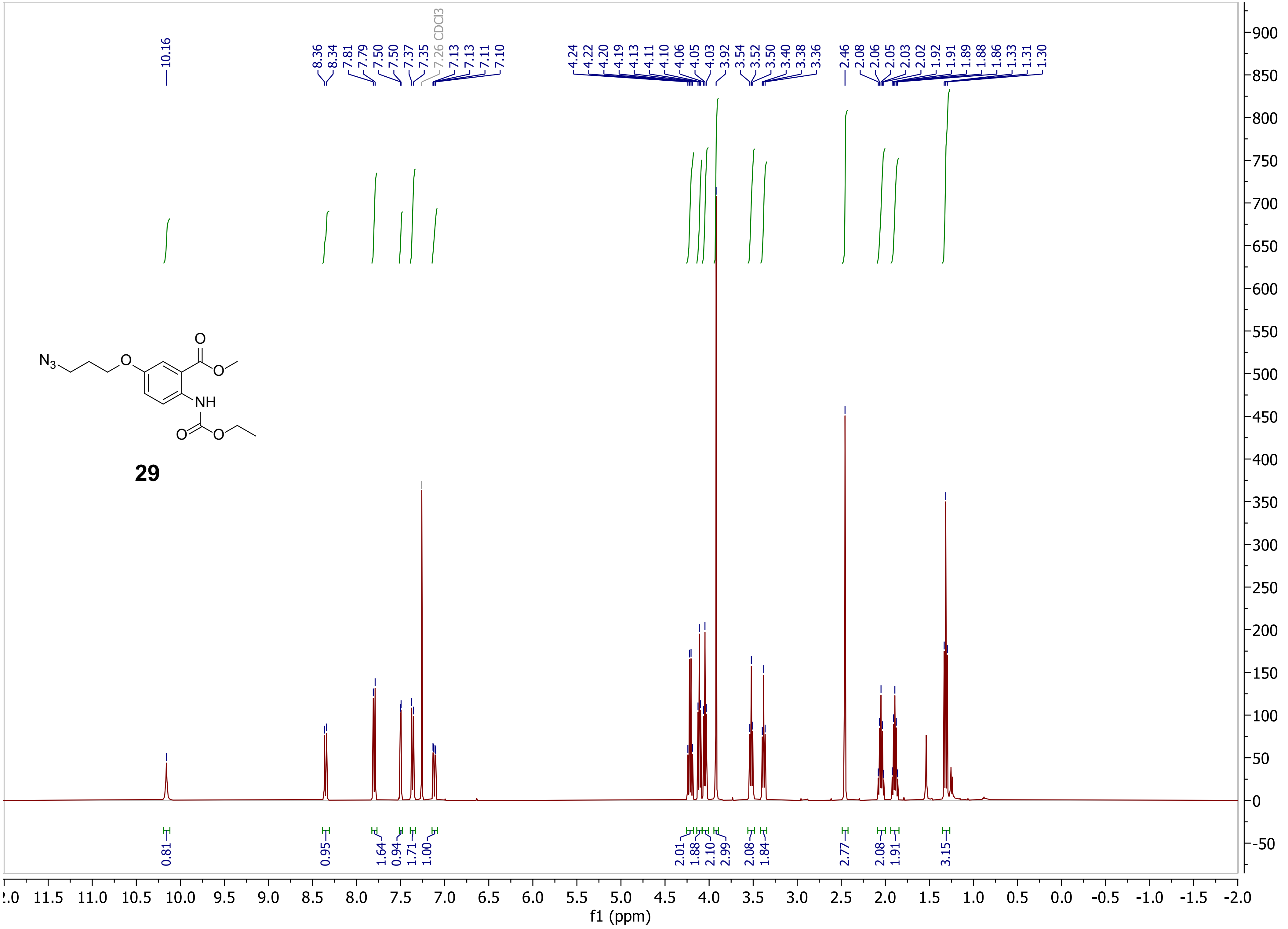
10.16
8.36 8.34 7.81 7.79 7.50 7.50 7.37 7.35 7.26 CDCl3 7.13 7.13 7.11 7.10
4.24 4.22 4.20 4.19 4.13 4.11 4.10 4.06 4.05 4.03 3.92 3.54 3.52 3.50 3.40 3.38 3.36
2.46 2.08 2.06 2.05 2.03 2.02 1.92 1.91 1.89 1.88 1.86 1.33 1.31 1.30
0.81 0.95 1.64 0.94 1.71 1.00 2.01 1.88 2.10 2.99 2.08 1.84 2.77 2.08 1.91 3.15
N3
O
NH
29
f1 (ppm)

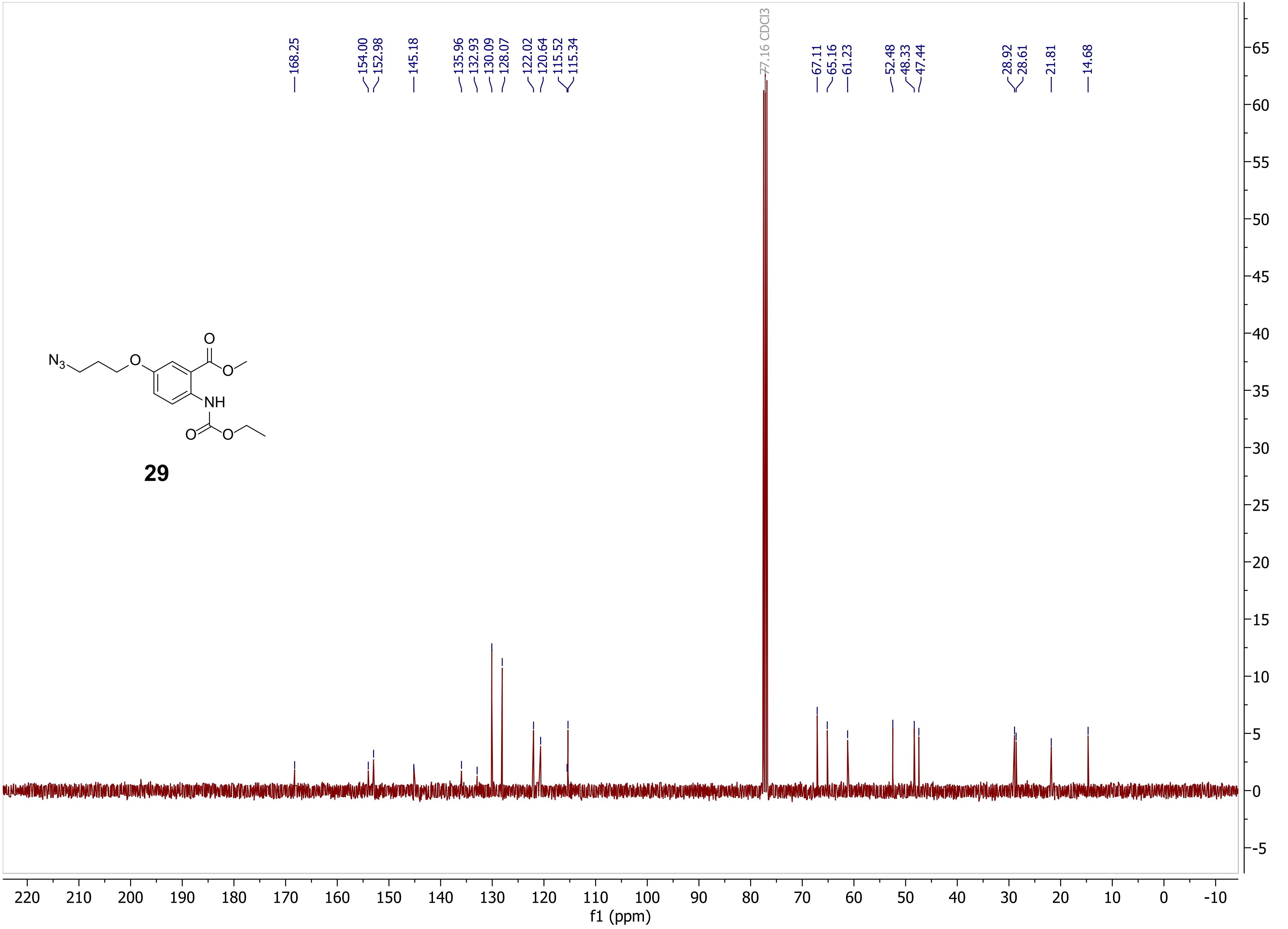
168.25
154.00
152.98
145.18
135.96
132.93
130.09
128.07
122.02
120.64
115.52
115.34
77.16 CDCl3
67.11
65.16
61.23
52.48
48.33
47.44
28.92
28.61
21.81
14.68
N3
O
O
O
NH
O
O
29
f1 (ppm)

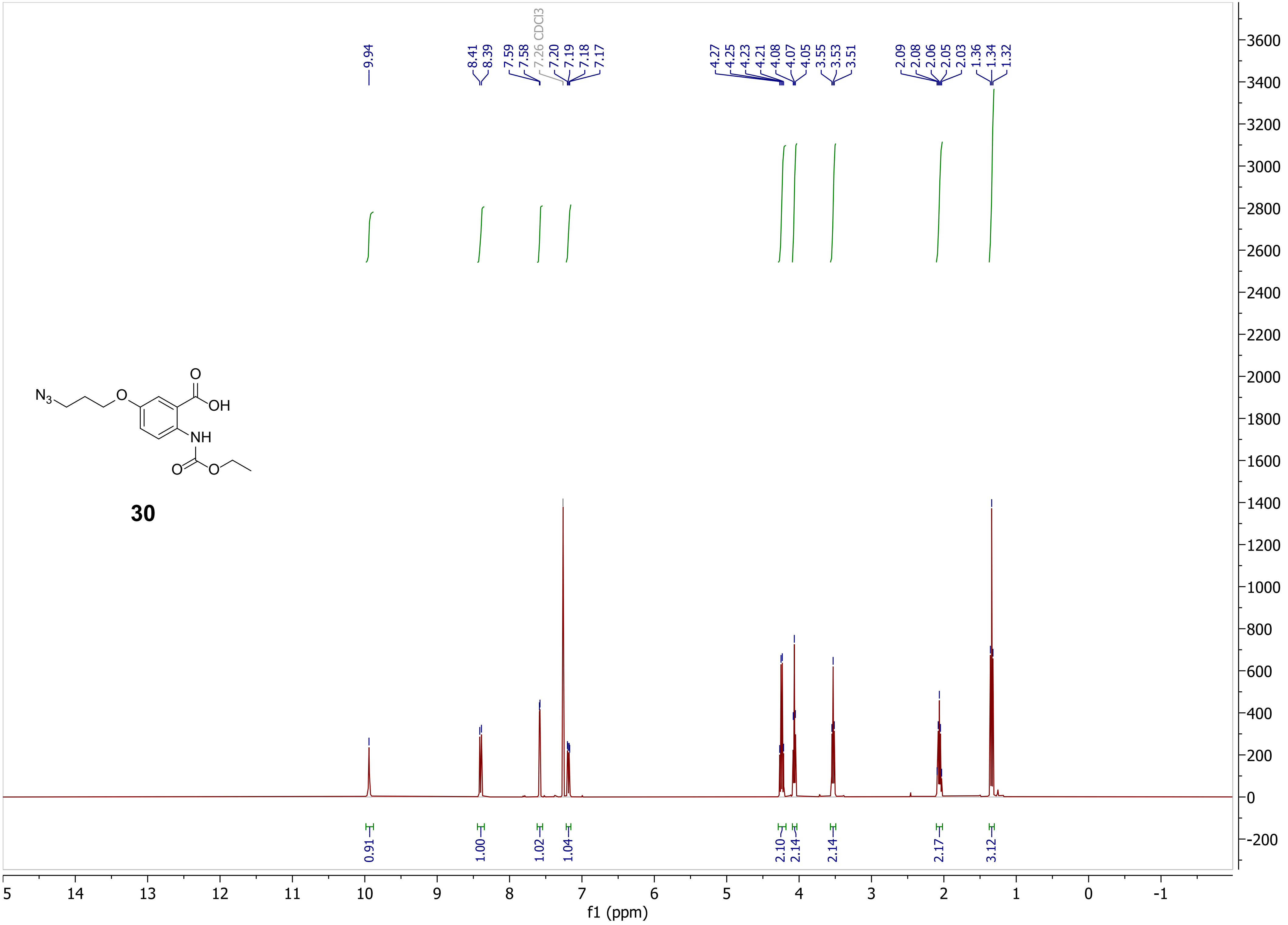

9.94
8.41
8.39
7.59
7.58
7.26 CDCl3
7.20
7.19
7.18
7.17
4.27
4.25
4.23
4.21
4.08
4.07
4.05
3.55
3.53
3.51
2.09
2.08
2.06
2.05
2.03
1.36
1.34
1.32
0.91
1.00
1.02
1.04
2.10
2.14
2.14
2.17
3.12
N3
O
OH
NH
30
f1 (ppm)

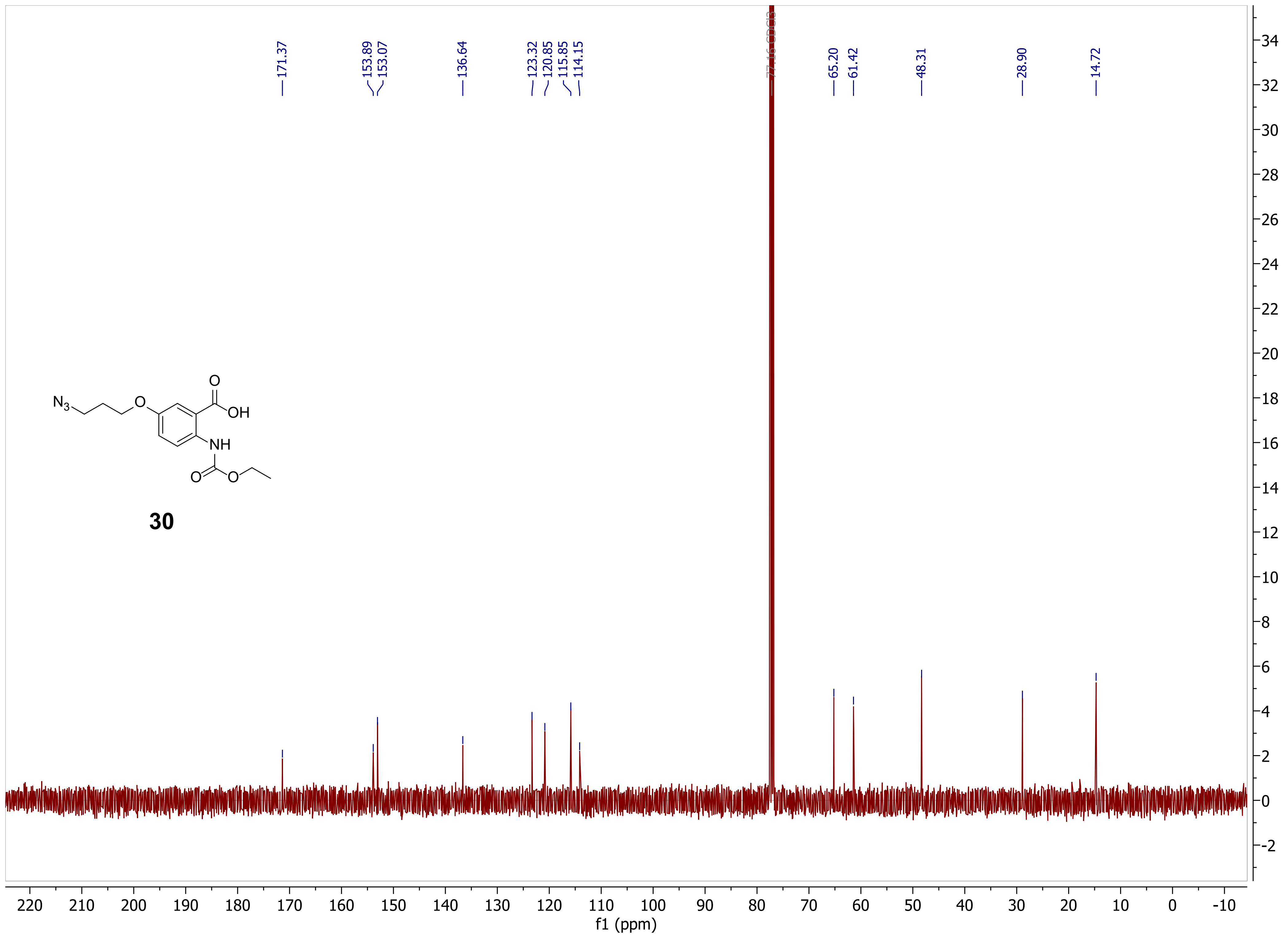

171.37
153.89
153.07
136.64
123.32
120.85
115.85
114.15
77.16 CDCl3
65.20
61.42
48.31
28.90
14.72
N3
O
O
OH
NH
O
O
30
f1 (ppm)
220 210 200 190 180 170 160 150 140 130 120 110 100 90 80 70 60 50 40 30 20 10 0 -10
34 32 30 28 26 24 22 20 18 16 14 12 10 8 6 4 2 0 -2

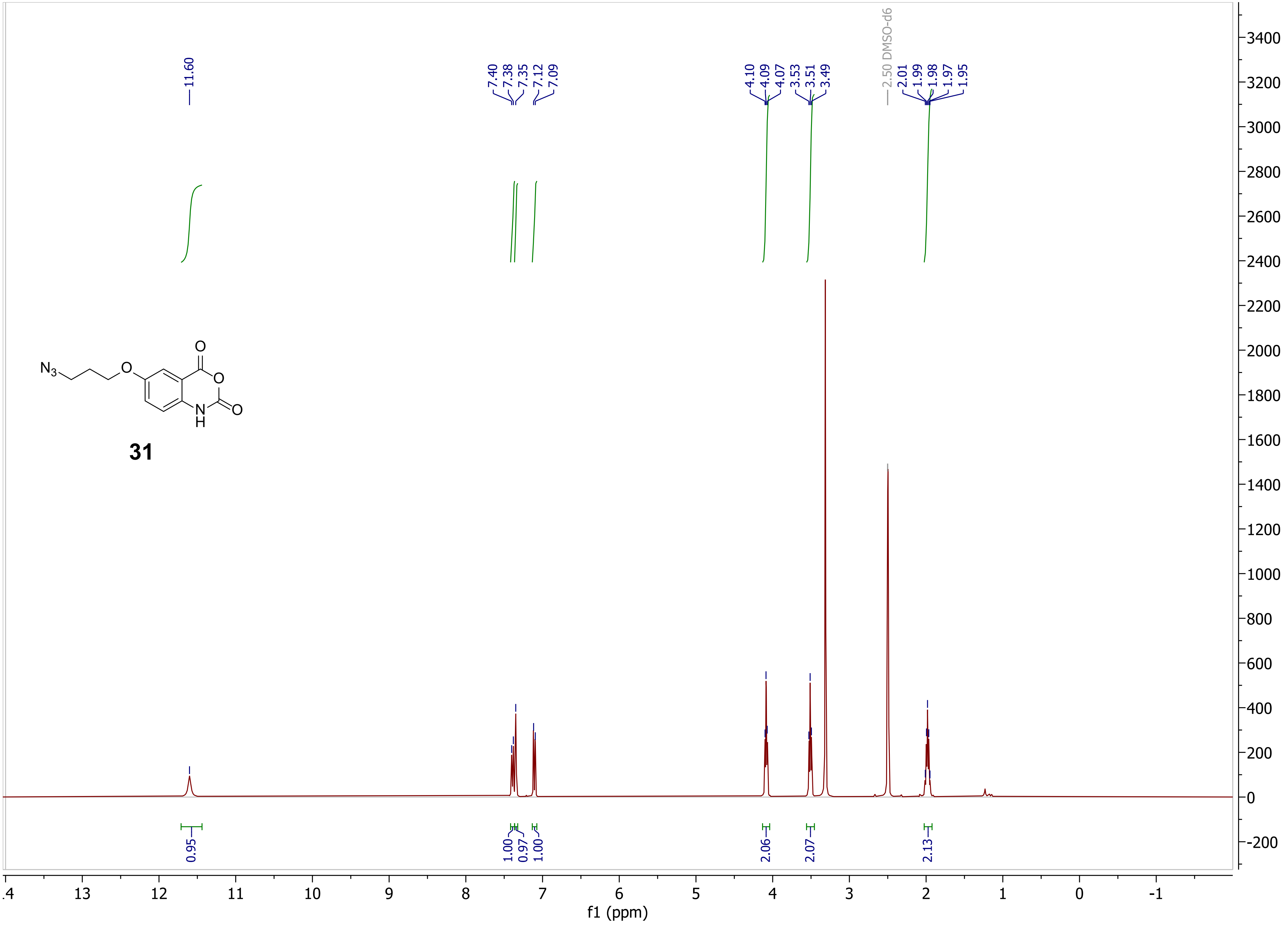

11.60
7.40
7.38
7.35
7.12
7.09
4.10
4.09
4.07
3.53
3.51
3.49
2.50 DMSO-d6
2.01
1.99
1.98
1.97
1.95
0.95
1.00
0.97
1.00
2.06
2.07
2.13
N3
O
O
O
N
H
O
31
f1 (ppm)

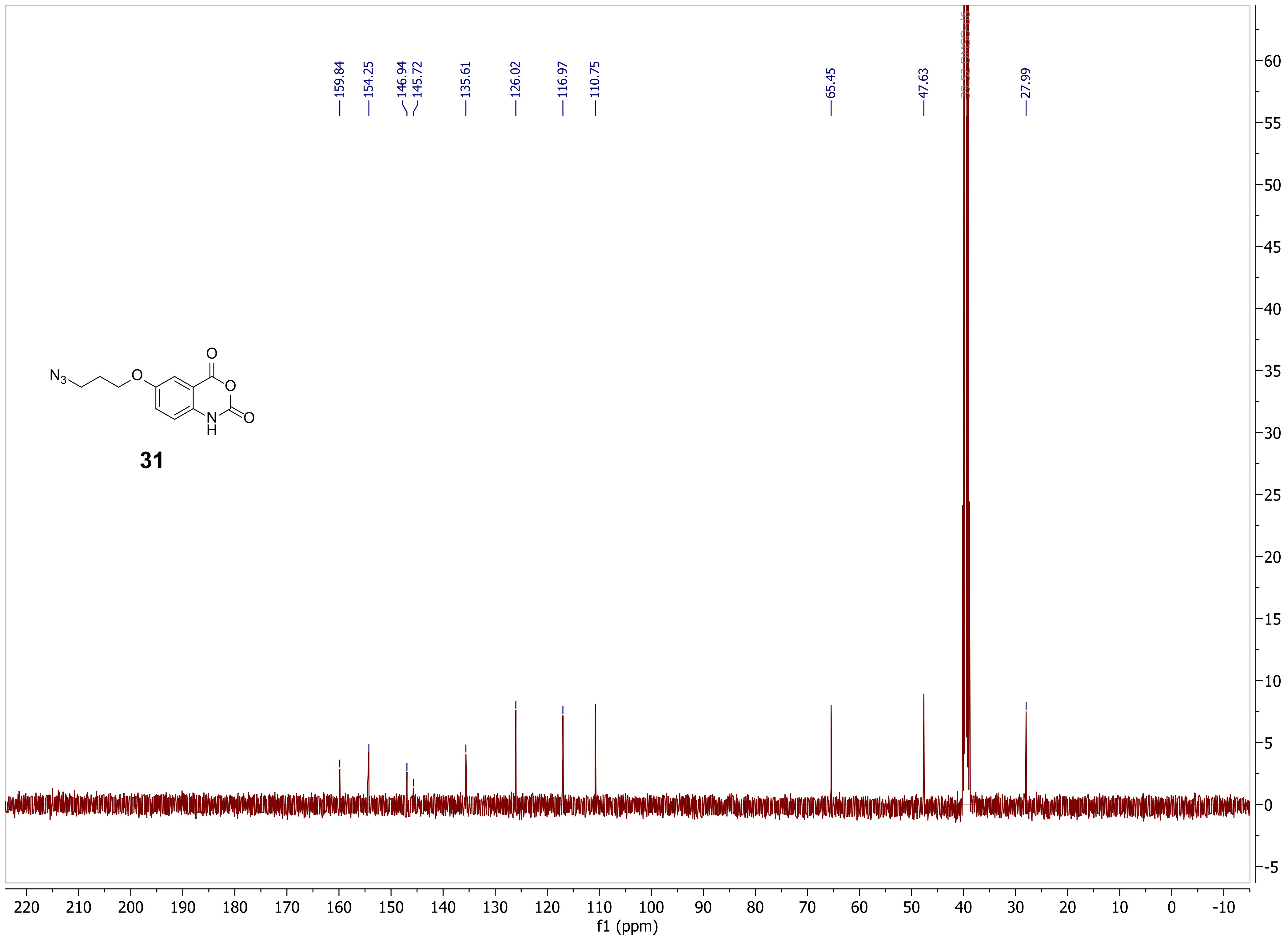

159.84
154.25
146.94
145.72
135.61
126.02
116.97
110.75
65.45
47.63
27.99
31
f1 (ppm)

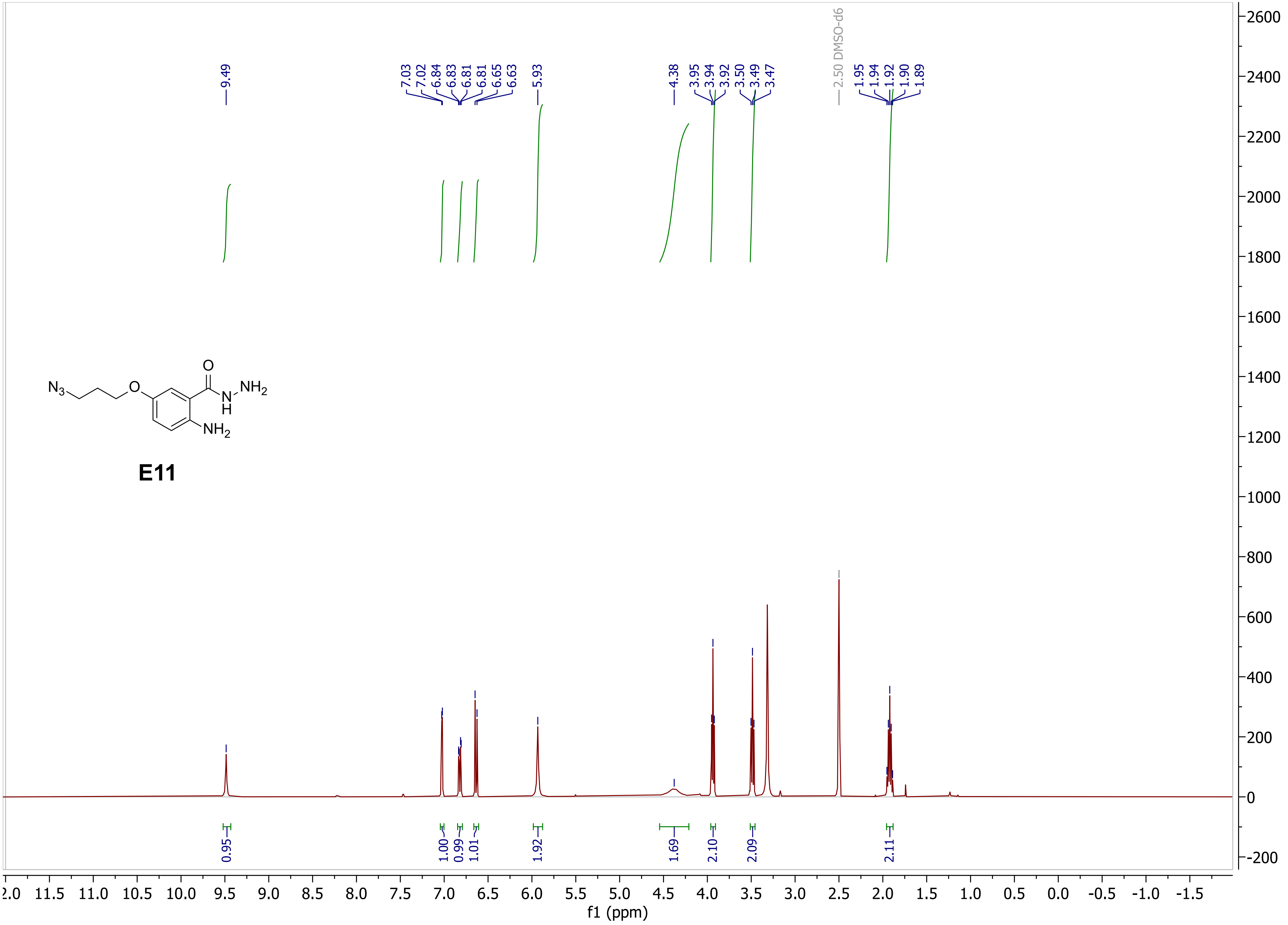

9.49
7.03
7.02
6.84
6.83
6.81
6.81
6.65
6.63
5.93
4.38
3.95
3.94
3.92
3.50
3.49
3.47
2.50 DMSO-d6
1.95
1.94
1.92
1.90
1.89
0.95
1.00
0.99
1.01
1.92
1.69
2.10
2.09
2.11
N3
O
NH2
N
H
NH2
E11
f1 (ppm)

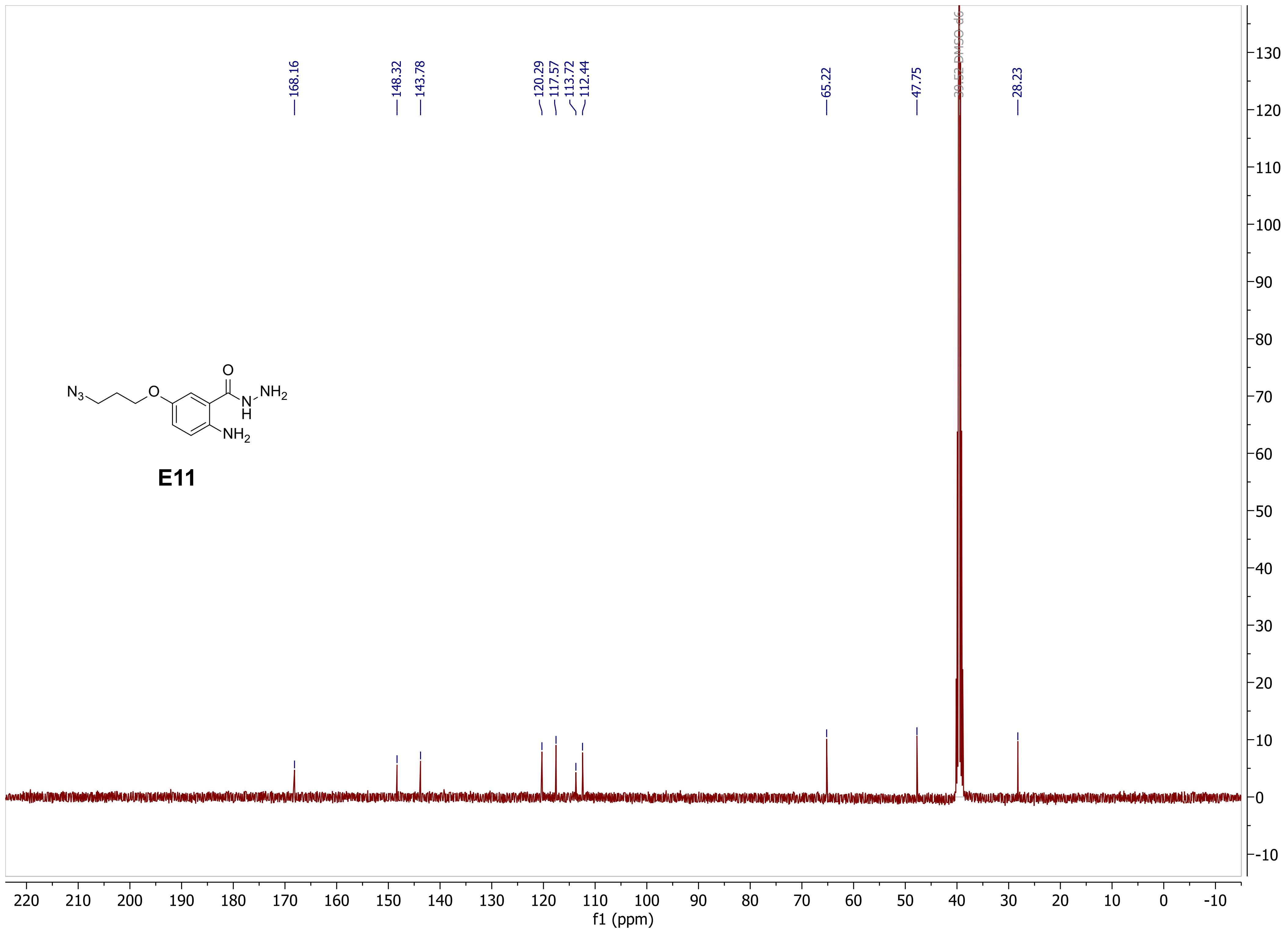
168.16
148.32
143.78
120.29
117.57
113.72
112.44
65.22
47.75
39.52 DMSO-d6
28.23
E11
N3
O
NH2
N
H
NH2
f1 (ppm)
220 210 200 190 180 170 160 150 140 130 120 110 100 90 80 70 60 50 40 30 20 10 0 -10
130 120 110 100 90 80 70 60 50 40 30 20 10 0 -10
